# Decline in attention-deficit hyperactivity disorder traits over the life course in the general population: trajectories across five population birth cohorts spanning ages 3 to 45 years

**DOI:** 10.1093/ije/dyac049

**Published:** 2022-04-11

**Authors:** Robyn E Wootton, Lucy Riglin, Rachel Blakey, Jessica Agnew-Blais, Arthur Caye, Tim Cadman, Alexandra Havdahl, Helen Gonçalves, Ana M B Menezes, Fernando C Wehrmeister, Kaili Rimfeld, George Davey Smith, Thalia C Eley, Luis Augusto Rohde, Louise Arseneault, Terrie E Moffitt, Evie Stergiakouli, Anita Thapar, Kate Tilling

**Affiliations:** MRC Integrative Epidemiology Unit, University of Bristol, Bristol, UK; Population Health Sciences, Bristol Medical School, University of Bristol, Bristol, UK; Nic Waals Institute, Lovisenberg Diaconal Hospital, Oslo, Norway; Division of Psychological Medicine and Clinical Neurosciences, MRC Centre for Neuropsychiatric Genetics and Genomics, Cardiff University, Cardiff, UK; Wolfson Centre for Young People’s Mental Health, Cardiff University, UK; MRC Integrative Epidemiology Unit, University of Bristol, Bristol, UK; Population Health Sciences, Bristol Medical School, University of Bristol, Bristol, UK; Department of Psychology, School of Biological and Chemical Science, Queen Mary University of London, London, UK; Department of Psychiatry, School of Medicine, Universidade Federal do Rio Grande do Sul, Porto Alegre, Brazil; National Institute of Developmental Psychiatry for Children and Adolescents, Brazil; ADHD and Developmental Psychiatry Programs, Hospital de Clínicas de Porto Alegre, Universidade Federal do Rio Grande do Sul, Porto Alegre, Brazil; MRC Integrative Epidemiology Unit, University of Bristol, Bristol, UK; Population Health Sciences, Bristol Medical School, University of Bristol, Bristol, UK; Nic Waals Institute, Lovisenberg Diaconal Hospital, Oslo, Norway; Department of Mental Disorders, Norwegian Institute of Public Health, Oslo, Norway; PROMENTA Research Centre, Department of Psychology, University of Oslo, Oslo, Norway; Postgraduate Program in Epidemiology, Universidade Federal de Pelotas, Pelotas, Brazil; Postgraduate Program in Epidemiology, Universidade Federal de Pelotas, Pelotas, Brazil; Postgraduate Program in Epidemiology, Universidade Federal de Pelotas, Pelotas, Brazil; Social, Genetic, and Developmental Psychiatry Centre, Institute of Psychiatry, Psychology, and Neuroscience, King’s College London, London, UK; Department of Psychology, Royal Holloway University of London, London, UK; MRC Integrative Epidemiology Unit, University of Bristol, Bristol, UK; Population Health Sciences, Bristol Medical School, University of Bristol, Bristol, UK; Social, Genetic, and Developmental Psychiatry Centre, Institute of Psychiatry, Psychology, and Neuroscience, King’s College London, London, UK; National Institute of Developmental Psychiatry for Children and Adolescents, Brazil; ADHD and Developmental Psychiatry Programs, Hospital de Clínicas de Porto Alegre, Universidade Federal do Rio Grande do Sul, Porto Alegre, Brazil; Social, Genetic, and Developmental Psychiatry Centre, Institute of Psychiatry, Psychology, and Neuroscience, King’s College London, London, UK; Social, Genetic, and Developmental Psychiatry Centre, Institute of Psychiatry, Psychology, and Neuroscience, King’s College London, London, UK; De partment of Psychology and Neuroscience, Duke University, Durham, NC, USA; MRC Integrative Epidemiology Unit, University of Bristol, Bristol, UK; Population Health Sciences, Bristol Medical School, University of Bristol, Bristol, UK; Division of Psychological Medicine and Clinical Neurosciences, MRC Centre for Neuropsychiatric Genetics and Genomics, Cardiff University, Cardiff, UK; Wolfson Centre for Young People’s Mental Health, Cardiff University, UK; MRC Integrative Epidemiology Unit, University of Bristol, Bristol, UK; Population Health Sciences, Bristol Medical School, University of Bristol, Bristol, UK

**Keywords:** Attention-deficit hyperactivity disorder, ADHD, neurodevelopment, trajectories, ALSPAC, TEDS, E-Risk, Pelotas, Dunedin

## Abstract

**Background:**

Trajectories of attention-deficit hyperactivity disorder (ADHD) traits spanning early childhood to mid-life have not been described in general populations across different geographical contexts. Population trajectories are crucial to better understanding typical developmental patterns.

**Methods:**

We combined repeated assessments of ADHD traits from five population-based cohorts, spanning ages 3 to 45 years. We used two measures: (i) the Strengths and Difficulties Questionnaire (SDQ) hyperactive-inattentive subscale (175 831 observations, 29 519 individuals); and (ii) scores from DSM-referenced scales (118 144 observations, 28 685 individuals). Multilevel linear spline models allowed for non-linear change over time and differences between cohorts and raters (parent/teacher/self).

**Results:**

Patterns of age-related change differed by measure, cohort and country: overall, SDQ scores decreased with age, most rapidly declining before age 8 years (-0.157, 95% CI: -0.170, -0.144 per year). The pattern was generally consistent using DSM scores, although with greater between-cohort variation. DSM scores decreased most rapidly between ages 14 and 17 years (-1.32%, 95% CI: -1.471, -1.170 per year). Average scores were consistently lower for females than males (SDQ: -0.818, 95% CI: -0.856, -0.780; DSM: -4.934%, 95% CI: -5.378, -4.489). This sex difference decreased over age for both measures, due to an overall steeper decrease for males.

**Conclusions:**

ADHD trait scores declined from childhood to mid-life, with marked variation between cohorts. Our results highlight the importance of taking a developmental perspective when considering typical population traits. When interpreting changes in clinical cohorts, it is important to consider the pattern of expected change within the general population, which is influenced by cultural context and measurement.

Key Messages

- This is the largest assessment to date of attention-deficit hyperactivity disorder (ADHD) trait trajectories in the general population. Our trajectories span ages 3 to 45 years and capture different geographical contexts (United Kingdom, New Zealand, Brazil).
- Overall, we observed a decline in ADHD trait scores from childhood to mid-life, with marked variation between cohorts.
- The complex patterns of change observed in the general population must be considered when interpreting changes in clinical cohorts.

## Introduction

Attention-deficit hyperactivity disorder (ADHD) is a neurodevelopmental condition-defined by a persistent and impairing pattern of inattentive, hyperactive and impulsive behaviours that typically starts in childhood.^1^ Its estimated prevalence worldwide is 3.4% (95% CI: 2.6, 4.5) in children and adolescents^2^ and 2.6% in adults (95% CI: 2.1, 3.1).^3^ The developmental course of ADHD within clinical samples is well documented: traits typically decline with age but not for all.^1^^,^^4^ Meta-analyses suggest over 80% of those with a childhood ADHD diagnosis do not meet full diagnostic criteria in adulthood, although around 65% do experience residual traits and impairment.^4^ Based upon the meta-analysed rate of decline, for an individual with ADHD there is an 83% chance of meeting full ADHD criteria 1 year later and a 96% chance of meeting residual criteria. However, trait trajectories among adults with ADHD are also highly heterogeneous.^5^

Categorical ADHD diagnosis represents one extreme of an underlying continuous distribution of ADHD traits within the general population.^6^^,^^7^ Compared with clinically ascertained samples, less is known about the developmental pattern of ADHD traits in the general population, especially into adult life. Previous cohort studies of ADHD trait trajectories suggest that for most individuals, traits are consistently low or decline across childhood/adolescence.^8^^,^^9^ However, modelling trajectories are often disrupted by the transition to adulthood, because measures and raters typically change (e.g. from parent- to self-ratings). Trajectory modelling across the life course is needed to understand the developmental course of ADHD traits in the general population: this is an important first step towards delineating what is developmentally (in)appropriate at different ages.

Even less is known about how the developmental patterns differ across different countries and cultural contexts. In this study, we use repeated measures from five population cohorts in the UK, New Zealand and Brazil, to better understand the natural history of ADHD traits in the general population. We set out to describe typical trajectories from childhood (earliest age 3 years) into mid-life (latest age 45 years) and to examine how these vary by cohort, rater, sex and common risk factors. We included repeated measures across multiple cohorts and raters through multilevel modelling, to maximize the generalizability of results: an approach previously applied to height and weight,^10^ blood pressure^11^ and alcohol consumption.^12^

## Methods

### Sample

We used data from five population-based birth cohorts: the Avon Longitudinal Study of Parents and Children (ALSPAC),^13–15^ the Twins Early Development Study (TEDS),^16^^,^^17^ the Environmental Risk (E-Risk) Longitudinal Twin Study,^18^ the Dunedin Multidisciplinary Health and Development Study^19^ and the 1993 Pelotas birth cohort.^20^ E-Risk was originally drawn from the TEDS sample, so overlapping participants were included in E-Risk only. For cohort descriptions see Table 1; also Supplementary Note 1 and Supplementary Tables S1 and S2 (available as Supplementary data at *IJE* online).

**Table 1:** Cohort descriptions and summary of attention-deficit hyperactivity disorder (ADHD) measures collected

	ALSPAC	TEDS	E-Risk	Dunedin	Pelotas
Represented population	Greater Avon region, UK	England and Wales, UK	England and Wales, UK	Greater Dunedin region, New Zealand	Pelotas, Brazil
Total sample *N*	15 645	25 656	2232	1037	5249
Analysis *N*	8959 SDQ	16 223 SDQ	–	–	4337 SDQ
	8175 DSM	14 041 DSM	2060 DSM	892 DSM	3517 DSM
*N* occasions	11 SDQ	14 SDQ			3 SDQ
	7 DSM	8 DSM	10 DSM	12 DSM	1 DSM
*N* observations	55 550 SDQ	107 613 SDQ	–	–	12 668 SDQ
	30 859 DSM	55 754 DSM	18 687 DSM	9327 DSM	3517 DSM
% female	49%	50%	51%	48%	50%
Year(s) of birth	1991-92	1994-96	1994-95	1972-73	1993
Age range	4–27 years	3–25 years	5–18 years	9–45 years	11–23 years
Relatedness	203 sibling pairs within full sample	Twins	Same-sex twins	–	–

Analysis *N* = number of people used in final analysis with at least one measure of ADHD and complete covariate data. *N* occasions = number of time points where ADHD traits were measured. Each rater at each time point is counted as a separate occasion. See Supplementary Note 2 (available as Supplementary data at *IJE* online) for more details. *N* observations = number of observations of ADHD traits available after restricting to complete covariate data.

ALSPAC, Avon Longitudinal Study of Parents and Children; TEDS, Twins Early Development Study; E-Risk, Environmental Risk Longitudinal Twin Study; Dunedin, the Dunedin Multidisciplinary Health and Development Study; Pelotas, the 1993 Pelotas birth cohort. SDQ, Strengths and Difficulties Questionnaire; DSM, Diagnostic and Statistical Manual 5th Edition criteria for ADHD.

### Measures of ADHD traits

Seven different measures of ADHD traits were available across cohorts and harmonized into two groups: (i) the hyperactive-inattentive subscale of the Strengths and Difficulties Questionnaire (SDQ); and (ii) ADHD scores based on the 18 ADHD diagnostic criteria in the Diagnostic and Statistical Manual (DSM percentage scores, see below). Parent-, teacher- and self-ratings of these measures were collected. For a detailed overview of the measures, see Supplementary Notes 2 and 3 (available as Supplementary data at *IJE* online).

*Strengths and Difficulties Questionnaire (SDQ)* measures were collected in three cohorts: ALSPAC (4–25 years), TEDS (3–21 years) and Pelotas (11 and 15 years). The hyperactive-inattentive subscale of the SDQ consists of five items capturing inattentive, hyperactive and impulsive traits. Possible scores range from 0 to 10, where higher scores represent higher ADHD trait levels. Further details of items and validations can be found in Supplementary Note 4 (available as Supplementary data at *IJE* online).

*DSM percentage scores* were measured in all five cohorts: ALSPAC (8–25 years), TEDS (8–21 years), E-Risk (5–18 years), Dunedin (9–45 years), Pelotas (21–22 years). DSM assessments ranged from 11 to 27 items and response categories from 0–1 to 0–3, resulting in considerable variation in possible scores across cohorts and across occasions (see Supplementary Notes 2, 3 and 6, available as Supplementary data at *IJE* online). To enable comparison despite this variation, scores were converted to a percentage of the total possible score for each cohort at each time point (additional information in Supplementary Note 4).

### Measures of covariates

The association between ADHD and related risk factors differs across cohorts,^21^ so we examined cohort differences by including common risk factors for ADHD as covariates in our models and allowed for interactions between covariates and cohorts. The five covariates included were: sex,^22^ birthweight (kg),^23^ gestational age (weeks),^24^ maternal age at delivery (years)^25^ and standardized parental socioeconomic position (SEP).^26^ Covariates chosen are common risk factors for ADHD and were measured across all cohorts (see additional details of covariate measures in Supplementary Note 5, available as Supplementary data at *IJE* online). We also modelled sex-stratified and SEP-stratified trajectories.

### Statistical analysis

**Figure 1:**
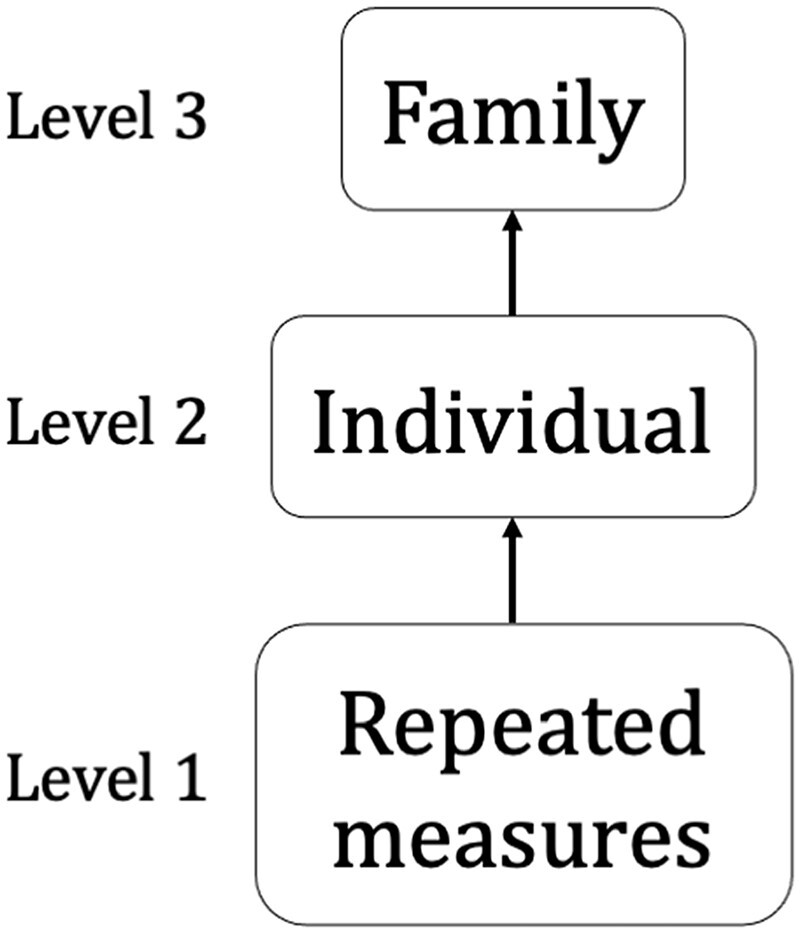
The final hierarchical multilevel model, with repeated measures of attention-deficit hyperactivity disorder traits nested within individuals who are nested within families

We used multilevel modelling (MLM) to estimate individual-specific and average trajectories of ADHD traits. We constructed separate trajectories for SDQ (3 to 25 years) and DSM (5 to 45 years) scores. We used cubic splines (smooth curves, joined at knot points, where model slope is allowed to change) and linear splines (linear periods of change, joined at knot points) to allow non-linear change over time.^10^ Model complexity was built up incrementally, beginning with parent-rated single cohorts and gradually adding additional cohorts, raters, covariates and finally related individuals. Model fit was assessed using the Akaike information criterion (AIC) and by comparing observed and predicted values for 2-year groups of age across the trajectory (see Supplementary Note 11, available as Supplementary data at *IJE* online). The final MLM is presented in Figure 1.

#### DSM benchmark model

Due to inconsistency in the assessment of DSM items (see supplementary Notes 2 and 3), we first constructed a benchmark model, removing as much variation as possible. We used only five parent-rated items that were consistent across cohorts. Differences between cohorts observed in this benchmark model can be used to help interpret the overall DSM percentage score model (see additional details in Supplementary Note 6).

#### Sensitivity analyses

We conducted MLM sensitivity analyses (i) allowing for autocorrelation, (ii) examining attrition (Supplementary Note 7, available as Supplementary data at *IJE* online), (iii) comparing centring by overall covariate mean to centring by the mean for each cohort separately and (iv) assessing the impact of zero-inflated distribution using generalized estimating equations (GEE). Even though MLM fixed effects are robust to non-normal distributions,^27^ we examined the sensitivity of our conclusions using GEE which does not rely on normality for confidence interval estimation.

## Results

### ADHD trait trajectories using SDQ scores

#### Model fitting

The best fitting model in the test cohort (ALSPAC) had linear splines with knot points at ages 8 and 16 years, where the rate of decrease changed at each knot point: the rate of decrease was shallower following the age 8 knot point and steeper again following the age 16 knot point (cubic splines, Supplementary Figure S1; fit comparisons, Supplementary Tables S3–S4, available as Supplementary data at *IJE* online). The model fit remained good after adding in additional raters and cohorts (Supplementary Figure S2 and Tables S5–S7, available as Supplementary data at *IJE* online). Mean and standard deviation for hyperactive-inattentive SDQ scores with age across cohorts were similar for ALSPAC and TEDS and slightly higher for Pelotas (Supplementary Table S8, available as Supplementary data at *IJE* online). The best fitting model was adjusted for rater, cohort, sex, birthweight, maternal age at delivery, and SEP (Supplementary Table S9, available as Supplementary data at *IJE* online). Additionally, fit was improved by interacting cohort with rater, age at delivery, SEP and slope (final model fit, Supplementary Table S10, available as Supplementary data at *IJE* online), suggesting that these can partially account for the observed differences in scores between cohorts.

**Figure 2:**
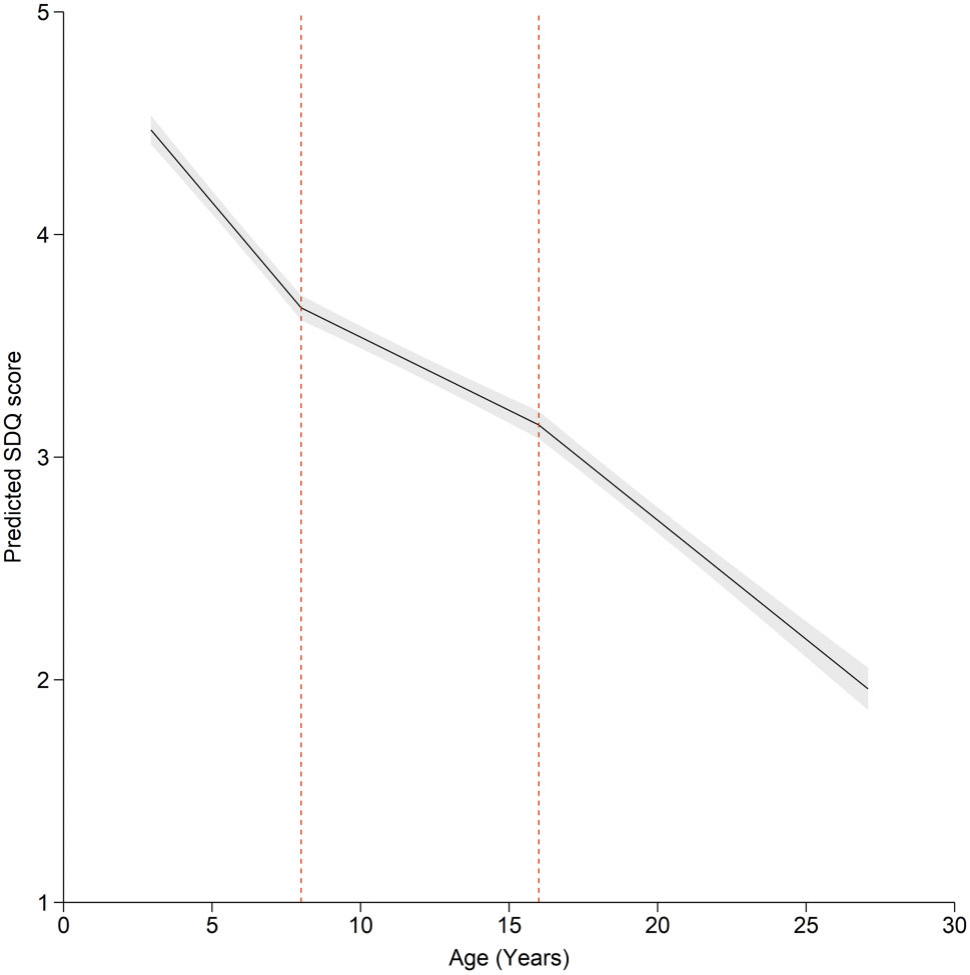
The best-fitting model of Strengths and Difficulties Questionnaire hyperactive-inattentive subscale scores with knot points at 8 and 16 years, using data from three cohorts combined (the Avon Longitudinal Study of Parents and Children: ALSPAC; the Twins Early Development Study; the Pelotas 1993 birth cohort). Plotted average scores are parent-rated for a male from the ALSPAC cohort, with mean covariate values

The final model of hyperactive-inattentive SDQ scores comprised 175 831 observations from 29 519 individuals (Figure 2; Supplementary Table S11, available as Supplementary data at *IJE* online). This model estimated that a male, aged 3 years, from the ALSPAC cohort (with mean covariate values), would have an average parent-rated hyperactive-inattentive SDQ score of 4.46 (95% CI: 4.40, 4.53). Average hyperactive-inattentive SDQ score decreased by 0.16 (95% CI: -0.17, -0.14) per year between ages 3 and 8 years; by 0.07 (95% CI: -0.08, -0.06) per year between ages 8 and 16 years; and by 0.11 (95% CI: -0.12, -0.10) per year after age 16. Average hyperactive-inattentive SDQ scores were 0.82 (95% CI: -0.86, -0.78) lower for females than males (sex-stratified results, Supplementary Note 8, available as Supplementary data at *IJE* online). Per SD increase in SEP, average hyperactive-inattentive SDQ scores were reduced by -0.15 (95% CI: -0.18, -0.11) (SEP-stratified results, Supplementary Note 9, available as Supplementary data at *IJE* online). Results were similar to those where covariates were centred at the mean for each cohort (Supplementary Table S11) and fixed effects were consistent with a model allowing for autocorrelation.

#### Cohort comparisons

**Figure 3:**
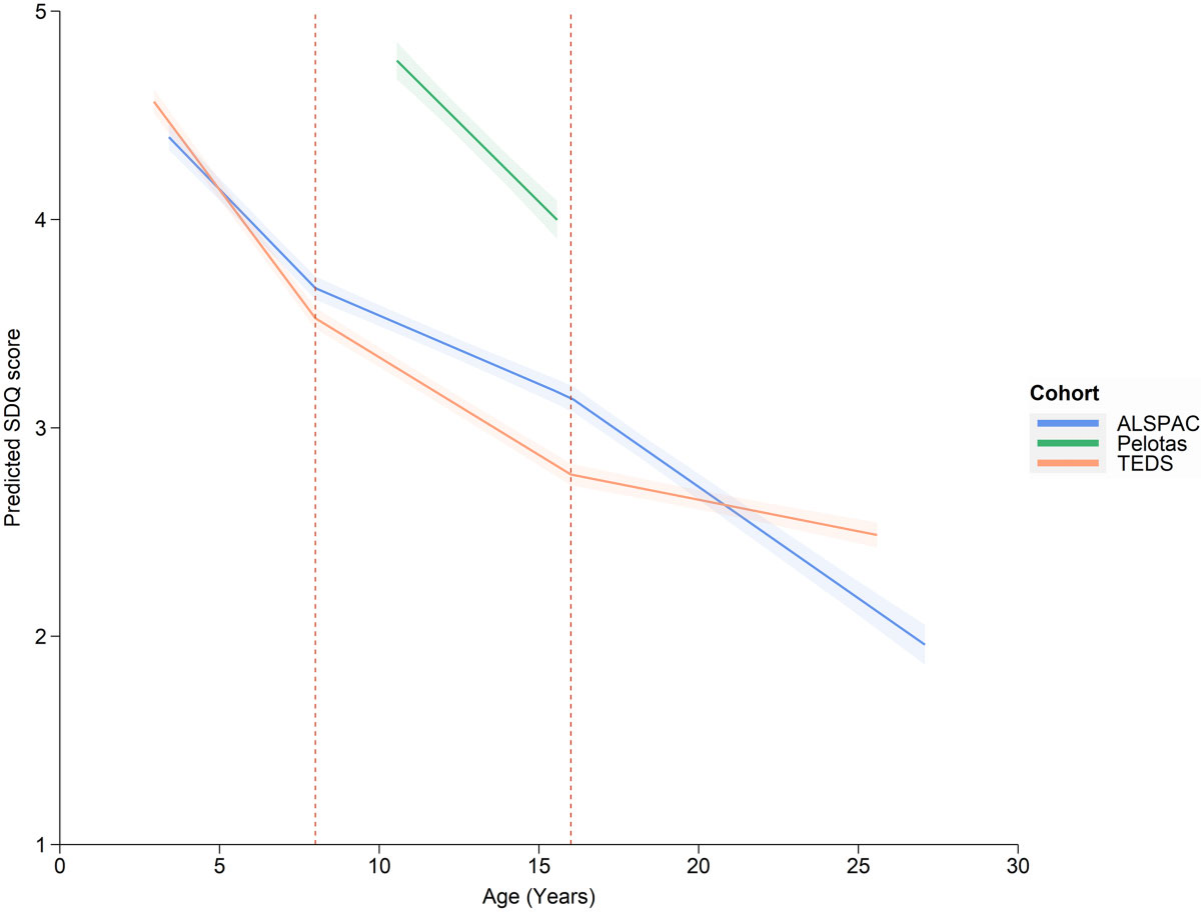
The best-fitting model of Strengths and Difficulties Questionnaire hyperactive-inattentive subscale scores for each cohort separately. The best-fitting model had linear splines with knot points at 8 and 16 years. Plotted average scores are parent-rated for a male, with mean covariate values

Figure 3 compares the trajectories across cohorts. Average scores for ALSPAC and TEDS were similar, with higher average scores for Pelotas. Average self-ratings were higher than parent-ratings (1.71, 95% CI: 1.62, 1.81) and teacher-ratings were lower than parent-ratings (-0.80, 95% CI: -0.84, -0.76). There was an interaction between cohort and rater, such that self-ratings were higher than parent-ratings in TEDS and ALSPAC (intercept difference = 1.02 and 1.71 hyperactive-inattentive SDQ points, respectively), but self-rated ADHD traits were lower than parent-rated in Pelotas (intercept difference = -0.98 and hyperactive-inattentive SDQ points, respectively). For full model coefficients, see Supplementary Table S11; for extrapolated trajectories, see Supplementary Figure S3 (available as Supplementary data at *IJE* online).

#### Between- and within-person variability

Of the total variation in hyperactive-inattentive SDQ scores at baseline, 53% was explained by level 1 (within-participant variation), 33% was explained by level 2 (between-participant) variation and 14% was explained by level 3 (between-family) variation. In other words, most of the variability between scores is explained by the reliability of two repeated scores within the same person; approximately a quarter is explained by differences between people; and there is very little similarity between families.

### ADHD trait trajectories using DSM percentage scores

#### Benchmark model

Average scores were lowest for ALSPAC, then TEDS, Dunedin, E-Risk and finally Pelotas which had the highest average DSM percentage scores (Supplementary Figure S4, available as Supplementary data at *IJE* online). All cohorts had similar trajectories despite different average scores, with the exception of Pelotas, which showed a steeper trajectory (additional details in Supplementary Note 6).

#### Model fitting

The best fitting model in the test cohort (TEDS) had knot points at ages 14, 17 and 21 years (cubic spline, Supplementary Figure S5; fit comparison, Supplementary Tables S16 and S17, available as Supplementary data at *IJE* online). The model fit remained good after adding in additional raters and cohorts (Supplementary Tables S18–S22, available as Supplementary data at *IJE* online). This model was adjusted for rater, cohort, sex, birthweight, gestational age, maternal age at delivery and SEP. Additionally, fit was improved by interacting cohort with sex, rater, age at delivery, SEP and slope (covariate iterative removal, Supplementary Table S23; final model fit, Supplementary Table S28, available as Supplementary data at *IJE* online).

**Figure 4:**
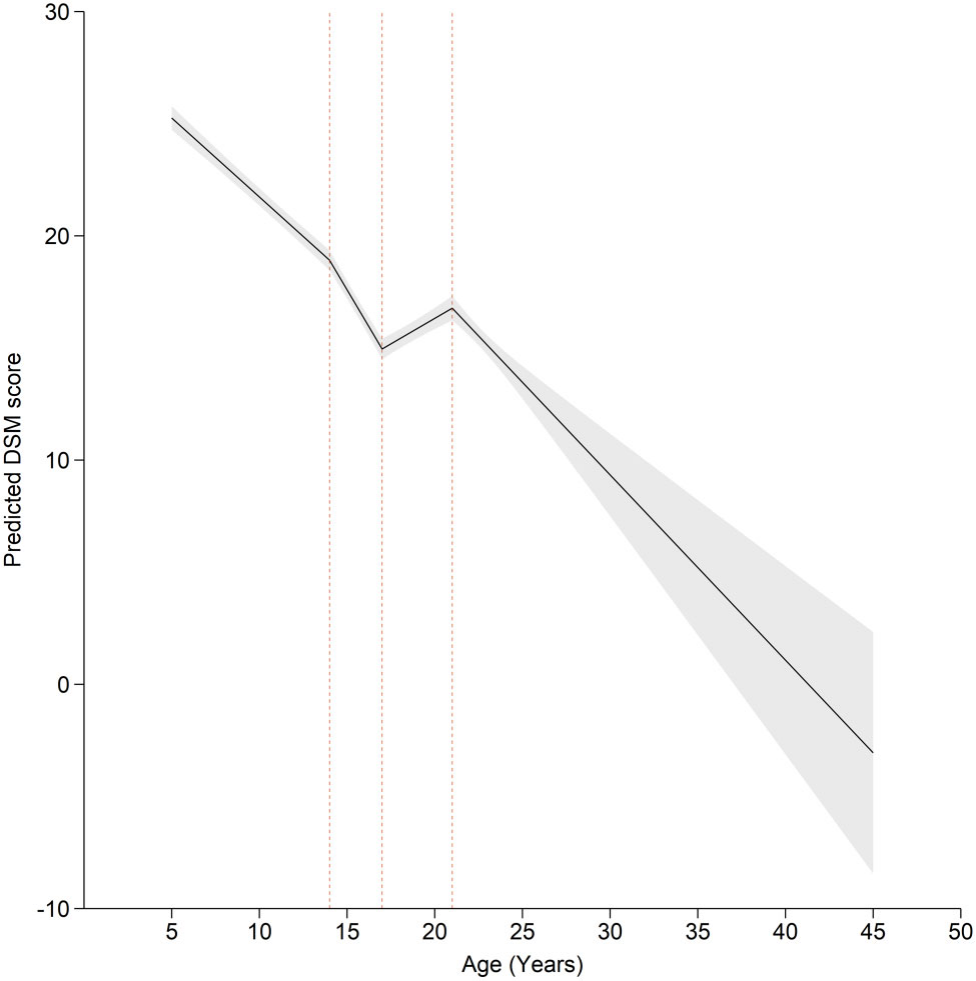
The best-fitting model of Diagnostic and Statistical Manual percentage scores. The model has linear splines with knot points at 14, 17 and 21 years, and uses data from five cohorts combined (the Twins Early Development Study: TEDS; the Avon Longitudinal Study of Parents and Children; the Pelotas 1993 birth cohort; the Environmental Risk Longitudinal Twin Study; the Dunedin Multidisciplinary Health and Development Study). Plotted average scores are parent-rated for a male, from the TEDS cohort with mean covariate values. Values below zero are not theoretically possible—the model is creating less accurate predictions at the extreme of the age distribution due to smaller numbers of observations in older ages. Values at extreme ages should be interpreted with caution

The final model of DSM percentage scores (using all available items) comprised 118 144 observations from 28 685 individuals (Figure 4;Supplementary Table S29, available as Supplementary data at *IJE* online). From ages 5 to 14 years, average DSM score decreased by 0.70% (95% CI: -0.77, -0.64) each year. From ages 14 to 17 years, average DSM score decreased by 1.32% (95% CI: -1.47, -1.17) each year. From ages 17–21, there was a small increase in average DSM score by 0.46% (95% CI: 0.32, 0.59) per year. For exploratory analysis of this increase, see Supplementary Note 10 (available as Supplementary data at *IJE* online). From 21 years onwards, average DSM scores declined by 0.83% (95% CI: -1.07, -0.59) each year. Average DSM scores were 4.93% lower (95% CI: -5.38, -4.49) for females than males (sex-stratified results, Supplementary Note 8, available as Supplementary data at *IJE* online). Per SD increase in SEP, average DSM scores were reduced by -1.72% (95% CI: -1.98, -1.46) (SEP-stratified results, Supplementary Note 9, available as Supplementary data at *IJE* online). Results were similar when covariates were centred at the mean for each cohort, using GEE (Supplementary Table S29, available as Supplementary data at *IJE* online), and fixed effects were consistent with a model allowing for autocorrelation.

#### Cohort comparisons

**Figure 5:**
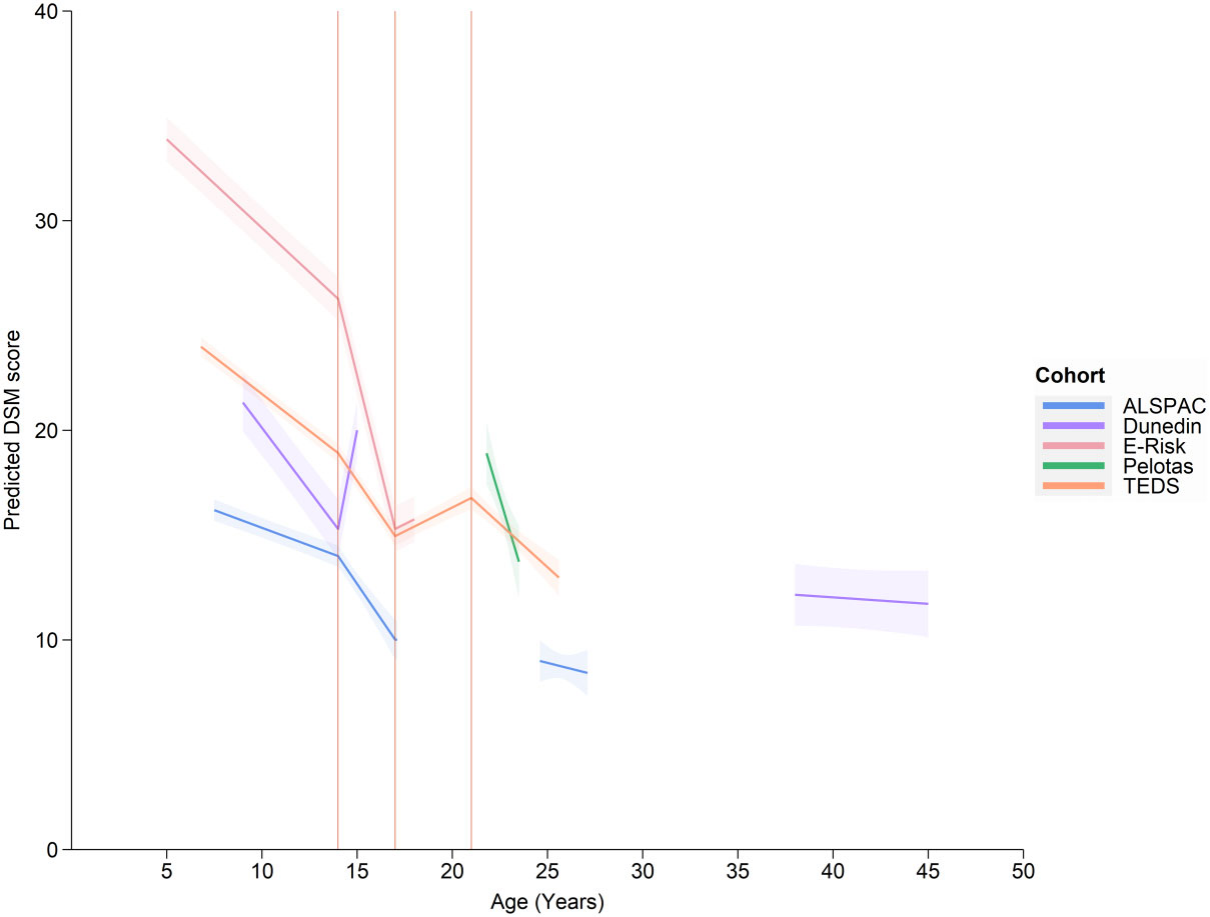
The best-fitting model of Diagnostic and Statistical Manual percentage scores for each cohort separately. The best-fitting model had knot points at 14, 17 and 21 years. Plotted average scores are parent-rated for a male with mean covariate values

A comparison of the trajectories across cohorts is shown in Figure 5. On average, self-ratings were higher than parent-ratings (10.79%; 95% CI: 10.51, 11.07) and teacher-ratings were lower than parent-ratings (-2.10%; 95% CI: -2.81, -1.39). Scores were the highest for E-Risk and the lowest for ALSPAC, especially for the first spline (intercept difference = 16.85%). Similar to the hyperactive-inattentive SDQ model, TEDS and ALSPAC had a similar slope, but average scores were higher for the TEDS cohort (intercept difference = 8.22%). Trajectories differed more across cohorts compared with the benchmark model (Supplementary Figure S4). This could suggest that some of the differences between cohorts are due to differences in DSM measurement rather than true differences in slope. For extrapolated DSM percentage scores, see Supplementary Figure S6 (available as Supplementary data at *IJE* online).

#### Between- and within-person variability

Of the total variation in DSM percentage scores at baseline, 37% was explained by level 1 (within-participant variation), 46% was explained by level 2 (between-participant) variation and 16% was explained by level 3 (between-family) variation. As with hyperactive-inattentive SDQ, similarity within families was low. However, here the variation between individuals was high and within individuals was lower than for the hyperactive-inattentive SDQ.

## Discussion

This is the most comprehensive investigation to date of the developmental course of ADHD traits from childhood to adulthood in the general population. There was an overall pattern of decreasing traits across development, which is consistent with findings from both single cohort studies spanning childhood/adolescence and clinical samples across the lifespan.^4^^,^^28^^,^^29^

Average ADHD traits differed according to sex, rater, cohort, socioeconomic position, birthweight, maternal age at delivery and gestational age. Overall, males had higher average scores than females, consistent with the well-documented preponderance for ADHD traits in boys in clinical^22^ and population samples.^30^ For both measures, this sex difference decreased over age, showing overall steeper decrease for males. Consequently by approximately age 25 years, average scores were similar for males and females. There is mixed evidence for a reduction in sex differences by adult life,^31–33^ which could reflect true differences in persistence or possibly that ADHD measures are better suited to detecting childhood traits in males than females.^34^

Average ADHD traits were highest for self-ratings and lowest for teacher-ratings, for both hyperactive-inattentive SDQ and DSM. Higher self- compared with parent-ratings have been reported in young adults without ADHD.^35^ However, this differs compared with clinical samples for which children with ADHD diagnosis tend to under-report traits compared with parents.^35–37^ Non-corroboration between raters has implications for longitudinal measurement, because respondents typically change from parent to self during adolescence. Our multilevel modelling approach accounted for this by including a fixed effect for rater and by allowing interactions between rater and cohort.

Across cohorts there were differences in average ADHD trait scores, even after controlling for rater and other covariates. For both hyperactive-inattentive SDQ and DSM models, the 1993 Pelotas cohort had higher average scores compared with ALSPAC and TEDS. This is consistent with a previous cross-cohort comparison which found higher SDQ and DAWBA scores in the 2004 Pelotas cohort compared with the ALSPAC cohort.^21^ Previous estimates of adolescent ADHD population prevalence from across Brazil were within the expected range,^38^ but estimates for ADHD in adulthood^39^ were higher than those found in the UK and New Zealand.^40–42^ It is important to note that items were translated into the Portuguese for the 1993 Pelotas cohort, which could have possibly influenced interpretation. The only cohort with higher average ADHD scores than Pelotas was E-Risk. This is likely due to the E-Risk sampling approach (a subsample of TEDS), where more young mothers were contacted and attrition was low, achieving a sample more representative of the UK population.^18^ To avoid overlap in the current study, we removed all E-Risk participants from the TEDS cohort, making it likely less representative of the general population in the UK.^17^ The higher ADHD scores observed in E-Risk likely better reflect the UK population. Furthermore, the majority of participants in the final model were from UK-based cohorts, with smaller numbers of participants from New Zealand and Brazil. Consequently, model inferences are likely to be most applicable to the UK population. Future investigations should incorporate data from additional countries to make wider generalizations.

Despite an overall decreasing trend, the average DSM percentage scores increased slightly between the ages of 17 and 21 years. This period of transition to adulthood is a particularly challenging time and a peak age for depression onset,^43^ both of which could exacerbate, and affect the measurement of, ADHD traits.^36^ However, only two of the contributing cohorts had observations of DSM-related items between these ages (TEDS and E-Risk), and both included additional self-reported items at these time points to capture age-related change in ADHD trait presentation (see Supplementary Note 10, available as Supplementary data at *IJE* online). These ADHD items were more frequently endorsed than the original items, which could suggest they are indeed more relevant to this developmental period or that they capture behaviours less specific to ADHD.^35^ We did not see an increase at this age in either our benchmark DSM model, or our model of hyperactive-inattentive SDQ, suggesting it is most likely due to different measurement rather than a true increase in average scores. This highlights the complexity of longitudinal work spanning different developmental periods: consistent measures are needed for the robust investigation of ADHD traits across age,^44^ but different measures are often needed to assess the same underlying construct in a developmentally appropriate manner. When items are adapted to be more developmentally appropriate, we recommend that the original measure is also included to allow for direct comparison.

Furthermore, caution is needed in interpreting average DSM percentage scores, given measurement variability both within and between cohorts (e.g. number of items, scoring of items, phrasing of items). These measurement differences meant that we were not able to use item counts, which would have enabled comparison between our general population trajectories and diagnostic thresholds. We converted DSM scores to the percentage of total possible scores to enable harmonization across cohorts and still observed greater cross-cohort variation for DSM percentage scores compared with hyperactive-inattentive SDQ scores. Triangulation of results with a benchmark model and the hyperactive-inattentive SDQ model enabled us to infer which changes might be due to measurement differences rather than true score change over time. Our findings highlight the importance of collecting consistent repeated measures in longitudinal cohorts to explore age-related change. This improves confidence in inferences from trajectory modelling and facilitates more effective meta-analyses across cohorts.^2^

Finally, it is important to note that the contributing cohorts suffer from non-random attrition to varying degrees,^17^^,^^20^^,^^45^ with those at the highest risk of psychopathology most likely to drop out.^45^ MLMs are robust to bias from attrition that is missing at random (i.e. observed variables predict dropout). Cohorts that have only collected measures later are therefore more likely to show bias because they do not have earlier observed scores. This could in part explain higher scores in the Pelotas cohort, as well as different sample compositions. Our inclusion of individuals with single observations will have reduced bias from attrition. Furthermore, results for individuals who had responded in early, middle and late age were very consistent with the main model, and results for E-Risk and Dunedin (where attrition was much lower) showed similar findings. However, we cannot rule out the possibility that the average reduction in ADHD traits over time could be in part due to non-random attrition.

## Conclusions

There was an overall pattern of decreasing ADHD traits across childhood through to adulthood in the general population in three different countries (UK, Brazil, New Zealand). This is the most comprehensive investigation to date of the developmental course of ADHD traits in the general population. The pattern of non-linear change was influenced by several factors including rater, sex and cohort. Our trajectories, which span childhood to mid-life in the general population, are a valuable step towards determining what is developmentally typical. We also emphasize the need for greater consistency in measurement of ADHD traits both between and within cohorts, which will improve the interpretation of future longitudinal models that aim to combine data across cohorts.

## Ethics approval

Ethical approval for the study was obtained from each cohort individually. For ALSPAC, the study was approved by the ALSPAC Ethics and Law Committee and the local research ethics committees. Informed consent for the use of data collected via questionnaires and clinics was obtained from participants following the recommendations of the ALSPAC Ethics and Law Committee at the time. TEDS and their consent procedures were approved by the King’s College London Research Ethics Committee (ref: PNM/09/10–104). For E-Risk, the Joint South London and Maudsley and the Institute of Psychiatry Research Ethics Committee approved each phase of the study. Parents gave informed consent and twins gave assent between 5 and 12 years and then informed consent at age 18. For Pelotas, ethical approval for the study was obtained from the Ethics and Research Committee of the Faculty of Medicine of the Federal University of Pelotas. Informed consent was obtained from parents, and also cohort participants gave their consent when applicable. For Dunedin, the NZ-HDEC (Health and Disability Ethics Committee) approved the study and all study members provided written informed consent.

## Data availability

The data underlying this article cannot be shared publicly. Researchers can apply for access to each of the cohorts.

## Supplementary data

Supplementary data are available at *IJE* online.

## Author contributions

A.T., K.T., E.S. and G.D.S. designed the study and obtained funding for the work. J.A.B., A.C., K.R., T.C.E., L.A.R., L.A., F.C.W., H.G., A.M.B.M. and T.E.M. provided datasets, cleaned variables and provided sample expertise. R.E.W. conducted the analysis and led the manuscript writing. L.R. and R.B. helped with analysis and script checking. T.C. helped with analysis and presenting results. K.T. planned the methodological approach and oversaw all analyses. All co-authors helped in interpreting the results and revising the manuscript. This publication is the work of the authors, but R.E.W. and K.T. will serve as guarantors for the contents of this paper. 

## Funding

The UK Medical Research Council and Wellcome (grant ref: 217065/Z/19/Z) and the University of Bristol provide core support for ALSPAC. A comprehensive list of grants funding is available on the ALSPAC website [http://www.bristol.ac.uk/alspac/external/documents/grant-acknowledgements.pdf]. This ALSPAC data collection was specifically funded by the NIH (5R01MH073842-04), the Wellcome Trust and MRC (076467/Z/05/Z) and the Wellcome Trust (204895/Z/16/Z). TEDS is supported by a programme grant to T.C.E. from the UK Medical Research Council (MR/V012878/1 and and previously G0901245), with additional support from the US National Institutes of Health (AG046938). The E-Risk Study support came from the UK Medical Research Council (grant G1002190), the US National Institute of Child Health and Development (grant HD077482) and the Klaus J. Jacobs Foundation. The ‘Pelotas Birth Cohort, 1993’ is conducted by the Postgraduate Program in Epidemiology at Universidade Federal de Pelotas with the collaboration of the Brazilian Public Health Association (ABRASCO). From 2004 to 2013, the Wellcome Trust supported the 1993 birth cohort study. The European Union, National Support Program for Centers of Excellence (PRONEX), the Brazilian National Research Council (CNPq) and the Brazilian Ministry of Health supported previous phases of the study. The 22-year follow-up was supported by the Science and Technology Department/Brazilian Ministry of Health, with resources transferred through the Brazilian National Council for Scientific and Technological Development (CNPq) (grant 400943/2013–1). Dunedin Study support came from the US-National Institute on Aging (grants AG032282 and AG069939) and UK Medical Research Council (grant MR/P005918/1). The Dunedin Multidisciplinary Health and Development Research Unit is supported by the New Zealand Health Research Council Programme (grant 16–604), and the New Zealand Ministry of Business, Innovation and Employment (MBIE).

R.E.W., E.S., G.D.S., R.B. and K.T. work in a unit that receives funding from the University of Bristol and the UK Medical Research Council (MC_UU_00011/1 and MC_UU_00011/3). This research was funded in part by the Wellcome Trust (204895/Z/16/Z). For the purpose of Open Access, the author has applied a CC BY public copyright licence to any Author Accepted Manuscript version arising from this submission. R.E.W. and A.H. were supported by grants from the South-Eastern Norway Regional Health Authority (2020024 and 2020022). J.A.B. is an MRC Skills Development Fellow. The E-Risk Study is funded by the Medical Research Council (UKMRC grant G1002190). Additional support was provided by National Institute of Child Health and Human Development (grant HD077482) and by the Jacobs Foundation. L.A. is the Mental Health Leadership Fellow for the UK Economic and Social Research Council. T.C. received funding from the European Union’s Horizon 2020 research and innovation programme under grant agreement N: 733206, LIFE-CYCLE project. T.C.E. is part-funded by the National Institute for Health Research (NIHR) Biomedical Research Centre at South London and Maudsley NHS Foundation Trust and King’s College London. The views expressed are those of the author(s) and not necessarily those of the NHS, the NIHR or the Department of Health. K.R. is supported by the Sir Henry Wellcome Postdoctoral Fellowship.

## Supplementary Material

dyac049_Supplementary_Data
