# Supplementary material for: Decline in attention-deficit hyperactivity disorder traits over the life course in the general population: trajectories across five population birth cohorts spanning ages 3 to 45 years
Source: Int J Epidemiol. 2022 Apr 11;51(3):919–30. doi: 10.1093/ije/dyac049 (PMC9189965; doi:10.1093/ije/dyac049)
Supplement: dyac049_Supplementary_Data [file dyac049_supplementary_data.zip › ije-2021-08-1228-File010.docx]

Supplementary Materials

Table of Contents

[Supplementary Tables 3](#_Toc88143120)

[Table S1. Comparison of covariates across cohorts 3](#_Toc88143121)

[Table S2. Missing data across each of the five cohorts. 4](#_Toc88143122)

[Table S3. Fit comparison for SDQ symptom models in ALSPAC to determine the most parsimonious best fitting model. 5](#_Toc88143123)

[Table S4. The difference between predicted and observed values of SDQ for the best fitting model in ALSPAC (parent ratings only) 6](#_Toc88143124)

[Table S5. The difference between predicted and observed values of SDQ for the best fitting model in ALSPAC (all raters) 7](#_Toc88143125)

[Table S6. The difference between predicted and observed values of SDQ for the best fitting model in TEDS (all raters) 8](#_Toc88143126)

[Table S7. The difference between predicted and observed values of SDQ for the best fitting model in ALSPAC, TEDS and Pelotas combined 9](#_Toc88143127)

[Table S8. Comparison of the observed mean and SD SDQ scores across ALSPAC, Pelotas and TEDS 10](#_Toc88143128)

[Table S9. The iterative removal of covariates from the model of SDQ 11](#_Toc88143129)

[Table S10. The difference between predicted and observed values of SDQ for the final model 12](#_Toc88143130)

[Table S11. Coefficients for the best fitting model of SDQ scores 13](#_Toc88143131)

[Table S12. The iterative removal of covariates from the model of SDQ for males only 14](#_Toc88143132)

[Table S13. The iterative removal of covariates from the model of SDQ for females only 15](#_Toc88143133)

[Table S14. The iterative removal of covariates from the model of SDQ for the low SEP group 16](#_Toc88143134)

[Table S15. The iterative removal of covariates from the model of SDQ for the high SEP group 17](#_Toc88143135)

[Table S16. Fit comparison for DSM symptom models in TEDS to determine the most parsimonious best fitting model 18](#_Toc88143136)

[Table S17. The difference between predicted and observed values of DSM for the best fitting model in TEDS (parent ratings only) 19](#_Toc88143137)

[Table S18. The difference between predicted and observed values of DSM for the best fitting model in TEDS (all raters) 20](#_Toc88143138)

[Table S19. The difference between predicted and observed values of DSM for the best fitting model in the ALSPAC cohort 21](#_Toc88143139)

[Table S20. The difference between predicted and observed values of DSM for the best fitting model in the E-Risk cohort 22](#_Toc88143140)

[Table S21. The difference between predicted and observed values of DSM for the best fitting model in the Dunedin cohort 23](#_Toc88143141)

[Table S22. The difference between predicted and observed values of DSM for the best fitting model in all cohorts combined 24](#_Toc88143142)

[Table S23. The iterative removal of covariates from the best fitting model of DSM 25](#_Toc88143143)

[Table S24. The iterative removal of covariates from our model of DSM for males only 26](#_Toc88143144)

[Table S25. The iterative removal of covariates from our model of DSM for females only 27](#_Toc88143145)

[Table S26. The iterative removal of covariates from our model of DSM for the low SEP group only 28](#_Toc88143146)

[Table S27. The iterative removal of covariates from our model of DSM for the high SEP group only 29](#_Toc88143147)

[Table S28. The difference between predicted and observed values of DSM for the best fitting model in all cohorts 30](#_Toc88143148)

[Table S29. Coefficients for the best fitting model of DSM scores 31](#_Toc88143149)

[Supplementary Figures 33](#_Toc88143150)

[Figure S1. Cubic spline model for SDQ in the ALSPAC cohort 33](#_Toc88143151)

[Figure S2. Cubic spline model for SDQ in the TEDS cohort 34](#_Toc88143152)

[Figure S3. SDQ Trajectories extrapolated from age 3 to 27 years 35](#_Toc88143153)

[Figure S4. Benchmark Model of DSM differences across cohorts 36](#_Toc88143154)

[Figure S5. Cubic spline model for DSM in the TEDS cohort 37](#_Toc88143155)

[Figure S6. DSM Trajectories extrapolated from age 5 to 45 years 38](#_Toc88143156)

[Figure S7. Average SDQ scores split by sex 39](#_Toc88143157)

[Figure S8. Average DSM percentage scores split by sex 40](#_Toc88143158)

[Figure S9. Average SDQ scores split by socio-economic position 41](#_Toc88143159)

[Figure S10. Average DSM percentage scores split by socio-economic position 42](#_Toc88143160)

[Figure S11. SDQ Model using only individuals who have completed 3+ assessments across the age range 43](#_Toc88143161)

[Figure S12. DSM Model using only individuals who have completed 3+ assessments across the age range 44](#_Toc88143162)

[Figure S13. Exploratory analysis removing TEDS self-ratings at age 21 years 45](#_Toc88143163)

## Supplementary Tables

### Table S1. Comparison of covariates across cohorts

|  | **ALSPAC** | **TEDS** | **E-Risk** | **Dunedin** | **Pelotas** |
| --- | --- | --- | --- | --- | --- |
| Sex (% female) | 49% | 50% | 51% | 48% | 50% |
| Birthweight (kg) | 3.39 (0.6) | 2.47 (0.6) | 2.44 (0.5) | 3.38 (0.5) | 3.16 (0.6) |
| Gestational age (weeks) | 39 (1.9) | 36 (2.6) | 36 (2.8) | 40 (1.6) | 39 (1.5) |
| Mother age at delivery | 28 (5.0) | 31 (4.7) | 28 (5.9) | 26 (5.0) | 26 (6.4) |

### Table S2. Missing data across each of the five cohorts.

|  | **ALSPAC** |  |  | **TEDS** |  |  | **E-Risk** |  | **Dunedin** |  |  | **Pelotas** |  |  |
| --- | --- | --- | --- | --- | --- | --- | --- | --- | --- | --- | --- | --- | --- | --- |
| **Cohort N** | **15,645** |  |  | **25,656** |  |  | **2,232** |  | **1,037** |  |  | **5,313** |  |  |
| % SDQ | 71% |  |  | 75% |  |  | - |  | - |  |  | 86% |  |  |
| % DSM | 65% |  |  | 60% |  |  | 100% |  | 98% |  |  | 42% |  |  |
| **SDQ** | **None** | **1+** | **P** | **None** | **1+** | **P** | **None** | **1+** | **None** | **1+** | **P** | **None** | **1+** | **P** |
| Sex (%female) | 49% | 49% | 0.62 | 47% | 51% | <0.001 | - | - | - | - | - | 48% | 51% | 0.11 |
| Birthweight (kg) | 3.29 | 3.42 | <0.001 | 2.44 | 2.49 | <0.001 | - | - | - | - | - | 3.03 | 3.18 | <0.001 |
| Gestational age (weeks) | 35 | 39 | <0.001 | 36 | 36 | 0.53 | - | - | - | - | - | 38 | 39 | <0.001 |
| Mother age at delivery | 25 | 29 | <0.001 | 30 | 31 | <0.001 | - | - | - | - | - | 26 | 26 | 0.44 |
| SEP | 9.18 | 9.75 | <0.001 | -0.28 | 0.12 | <0.001 | - | - | - | - | - | 3 | 3.29 |  |
| **DSM** | **None** | **1+** | **P** | **None** | **1+** | **P** | **None** | **1+** | **None** | **1+** | **P** | **None** | **1+** | **P** |
| Sex (%female) | 46% | 50% | <0.001 | 48% | 51% | <0.001 | - | 51% | 41% | 49% | 0.72 | 43% | 53% | <0.001 |
| Birthweight (kg) | 3.32 | 3.43 | <0.001 | 2.44 | 2.5 | <0.001 | - | 2.44 | 3.44 | 3.37 | 0.58 | 3.1 | 3.18 | <0.001 |
| Gestational age (weeks) | 36 | 39 | <0.001 | 36 | 36 | <0.001 | - | 36 | 41 | 40 | 0.03 | 38 | 39 | <0.001 |
| Mother age at delivery | 25 | 29 | <0.001 | 30 | 31 | <0.001 | - | 28 | 27 | 26 | 0.21 | 26 | 26 | 0.85 |
| SEP | 9.21 | 9.79 | <0.001 | -0.22 | 0.18 | <0.001 | - | 2 | 3.71 | 3.75 | 0.88 | 3.27 | 3.41 | <0.001 |

**Note.** Comparison of the covariates for those with at least one ADHD measure compared with those that have no measures of ADHD. P-values represent the difference between groups, calculated used chi-square for categorical traits and t-test for continuous traits. SDQ = strengths and difficulties questionnaire; DSM = Diagnostic and statistical manual; SEP = socio-economic position.

### Table S3. Fit comparison for SDQ symptom models in ALSPAC to determine the most parsimonious best fitting model.

| **Model** | **AIC** | **AIC Difference** |
| --- | --- | --- |
| Null Model | 173,369 | - |
| Random Intercept | 169,366 | -4004 |
| Random Slope | 168,438 | -927 |
| Cubic Spline Model | 165,773 | -2666 |
| knots 6 and 15 | 165,987 | 215 |
| knots 6 and 16 | 165,993 | 221 |
| knots 6 and 17 | 166,022 | 249 |
| knots 7 and 15 | 165,923 | 150 |
| knots 7 and 16 | 165,926 | 153 |
| knots 7 and 17 | 165,952 | 180 |
| knots 8 and 15 | 165,903 | 130 |
| **knots 8 and 16** | **165,900** | **128** |
| knots 8 and 17 | 165,922 | 149 |

**Note.** Selected model in bold. Difference in AIC is stepwise, always comparing to the previously best fitting model. The linear spline model was chosen over the cubic spline model on the basis of parsimony.

### Table S4. The difference between predicted and observed values of SDQ for the best fitting model in ALSPAC (parent ratings only)

|  | **N** | **Observed Mean** | **Observed SD** | **Predicted Mean** | **Predicted SD** | **Mean Difference** | **SD Difference** | **Minimum Difference** | **Maximum Difference** |
| --- | --- | --- | --- | --- | --- | --- | --- | --- | --- |
| Overall | 42,542 | 3.05 | 1.6 | 3.12 | 0.05 | 0.08 | 2.25 | -8.58 | 3.97 |
| 3-5 | 6,818 | 3.93 | 2.32 | 3.95 | 0.01 | 0.01 | 2.32 | -6.09 | 3.97 |
| 5-7 | 6,072 | 3.34 | 2.35 | 3.41 | 0.01 | 0.06 | 2.35 | -6.62 | 3.42 |
| 7-9 | 6,253 | 3.3 | 2.44 | 3.2 | 0.04 | -0.1 | 2.44 | -6.85 | 3.37 |
| 9-11 | 6,057 | 2.91 | 2.25 | 3.1 | 0.01 | 0.19 | 2.25 | -6.92 | 3.14 |
| 11-13 | 5,533 | 2.74 | 2.22 | 2.97 | 0.01 | 0.23 | 2.22 | -7.06 | 2.98 |
| 13-15 | 5,320 | 2.87 | 2.18 | 2.88 | 0.01 | 0.01 | 2.18 | -7.2 | 2.89 |
| 15-17 | 2,942 | 2.53 | 2.11 | 2.63 | 0.02 | 0.1 | 2.11 | -7.4 | 2.74 |
| 17-19 | 1,450 | 2.54 | 2.08 | 2.56 | 0.02 | 0.02 | 2.08 | -7.48 | 2.59 |
| 19-21 | 0 | - | - | - | - | - | - | - | - |
| 21-23 | 0 | - | - | - | - | - | - | - | - |
| 23-25 | 100 | 1.54 | 1.87 | 1.67 | 0.01 | 0.13 | 1.87 | -8.33 | 1.67 |
| 25-27 | 1,997 | 1.3 | 1.6 | 1.56 | 0.05 | 0.26 | 1.61 | -8.58 | 1.65 |

**Note.** The best fitting model has linear splines with knots at 8 and 16 years. SD = standard deviation.

### Table S5. The difference between predicted and observed values of SDQ for the best fitting model in ALSPAC (all raters)

|  | **N** | **Observed Mean** | **Observed SD** | **Predicted Mean** | **Predicted SD** | **Mean Difference** | **SD Difference** | **Minimum Difference** | **Maximum Difference** |
| --- | --- | --- | --- | --- | --- | --- | --- | --- | --- |
| Overall | 54,937 | 2.93 | 2.1 | 3.06 | 0.84 | 0.13 | 2.31 | -8.54 | 3.99 |
| 3-5 | 6,818 | 3.93 | 2.32 | 3.95 | 0.01 | 0.01 | 2.32 | -6.09 | 3.97 |
| 5-7 | 6,072 | 3.34 | 2.35 | 3.41 | 0.01 | 0.06 | 2.35 | -6.62 | 3.42 |
| 7-9 | 6,253 | 3.3 | 2.44 | 3.2 | 0.04 | -0.1 | 2.44 | -6.85 | 3.37 |
| 9-11 | 6,057 | 2.91 | 2.25 | 3.1 | 0.01 | 0.19 | 2.25 | -6.92 | 3.14 |
| 11-13 | 5,533 | 2.74 | 2.22 | 2.97 | 0.01 | 0.23 | 2.22 | -7.06 | 2.98 |
| 13-15 | 5,320 | 2.87 | 2.18 | 2.88 | 0.01 | 0.01 | 2.18 | -7.2 | 2.89 |
| 15-17 | 2,942 | 2.53 | 2.11 | 2.63 | 0.02 | 0.1 | 2.11 | -7.4 | 2.74 |
| 17-19 | 1,450 | 2.54 | 2.08 | 2.56 | 0.02 | 0.02 | 2.08 | -7.48 | 2.59 |
| 19-21 | 0 | - | - | - | - | - | - | - | - |
| 21-23 | 0 | - | - | - | - | - | - | - | - |
| 23-25 | 100 | 1.54 | 1.87 | 1.67 | 0.01 | 0.13 | 1.87 | -8.33 | 1.67 |
| 25-27 | 1,997 | 1.3 | 1.6 | 1.56 | 0.05 | 0.26 | 1.61 | -8.58 | 1.65 |

**Note.** The best fitting model has linear splines with knots at 8 and 16 years. SD = standard deviation.

### Table S6. The difference between predicted and observed values of SDQ for the best fitting model in TEDS (all raters)

|  | **N** | **Observed Mean** | **Observed SD** | **Predicted Mean** | **Predicted SD** | **Mean Difference** | **SD Difference** | **Minimum Difference** | **Maximum Difference** |
| --- | --- | --- | --- | --- | --- | --- | --- | --- | --- |
| Overall | 53,845 | 3.06 | 2.17 | 3.16 | 0.93 | 0.10 | 2.31 | -7.81 | 4.19 |
| 3-5 | 6,208 | 3.90 | 2.29 | 4.00 | 0.02 | 0.10 | 2.29 | -6.12 | 4.03 |
| 5-7 | 3,898 | 3.47 | 2.61 | 3.27 | 0.24 | -0.20 | 2.59 | -7.10 | 3.53 |
| 7-9 | 10,431 | 3.15 | 2.51 | 3.18 | 0.45 | 0.03 | 2.48 | -7.40 | 4.19 |
| 9-11 | 8,398 | 3.05 | 2.39 | 3.25 | 0.64 | 0.21 | 2.33 | -7.60 | 4.15 |
| 11-13 | 8,688 | 2.78 | 2.35 | 3.02 | 0.65 | 0.24 | 2.29 | -7.77 | 3.95 |
| 13-15 | 89 | 3.25 | 2.44 | 3.04 | 0.54 | -0.21 | 2.38 | -5.49 | 3.56 |
| 15-17 | 6,596 | 2.89 | 2.23 | 2.94 | 0.52 | 0.05 | 2.14 | -7.61 | 3.55 |
| 17-19 | 1,455 | 2.81 | 2.20 | 2.89 | 0.52 | 0.09 | 2.11 | -7.62 | 3.42 |
| 19-21 | 850 | 2.69 | 2.20 | 2.76 | 0.52 | 0.07 | 2.11 | -7.72 | 3.32 |
| 21-23 | 5,219 | 2.60 | 2.19 | 2.73 | 0.52 | 0.13 | 2.08 | -7.78 | 3.31 |
| 23-25 | 2,008 | 2.43 | 2.17 | 2.66 | 0.52 | 0.24 | 2.06 | -7.81 | 3.25 |
| 25-27 | 5 | 2.80 | 2.17 | 3.59 | 0.93 | 0.79 | 1.49 | -1.78 | 2.13 |

**Note.** The best fitting model has linear splines with knots at 8 and 16 years. SD = standard deviation.

### Table S7. The difference between predicted and observed values of SDQ for the best fitting model in ALSPAC, TEDS and Pelotas combined

|  | **N** | **Observed Mean** | **Observed SD** | **Predicted Mean** | **Predicted SD** | **Mean Difference** | **SD Difference** | **Minimum Difference** | **Maximum Difference** |
| --- | --- | --- | --- | --- | --- | --- | --- | --- | --- |
| Overall | 121,452 | 3.08 | 2.10 | 3.18 | 0.32 | 0.10 | 2.38 | -7.86 | 4.42 |
| 3-5 | 13,026 | 3.92 | 2.31 | 3.97 | 0.03 | 0.06 | 2.31 | -6.15 | 4.01 |
| 5-7 | 9,970 | 3.39 | 2.45 | 3.36 | 0.23 | -0.03 | 2.45 | -7.24 | 3.55 |
| 7-9 | 20,936 | 3.06 | 2.53 | 3.03 | 0.40 | -0.03 | 2.50 | -7.52 | 3.90 |
| 9-11 | 17,490 | 3.03 | 2.42 | 3.20 | 0.53 | 0.17 | 2.38 | -7.64 | 4.42 |
| 11-13 | 24,731 | 2.96 | 2.54 | 3.19 | 0.67 | 0.24 | 2.48 | -7.71 | 4.35 |
| 13-15 | 8,831 | 3.24 | 2.60 | 3.13 | 0.28 | -0.11 | 2.57 | -7.17 | 3.51 |
| 15-17 | 10,256 | 2.84 | 2.28 | 3.01 | 0.34 | 0.16 | 2.22 | -7.32 | 3.49 |
| 17-19 | 2,905 | 2.68 | 2.14 | 2.85 | 0.30 | 0.17 | 2.10 | -7.35 | 3.38 |
| 19-21 | 850 | 2.69 | 2.20 | 2.83 | 0.33 | 0.13 | 2.13 | -7.48 | 3.18 |
| 21-23 | 5,219 | 2.60 | 2.19 | 2.76 | 0.33 | 0.16 | 2.11 | -7.58 | 3.16 |
| 23-25 | 2,251 | 2.43 | 2.18 | 2.67 | 0.33 | 0.23 | 2.09 | -7.74 | 3.05 |
| 25-27 | 4,987 | 2.37 | 2.10 | 2.60 | 0.32 | 0.22 | 1.99 | -7.86 | 2.90 |

**Note.** The best fitting model has linear splines with knots at 8 and 16 years with covariate fixed effects for rater and cohort. SD = standard deviation.

### Table S8. Comparison of the observed mean and SD SDQ scores across ALSPAC, Pelotas and TEDS

|  | **ALSPAC** |  |  | **TEDS** |  |  | **Pelotas** |  |  |
| --- | --- | --- | --- | --- | --- | --- | --- | --- | --- |
|  | **N** | **Mean** | **SD** | **N** | **Mean** | **SD** | **N** | **Mean** | **SD** |
| 3-5 | 6,818 | 3.93 | 2.32 | 6,208 | 3.90 | 2.29 | 0 | - | - |
| 5-7 | 6,072 | 3.34 | 2.35 | 3,898 | 3.47 | 2.61 | 0 | - | - |
| 7-9 | 10,505 | 2.98 | 2.54 | 10,431 | 3.15 | 2.51 | 0 | - | - |
| 9-11 | 7,358 | 2.81 | 2.33 | 8,398 | 3.05 | 2.39 | 1,734 | 3.89 | 2.76 |
| 11-13 | 9,247 | 2.49 | 2.38 | 8,688 | 2.78 | 2.35 | 6,796 | 3.82 | 2.76 |
| 13-15 | 5,320 | 2.87 | 2.18 | 89 | 3.25 | 2.44 | 3,422 | 3.82 | 3.06 |
| 15-17 | 2,942 | 2.53 | 2.11 | 6,596 | 2.89 | 2.23 | 718 | 3.69 | 3.02 |
| 17-19 | 1,450 | 2.54 | 2.08 | 1,455 | 2.81 | 2.20 | 0 | - | - |
| 19-21 | 0 | - | - | 850 | 2.69 | 2.20 | 0 | - | - |
| 21-23 | 0 | - | - | 5,219 | 2.60 | 2.19 | 0 | - | - |
| 23-25 | 243 | 2.49 | 2.24 | 2,008 | 2.43 | 2.17 | 0 | - | - |
| 25-27 | 4,982 | 2.37 | 2.1 | 5 | 2.80 | 2.17 | 0 | - | - |

**Note.** As relatedness could not be accounted for here, data for this table are first born child for ALSPAC only and one twin selected at random for TEDS.

### Table S9. The iterative removal of covariates from the model of SDQ

| **Model** | **AIC** | **AIC Difference** | **Decision** |
| --- | --- | --- | --- |
| All covariates | 748,176 | - | - |
| Birthweight removed | 748,237 | 61 | Keep |
| Gestational age removed | 748,178 | 2 | Drop |
| Mother's age at delivery removed | 748,333 | 155 | Keep |
| SEP removed | 748,642 | 463 | Keep |
| Sex removed | 749,827 | 1648 | Keep |
| Rater removed | 759,790 | 11612 | Keep |
| Cohort*birthweight interaction removed | 748,174 | -4 | Drop |
| **Cohort*birthweight interaction removed** | **748,173** | **-1** | **Drop** |
| Cohort*age at delivery interaction removed | 748,250 | 77 | Keep |
| Cohort*SEP interaction removed | 748,214 | 40 | Keep |
| Cohort*rater interaction removed | 751,032 | 2859 | Keep |
| Cohort*slope interaction removed | 748,401 | 228 | Keep |

**Note.** We iteratively removed each covariate based upon the order in which they least changed the coefficient. If the AIC increased by more than 4 than the last best fitting model then we kept them in the model. The best fitting/most parsimonious model is highlighted in bold. SEP = socio-economic position.

### Table S10. The difference between predicted and observed values of SDQ for the final model

|  | **N** | **Observed Mean** | **Observed SD** | **Predicted Mean** | **Predicted SD** | **Mean Difference** | **SD Difference** | **Minimum Difference** | **Maximum Difference** |
| --- | --- | --- | --- | --- | --- | --- | --- | --- | --- |
| Overall | 175,833 | 3.07 | 2.32 | 3.15 | 0.50 | 0.08 | 2.30 | -9.07 | 5.66 |
| 3-5 | 28,821 | 3.25 | 2.56 | 3.20 | 0.58 | -0.05 | 2.46 | -8.13 | 4.49 |
| 5-7 | 31,210 | 3.07 | 2.42 | 3.16 | 0.72 | 0.09 | 2.31 | -8.36 | 5.20 |
| 7-9 | 39,754 | 2.96 | 2.50 | 3.14 | 0.85 | 0.18 | 2.34 | -8.51 | 5.66 |
| 9-11 | 10,437 | 2.81 | 2.32 | 2.86 | 0.67 | 0.05 | 2.22 | -8.28 | 4.91 |
| 11-13 | 10,171 | 3.31 | 2.64 | 3.30 | 0.77 | -0.01 | 2.54 | -8.12 | 5.08 |
| 13-15 | 14,688 | 2.72 | 2.19 | 2.79 | 0.67 | 0.08 | 2.10 | -8.43 | 4.61 |
| 15-17 | 34 | 3.53 | 2.75 | 2.72 | 0.73 | -0.81 | 2.54 | -8.06 | 4.15 |
| 17-19 | 6,786 | 2.64 | 2.18 | 2.66 | 0.69 | 0.02 | 2.10 | -8.33 | 4.26 |
| 19-21 | 8,906 | 2.47 | 2.17 | 2.64 | 0.69 | 0.17 | 2.06 | -8.68 | 4.33 |
| 21-23 | 3,813 | 2.38 | 2.13 | 2.56 | 0.94 | 0.18 | 1.94 | -9.07 | 4.54 |
| 23-25 | 1,878 | 2.37 | 2.09 | 2.51 | 0.96 | 0.13 | 1.96 | -8.51 | 4.46 |
| 25-27 | 19,335 | 3.94 | 2.32 | 4.01 | 0.50 | 0.07 | 2.27 | -6.89 | 5.20 |

**Note.** Fit is for the best fitting model of SDQ scores (linear splines with knots at 8 and 16 years) using data from all 3 cohorts combined (TEDS, ALSPAC and Pelotas) with covariates included. SD = standard deviation.

### Table S11. Coefficients for the best fitting model of SDQ scores

|  |  | Centred at the mean for the total sample | Centred at the mean for each cohort |
| --- | --- | --- | --- |
|  |  | **Coefficient (95% CI)** | **Coefficient (95% CI)** |
| **Fixed Effects** | Intercept | 4.463 (4.399, 4.528) | 5.026 (4.881, 5.170) |
|  | Spline 1 (Age 3-8 years) | -0.157 (-0.170, -0.144) | -0.157 (-0.170, -0.144) |
|  | Spline 2 (Age 8-16 years) | -0.067 (-0.075, -0.059) | -0.067 (-0.075, -0.059) |
|  | Spline 3 (Age 16+ years) | -0.106 (-0.116, -0.097) | -0.106 (-0.116, -0.097) |
|  | Rater (self) | 1.712 (1.616, 1.808) | 1.712 (1.616, 1.808) |
|  | Rater (teacher) | -0.799 (-0.844, -0.755) | -0.799 (-0.844, -0.755) |
|  | Cohort (TEDS) | 0.092 (0.006, 0.178) | 0.015 (-0.069, 0.100) |
|  | Cohort (Pelotas) | 1.341 (1.216, 1.466) | 1.373 (1.252, 1.493) |
|  | Sex (female) | -0.818 (-0.856, -0.780) | -0.818 (-0.856, -0.780) |
|  | Birthweight | -0.160 (-0.198, -0.122) | -0.0002 (-0.0002, -0.0001) |
|  | Mother’s age at delivery | -0.042 (-0.049, -0.034) | -0.042 (-0.049, -0.034) |
|  | SEP | -0.145 (-0.179, -0.111) | -0.145 (-0.179, -0.111) |
|  | Rater (self) * Cohort (TEDS) | -0.691 (-0.791, -0.591) | -0.691 (-0.791, -0.591) |
|  | Rater (teacher) * Cohort (TEDS) | 0.269 (0.215, 0.323) | 0.269 (0.215, 0.323) |
|  | Rater (self) * Cohort (Pelotas) | -2.691 (-2.812, -2.570) | -2.691 (-2.812, -2.570) |
|  | Cohort (TEDS) * Mother’s age at delivery | 0.048 (0.037, 0.059) | 0.048 (0.037, 0.059) |
|  | Cohort (Pelotas) * Mother’s age at delivery | 0.013 (0.001, 0.024) | 0.013 (0.001, 0.024) |
|  | Cohort (TEDS) * SEP | -0.157 (-0.205, -0.110) | -0.157 (-0.205, -0.110) |
|  | Cohort (Pelotas) * SEP | -0.130 (-0.195, -0.065) | -0.130 (-0.195, -0.065) |
|  | Spline 1 * Cohort (TEDS) | -0.049 (-0.066, -0.031) | -0.049 (-0.066, -0.031) |
|  | Spline 2 * Cohort (TEDS) | -0.027 (-0.037, -0.017) | -0.027 (-0.037, -0.017) |
|  | Spline 2 * Cohort (Pelotas) | -0.065 (-0.086, -0.044) | -0.065 (-0.086, -0.044) |
|  | Spline 3 * Cohort (TEDS) | 0.076 (0.065, 0.087) | 0.076 (0.065, 0.087) |
| **Random Effects (Level 3)** | Variance Intercept | 0.813 (0.758, 0.868) | 0.813 (0.758, 0.868) |
| **Random Effects (Level 2)** | Variance Intercept | 1.864 (1.719, 2.009) | 1.864 (1.719, 2.009) |
|  | Covariance Intercept and Spline 1 | -0.093 (-0.121, -0.065) | -0.093 (-0.121, -0.065) |
|  | Variance Spline 1 | 0.054 (0.046, 0.061) | 0.054 (0.046, 0.061) |
|  | Covariance Intercept and Spline 2 | -0.121 (-0.134, -0.108) | -0.121 (-0.134, -0.108) |
|  | Covariance Spline 1 and Spline 2 | -0.0005 (-0.004, 0.003) | -0.0005 (-0.004, 0.003) |
|  | Variance Spline 2 | 0.020 (0.018, 0.022) | 0.020 (0.018, 0.022) |
|  | Covariance Intercept and Spline 3 | -0.030 (-0.043, -0.017) | -0.030 (-0.043, -0.017) |
|  | Covariance Spline 1 and Spline 3 | -0.014 (-0.017, -0.011) | -0.014 (-0.017, -0.011) |
|  | Covariance Spline 2 and Spline 3 | 0.004 (0.003, 0.006) | 0.004 (0.003, 0.006) |
|  | Variance Spline 3 | 0.0005 (-0.001, 0.002) | 0.0005 (-0.001, 0.002) |
| **Random Effects (Level 1)** | Variance Intercept | 2.995 (2.970, 3.021) | 2.995 (2.970, 3.021) |

**Note.** The primary model reported in the main text has all continuous covariates centred to the mean for the total sample (birthweight = 3kg, gestational age = 38 weeks, mother’s age at delivery = 30 years). This is compared with a model where each cohort had covariates centred to the mean for that cohort (covariate means by cohort available in Supplementary Table S1). SEP = socio-economic position.

### Table S12. The iterative removal of covariates from the model of SDQ for males only

| **Model** | **AIC** | **AIC Difference** | **Decision** |
| --- | --- | --- | --- |
| All covariates | 366,914 | - | - |
| Birthweight removed | 366,933 | 19 | Keep |
| Gestational age removed | 366,910 | -4 | Drop |
| Mother's age at delivery removed | 366,982 | 72 | Keep |
| SEP removed | 367,132 | 221 | Keep |
| Rater removed | 370,140 | 3229 | Keep |
| **Cohort*birthweight removed** | **366,906** | **-4** | **Drop** |
| Cohort*age at delivery removed | 366,938 | 32 | Keep |
| Cohort*SEP removed | 366,926 | 20 | Keep |
| Cohort*rater removed | 368,325 | 1418 | Keep |
| Cohort*slope removed | 367,042 | 135 | Keep |

**Note.** We iteratively removed each covariate based upon the order in which they least changed the coefficient. If the AIC increased by more than 4 then we kept them in the model. SEP = socio-economic position.

### Table S13. The iterative removal of covariates from the model of SDQ for females only

| **Model** | **AIC** | **AIC Difference** | **Decision** |
| --- | --- | --- | --- |
| All covariates | 378,485 | - | - |
| Birthweight removed | 378,527 | 42 | Keep |
| Gestational age removed | 378,489 | 4 | Drop |
| Mother's age at delivery removed | 378,564 | 75 | Keep |
| SEP removed | 378,760 | 271 | Keep |
| Rater removed | 388,181 | 9692 | Keep |
| **Cohort*birthweight interaction removed** | **378,486** | **-3** | **Drop** |
| Cohort*age at delivery interaction removed | 378,526 | 41 | Keep |
| Cohort*SEP interaction removed | 378,514 | 28 | Keep |
| Cohort*rater interaction removed | 379,965 | 1480 | Keep |
| Cohort*slope interaction removed | 378,579 | 93 | Keep |

**Note.** We iteratively removed each covariate based upon the order in which they least changed the coefficient. If the AIC increased by more than 4 then we kept them in the model. SEP = socio-economic position.

### Table S14. The iterative removal of covariates from the model of SDQ for the low SEP group

| **Model** | **AIC** | **AIC Difference** | **Decision** |
| --- | --- | --- | --- |
| All covariates | 271,113 | - | - |
| Birthweight removed | 271,130 | 18 | Keep |
| Gestational age removed | 271,113 | 1 | Drop |
| Mother's age at delivery removed | 271,154 | 41 | Keep |
| Sex removed | 271,758 | 645 | Keep |
| Rater removed | 274,607 | 3494 | Keep |
| Cohort*birthweight removed | 271,111 | -2 | Drop |
| **Cohort*age at delivery removed** | **271,114** | **3** | **Drop** |
| Cohort*sex removed | 271,129 | 15 | Keep |
| Cohort*rater removed | 272,260 | 1146 | Keep |
| Cohort*slope removed | 271,154 | 40 | Keep |

**Note.** We iteratively removed each covariate based upon the order in which they least changed the coefficient. If the AIC increased by more than 4 then we kept them in the model. SEP = socio-economic position.

### Table S15. The iterative removal of covariates from the model of SDQ for the high SEP group

| **Model** | **AIC** | **AIC Difference** | **Decision** |
| --- | --- | --- | --- |
| All covariates | 343,252 | - | - |
| Birthweight removed | 343,271 | 19 | Keep |
| Gestational age removed | 343,249 | -3 | Drop |
| Mother's age at delivery removed | 343,289 | 39 | Keep |
| Sex removed | 343,921 | 672 | Keep |
| Rater removed | 349,424 | 6174 | Keep |
| **Cohort*birthweight interaction removed** | **343,247** | **-2** | **Drop** |
| Cohort*age at delivery interaction removed | 343,263 | 15 | Keep |
| Cohort*Sex interaction removed | 343,261 | 14 | Keep |
| Cohort*rater interaction removed | 344,796 | 1549 | Keep |
| Cohort*slope interaction removed | 343,311 | 64 | Keep |

**Note.** We iteratively removed each covariate based upon the order in which they least changed the coefficient. If the AIC increased by more than 4 then we kept them in the model. SEP = socio-economic position.

### Table S16. Fit comparison for DSM symptom models in TEDS to determine the most parsimonious best fitting model

| **Model** | **AIC** | **difference** |
| --- | --- | --- |
| Null Model | 168,283 | - |
| Random Intercept | 166,566 | -1717 |
| Random Slope | 165,584 | -982 |
| Random slope with sex fixed effects | 165,361 | -223 |
| Cubic Spline Model | 164,680 | -681 |
| **knots 14 17 and 21** | **164,627** | **-53** |
| knots 14 17 and 22 | 164,632 | -48 |
| knots 14 16 and 21 | 164,640 | -40 |
| knots 14 and 20 | 164,812 | 132 |
| knots 14 and 21 | 164,874 | 194 |
| knots 16 and 20 | 164,874 | 194 |

**Note.** Selected model in bold. Difference in AIC is stepwise, always comparing to the previously best fitting model. We tested 3 knot points at 14/15, 16/17/18 and 21/22/23. The top 3 models are presented here. We tested 2 knot points at 14/15/15/17 and 20/21/22/23. The top 3 models are presented here.

### Table S17. The difference between predicted and observed values of DSM for the best fitting model in TEDS (parent ratings only)

|  | **N** | **Observed Mean** | **Observed SD** | **Predicted Mean** | **Predicted SD** | **Mean Difference** | **SD Difference** | **Minimum Difference** | **Maximum Difference** |
| --- | --- | --- | --- | --- | --- | --- | --- | --- | --- |
| Overall | 21,099 | 15.92 | 9.95 | 16.70 | 2.28 | 0.78 | 14.79 | -85.78 | 23.80 |
| 6-8 | 3,234 | 20.65 | 17.07 | 21.15 | 2.27 | 0.50 | 16.76 | -73.80 | 23.80 |
| 8-10 | 2,294 | 19.88 | 17.27 | 20.42 | 2.27 | 0.54 | 16.92 | -81.66 | 23.10 |
| 10-12 | 3,826 | 17.94 | 15.47 | 18.70 | 2.29 | 0.76 | 15.09 | -79.21 | 21.82 |
| 12-14 | 1,975 | 17.11 | 15.88 | 17.50 | 2.32 | 0.39 | 15.59 | -81.42 | 20.58 |
| 14-16 | 2,848 | 14.35 | 14.46 | 15.36 | 2.46 | 1.01 | 14.20 | -78.43 | 19.27 |
| 16-18 | 2,598 | 11.30 | 12.86 | 12.70 | 2.29 | 1.41 | 12.71 | -80.47 | 16.21 |
| 18-20 | 7 | 28.31 | 13.30 | 14.08 | 2.09 | -14.23 | 13.24 | -33.28 | 2.30 |
| 20-22 | 1,785 | 12.71 | 13.32 | 13.44 | 2.27 | 0.73 | 13.23 | -79.49 | 16.28 |
| 22-24 | 2,396 | 11.11 | 12.46 | 12.01 | 2.32 | 0.91 | 12.49 | -85.78 | 15.33 |
| 24-26 | 136 | 9.62 | 9.95 | 10.43 | 2.28 | 0.80 | 10.35 | -39.33 | 13.25 |

**Note.** The best fitting model has linear splines with knots at 14, 17 and 21 years. SD = standard deviation.

### Table S18. The difference between predicted and observed values of DSM for the best fitting model in TEDS (all raters)

|  | **N** | **Observed Mean** | **Observed SD** | **Predicted Mean** | **Predicted SD** | **Mean Difference** | **SD Difference** | **Minimum Difference** | **Maximum Difference** |
| --- | --- | --- | --- | --- | --- | --- | --- | --- | --- |
| Overall | 27,913 | 17.60 | 14.66 | 18.43 | 5.25 | 0.83 | 14.92 | -86.37 | 30.02 |
| 6-8 | 3,234 | 20.65 | 17.07 | 21.27 | 2.11 | 0.63 | 16.77 | -73.53 | 23.82 |
| 8-10 | 2,294 | 19.88 | 17.27 | 20.45 | 2.12 | 0.57 | 16.94 | -81.45 | 23.02 |
| 10-12 | 3,826 | 17.94 | 15.47 | 18.49 | 2.14 | 0.55 | 15.10 | -79.65 | 21.54 |
| 12-14 | 3,622 | 19.80 | 16.57 | 20.64 | 5.77 | 0.84 | 15.76 | -81.74 | 30.02 |
| 14-16 | 4,552 | 17.43 | 15.65 | 18.37 | 5.85 | 0.93 | 14.69 | -86.37 | 29.13 |
| 16-18 | 2,598 | 11.30 | 12.86 | 12.76 | 2.12 | 1.47 | 12.71 | -80.21 | 15.95 |
| 18-20 | 7 | 28.31 | 13.30 | 14.04 | 1.94 | -14.26 | 13.24 | -33.11 | 2.18 |
| 20-22 | 2,594 | 16.10 | 14.63 | 16.71 | 5.18 | 0.62 | 13.70 | -79.21 | 26.60 |
| 22-24 | 4,647 | 16.77 | 14.93 | 17.73 | 5.71 | 0.96 | 13.75 | -85.25 | 26.26 |
| 24-26 | 539 | 18.51 | 14.66 | 19.79 | 5.25 | 1.28 | 13.98 | -79.33 | 25.04 |

**Note.** The best fitting model has linear splines with knots at 14, 17 and 21 years. SD = standard deviation.

### Table S19. The difference between predicted and observed values of DSM for the best fitting model in the ALSPAC cohort

|  | **N** | **Observed Mean** | **Observed SD** | **Predicted Mean** | **Predicted SD** | **Mean Difference** | **SD Difference** | **Minimum Difference** | **Maximum Difference** |
| --- | --- | --- | --- | --- | --- | --- | --- | --- | --- |
| Overall | 30,524 | 10.86 | - | 11.49 | - | 0.63 | 16.12 | -94.25 | 16.87 |
| 6-8 | 6,606 | 13.48 | 18.75 | 13.83 | 1.82 | 0.35 | 18.54 | -88.12 | 15.6 |
| 8-10 | 216 | 15.12 | 21.26 | 13.48 | 1.83 | -1.64 | 20.92 | -84.59 | 15.46 |
| 10-12 | 6,278 | 12.48 | 18.05 | 12.79 | 1.82 | 0.31 | 17.85 | -89.08 | 14.64 |
| 12-14 | 4,740 | 10.93 | 16.87 | 11.76 | 1.82 | 0.83 | 16.68 | -86.47 | 14.15 |
| 14-16 | 4,745 | 9.51 | 15.38 | 10.1 | 2 | 0.59 | 15.19 | -88.98 | 13.5 |
| 16-18 | 168 | 7.29 | 12.79 | 8.17 | 1.82 | 0.89 | 12.69 | -67.97 | 10.67 |
| 18-20 | 0 | - | - | - | - | - | - | - | - |
| 20-22 | 0 | - | - | - | - | - | - | - | - |
| 22-24 | 0 | - | - | - | - | - | - | - | - |
| 24-26 | 4,978 | 7.84 | 12.58 | 9.15 | 4.71 | 1.31 | 11.75 | -87.45 | 16.87 |
| 26-28 | 0 | - | - | - | - | - | - | - | - |

**Note.** The best fitting model has linear splines with knots at 14, 17 and 21 years. Both parent and self-ratings from ALSPAC were included. SD = standard deviation.

### Table S20. The difference between predicted and observed values of DSM for the best fitting model in the E-Risk cohort

|  | **N** | **Observed Mean** | **Observed SD** | **Predicted Mean** | **Predicted SD** | **Mean Difference** | **SD Difference** | **Minimum Difference** | **Maximum Difference** |
| --- | --- | --- | --- | --- | --- | --- | --- | --- | --- |
| Overall | 9,332 | 22.18 | 23.60 | 22.29 | 9.27 | 0.11 | 20.19 | -89.40 | 35.72 |
| 5 | 1,995 | 24.80 | 22.46 | 24.45 | 8.88 | -0.36 | 20.25 | -87.26 | 35.72 |
| 7 | 1,949 | 22.48 | 21.78 | 22.97 | 8.87 | 0.49 | 19.79 | -79.56 | 34.21 |
| 10 | 1,867 | 20.48 | 21.24 | 20.83 | 8.88 | 0.35 | 19.05 | -77.15 | 31.94 |
| 12 | 1,793 | 20.00 | 21.77 | 19.75 | 8.83 | -0.25 | 20.04 | -83.34 | 30.43 |
| 18 | 1,728 | 22.91 | 23.60 | 23.27 | 9.27 | 0.35 | 21.86 | -89.40 | 34.51 |

**Note.** The best fitting model has linear splines with knots at 14, 17 and 21 years. Both parent, teacher and self-ratings from E-Risk were included. SD = standard deviation.

### Table S21. The difference between predicted and observed values of DSM for the best fitting model in the Dunedin cohort

|  | **N** | **Observed Mean** | **Observed SD** | **Predicted Mean** | **Predicted SD** | **Mean Difference** | **SD Difference** | **Minimum Difference** | **Maximum Difference** |
| --- | --- | --- | --- | --- | --- | --- | --- | --- | --- |
| Overall | 9,327 | 15.23 | 14.06 | 15.39 | 1.79 | 0.17 | 16.62 | -85.97 | 22.50 |
| 9 | 1,624 | 19.61 | 19.17 | 18.98 | 2.53 | -0.64 | 18.62 | -76.54 | 22.50 |
| 11 | 2,295 | 15.28 | 17.45 | 16.43 | 2.33 | 1.15 | 17.10 | -82.16 | 20.06 |
| 13 | 2,084 | 14.22 | 16.50 | 14.00 | 2.34 | -0.22 | 16.22 | -85.97 | 17.61 |
| 15 | 1,686 | 18.16 | 16.41 | 18.34 | 2.10 | 0.18 | 16.43 | -76.26 | 21.19 |
| 38 | 833 | 9.30 | 14.29 | 9.36 | 1.79 | 0.06 | 14.44 | -77.73 | 11.16 |
| 45 | 805 | 8.83 | 14.06 | 8.93 | 1.79 | 0.10 | 14.22 | -83.72 | 10.73 |

**Note.** The best fitting model has linear splines with knots at 14, 17 and 21 years. Both parent, teacher and self-ratings from E-Risk were included. SD = standard deviation.

### Table S22. The difference between predicted and observed values of DSM for the best fitting model in all cohorts combined

|  | **N** | **Observed Mean** | **Observed SD** | **Predicted Mean** | **Predicted SD** | **Mean Difference** | **SD Difference** | **Minimum Difference** | **Maximum Difference** |
| --- | --- | --- | --- | --- | --- | --- | --- | --- | --- |
| Overall | 80,613 | 15.76 | 14.06 | 16.29 | 1.79 | 0.53 | 16.75 | -94.14 | 30.29 |
| 5-7 | 2,183 | 24.61 | 22.24 | 23.52 | 4.50 | -1.09 | 20.75 | -82.43 | 29.66 |
| 7-9 | 13,839 | 17.26 | 19.02 | 17.99 | 4.20 | 0.74 | 18.21 | -87.38 | 28.65 |
| 9-11 | 11,289 | 15.93 | 18.86 | 15.51 | 4.53 | -0.43 | 18.08 | -88.87 | 27.12 |
| 11-13 | 7,446 | 17.12 | 17.91 | 18.36 | 4.95 | 1.24 | 17.69 | -85.46 | 26.65 |
| 13-15 | 13,590 | 15.20 | 17.15 | 15.51 | 6.07 | 0.31 | 16.37 | -90.93 | 27.48 |
| 15-17 | 9,087 | 12.13 | 15.03 | 12.74 | 4.42 | 0.60 | 14.54 | -89.75 | 23.52 |
| 17-19 | 2,472 | 19.39 | 21.58 | 20.13 | 6.04 | 0.74 | 19.93 | -82.68 | 28.63 |
| 19-21 | 458 | 14.28 | 14.08 | 15.12 | 1.78 | 0.84 | 14.02 | -69.76 | 17.14 |
| 21-23 | 7,830 | 21.14 | 18.38 | 21.86 | 6.06 | 0.72 | 17.02 | -83.39 | 30.29 |
| 23-25 | 3,373 | 17.60 | 16.20 | 18.27 | 5.85 | 0.68 | 14.86 | -80.49 | 29.70 |
| 25-27 | 7,396 | 7.99 | 12.63 | 9.17 | 4.17 | 1.18 | 11.86 | -87.82 | 16.36 |
| 27-29 | 12 | 35.65 | 40.05 | 8.16 | 4.46 | -27.49 | 39.90 | -94.14 | 9.89 |
| 38 | 833 | 9.30 | 14.29 | 12.10 | 1.79 | 2.80 | 14.44 | -75.00 | 13.89 |
| 45 | 805 | 8.83 | 14.06 | 8.43 | 1.79 | -0.40 | 14.22 | -84.22 | 10.23 |

**Note.** The best fitting model has linear splines with knots at 14, 17 and 21 years with covariate fixed effects for sex, rater and cohort. SD = standard deviation.

### Table S23. The iterative removal of covariates from the best fitting model of DSM

| **Model** | **AIC** | **AIC Difference** | **Decision** |
| --- | --- | --- | --- |
| All covariates | 962,702 | - | - |
| Birthweight removed | 962,729 | 26 | Keep |
| Gestational age removed | 962,707 | 5 | Keep |
| Mother's age at delivery removed | 962,742 | 39 | Keep |
| SEP removed | 962,987 | 284 | Keep |
| Sex removed | 963,445 | 743 | Keep |
| Rater removed | 977,235 | 14533 | Keep |
| Cohort*birthweight removed | 962,704 | 2 | Drop |
| **Cohort*gestational age removed** | **962,700** | **-4** | **Drop** |
| Cohort*age at delivery removed | 962,720 | 20 | Keep |
| Cohort*SEP removed | 962,735 | 35 | Keep |
| Cohort*sex removed | 962,799 | 98 | Keep |
| Cohort*rater removed | 966,796 | 4095 | Keep |
| Cohort*slope removed | 963,401 | 701 | Keep |

**Note.** We iteratively removed each covariate based upon the order in which they least changed the coefficient. If the AIC increased by more than 4 than the last best fitting model then we kept them in the model. The best fitting/most parsimonious model is highlighted in bold. SEP = socio-economic position.

### Table S24. The iterative removal of covariates from our model of DSM for males only

| **Model** | **AIC** | **AIC Difference** | **Decision** |
| --- | --- | --- | --- |
| All covariates | 460,956 | - | - |
| Birthweight removed | 460,961 | 5 | Keep |
| Gestational age removed | 460,953 | -3 | Drop |
| Mother's age at delivery removed | 460,969 | 17 | Keep |
| SEP removed | 461,069 | 117 | Keep |
| Rater removed | 465,802 | 4849 | Keep |
| **Cohort*birthweight removed** | **460,948** | **-5** | **Drop** |
| Cohort*age at delivery removed | 460,955 | 7 | Keep |
| Cohort*SEP removed | 634,329 | 173382 | Keep |
| Cohort*rater removed | 462,730 | 1782 | Keep |
| Cohort*slope removed | 461,223 | 275 | Keep |

**Note.** We iteratively removed each covariate based upon the order in which they least changed the coefficient. If the AIC increased by more than 4 than the last best fitting model then we kept them in the model. The best fitting/most parsimonious model is highlighted in bold. SEP = socio-economic position.

### Table S25. The iterative removal of covariates from our model of DSM for females only

| **Model** | **AIC** | **AIC Difference** | **Decision** |
| --- | --- | --- | --- |
| All covariates | 498,985 | - | - |
| Birthweight removed | 499,001 | 16 | Keep |
| Gestational age removed | 498,985 | 0 | Drop |
| Mother's age at delivery removed | 498,998 | 14 | Keep |
| SEP removed | 499,141 | 156 | Keep |
| Rater removed | 509,447 | 10462 | Keep |
| **Cohort*birthweight removed** | **498,977** | **-7** | **Drop** |
| Cohort*age at delivery removed | 498,984 | 7 | Keep |
| Cohort*SEP removed | 498,999 | 22 | Keep |
| Cohort*rater removed | 501,338 | 2361 | Keep |
| Cohort*slope removed | 499,429 | 452 | Keep |

**Note.** We iteratively removed each covariate based upon the order in which they least changed the coefficient. If the AIC increased by more than 4 than the last best fitting model then we kept them in the model. The best fitting/most parsimonious model is highlighted in bold. SEP = socio-economic position.

### Table S26. The iterative removal of covariates from our model of DSM for the low SEP group only

| **Model** | **AIC** | **AIC Difference** | **Decision** |
| --- | --- | --- | --- |
| All covariates | 338,887 | - | - |
| Birthweight removed | 338,888 | 1 | Drop |
| Gestational age removed | 338,881 | -7 | Drop |
| Mother's age at delivery removed | 338,900 | 18 | Keep |
| Sex removed | 339,161 | 280 | Keep |
| Rater removed | 343,387 | 4506 | Keep |
| **Cohort*age at delivery removed** | **338,877** | **-4** | **Drop** |
| Cohort*sex removed | 338,918 | 41 | Keep |
| Cohort*rater removed | 340,467 | 1590 | Keep |
| Cohort*slope removed | 339,092 | 214 | Keep |

**Note.** We iteratively removed each covariate based upon the order in which they least changed the coefficient. If the AIC increased by more than 4 than the last best fitting model then we kept them in the model. The best fitting/most parsimonious model is highlighted in bold. SEP = socio-economic position.

### Table S27. The iterative removal of covariates from our model of DSM for the high SEP group only

| **Model** | **AIC** | **AIC Difference** | **Decision** |
| --- | --- | --- | --- |
| All covariates | 453,686 | - | - |
| Birthweight removed | 453,702 | 15 | Keep |
| Gestational age removed | 453,684 | -2 | Drop |
| Mother's age at delivery removed | 453,695 | 10 | Keep |
| SEP removed | 454,049 | 365 | Keep |
| Rater removed | 460,873 | 7188 | Keep |
| **Cohort*birthweight removed** | **453,679** | **-5** | **Drop** |
| Cohort*age at delivery removed | 453,689 | 10 | Keep |
| Cohort*Sex removed | 453,700 | 21 | Keep |
| Cohort*rater removed | 455,538 | 1859 | Keep |
| Cohort*slope removed | 453,989 | 310 | Keep |

**Note.** We iteratively removed each covariate based upon the order in which they least changed the coefficient. If the AIC increased by more than 4 than the last best fitting model then we kept them in the model. The best fitting/most parsimonious model is highlighted in bold. SEP = socio-economic position.

### Table S28. The difference between predicted and observed values of DSM for the best fitting model in all cohorts

|  | **N** | **Observed Mean** | **Observed SD** | **Predicted Mean** | **Predicted SD** | **Mean Difference** | **SD Difference** | **Minimum Difference** | **Maximum Difference** |
| --- | --- | --- | --- | --- | --- | --- | --- | --- | --- |
| Overall | 118,144 | 16.63 | 17.94 | 17.11 | 7.05 | 0.48 | 16.42 | -93.24 | 40.87 |
| 5-7 | 4,369 | 24.34 | 22.46 | 24.07 | 8.62 | -0.27 | 20.27 | -86.20 | 39.30 |
| 7-9 | 20,927 | 18.41 | 18.93 | 18.80 | 5.85 | 0.38 | 17.81 | -88.05 | 37.61 |
| 9-11 | 14,994 | 16.77 | 18.98 | 16.67 | 6.07 | -0.10 | 17.77 | -87.87 | 35.15 |
| 11-13 | 12,359 | 17.37 | 17.96 | 17.98 | 5.62 | 0.61 | 16.90 | -82.02 | 34.11 |
| 13-15 | 19,340 | 16.41 | 16.97 | 17.08 | 6.58 | 0.68 | 15.79 | -88.92 | 32.69 |
| 15-17 | 12,652 | 12.36 | 14.72 | 13.03 | 4.22 | 0.67 | 14.07 | -87.82 | 24.93 |
| 17-19 | 4,940 | 19.84 | 21.80 | 20.30 | 10.25 | 0.46 | 19.34 | -93.12 | 40.87 |
| 19-21 | 916 | 14.38 | 14.56 | 13.73 | 2.95 | -0.65 | 14.70 | -76.06 | 19.35 |
| 21-23 | 12,533 | 19.40 | 17.31 | 20.06 | 6.95 | 0.65 | 15.84 | -85.96 | 31.30 |
| 23-25 | 5,983 | 17.27 | 15.34 | 17.95 | 6.46 | 0.68 | 14.14 | -77.45 | 28.70 |
| 25-27 | 7,481 | 7.99 | 12.63 | 9.19 | 5.02 | 1.20 | 11.90 | -89.04 | 21.37 |
| 27-29 | 12 | 35.65 | 40.05 | 8.38 | 4.96 | -27.27 | 39.23 | -93.24 | 10.50 |
| 38 | 833 | 9.30 | 14.29 | 9.34 | 2.39 | 0.04 | 14.36 | -77.50 | 16.17 |
| 45 | 805 | 8.83 | 14.06 | 8.92 | 2.40 | 0.09 | 14.18 | -81.68 | 15.74 |

**Note.** The best fitting model has linear splines with knots at 14, 17 and 21 years. Predicted values are based upon a model with all cohorts, raters and optimum covariates included. SD = standard deviation.

### Table S29. Coefficients for the best fitting model of DSM scores

|  |  | Centred at the mean for the total sample | Centred at the mean for each cohort | GEE Results |
| --- | --- | --- | --- | --- |
|  |  | **Coefficient (95% CI)** | **Coefficient (95% CI)** | **Coefficient (95% CI)** |
| **Fixed Effects** | Intercept | 25.255 (24.729, 25.780) | 25.645 (25.140, 26.149) | 25.026 (24.488, 25.563) |
|  | Spline 1 (Age 5-14 years) | -0.704 ( -0.769, -0.640) | -0.704 (-0.769, -0.640) | -0.775 (-0.842, -0.708) |
|  | Spline 2 (Age 14-17 years) | -1.321 (-1.471, -1.170) | -1.321 (-1.471, -1.170) | -1.436 (-1.582, -1.291) |
|  | Spline 3 (Age 17-21 years) | 0.455 (0.324, 0.587) | 0.455 (0.324, 0.587) | 0.644 (0.510, 0.778) |
|  | Spline 4 (Age 21+ years) | -0.826 (-1.065, -0.588) | -0.826 ( -1.065, -0.588) | -1.033 ( -1.286, -0.780) |
|  | Rater (self) | 10.790 (10.512, 11.067) | 10.790 (10.512, 11.067) | 10.583 (10.277, 10.889) |
|  | Rater (teacher) | -2.101 (-2.809, -1.394) | -2.101 ( -2.809, -1.394) | -1.576 (-2.471, -0.681) |
|  | Cohort (ALSPAC) | -8.221 (-9.054, -7.389) | -8.599 ( -9.382, -7.817) | -7.917 (-8.824, -7.011) |
|  | Cohort (Pelotas) | 4.566 (1.901, 7.230) | 4.492 (1.861, 7.124) | 5.276 (1.738, 8.813) |
|  | Cohort (E-Risk) | 8.626 (7.462, 9.789) | 8.983 (7.835, 10.131) | 9.547 (8.316, 10.777) |
|  | Cohort (Dunedin) | 0.897 (-1.014, 2.808) | 1.164 (-0.627, 2.954) | 2.588 (0.527, 4.650) |
|  | Sex (female) | -4.934 (-5.378, -4.489) | -4.934 (-5.378, -4.489) | -5.004 (-5.417, -4.591) |
|  | Birthweight | -0.994 (-1.370, -0.619) | -0.994 (-1.370, -0.619) | -1.196 (-1.597, -0.796) |
|  | Gestational age | 0.087 (-0.006, 0.179) | 0.087 (-0.006, 0.179) | 0.094 (-0.004, 0.192) |
|  | Mother’s age at delivery | 0.036 (-0.021, 0.094) | 0.036 (-0.021, 0.094) | 0.072 (0.018, 0.126) |
|  | SEP | -1.720 (-1.979, -1.460) | -1.720 (-1.979, -1.460) | -1.689 (-1.929, -1.450) |
|  | Rater (self) * Cohort (ALSPAC) | -1.909 (-2.513, -1.305) | -1.909 (-2.513, -1.305) | -1.339 (-1.949, -0.729) |
|  | Rater (self) * Cohort (E-Risk) | 7.795 (6.963, 8.627) | 7.795 (6.963, 8.627) | 8.443 (7.243, 9.643) |
|  | Rater (teacher) * Cohort (E-Risk) | -13.508 (-14.306, -12.710) | -13.508 (-14.306, -12.710) | -14.113 (-15.258, -12.968) |
|  | Rater (self) * Cohort (Dunedin) | -12.970 (-13.677, -12.263) | -12.970 (-13.677, -12.263) | -13.001 (-14.058, -11.945) |
|  | Rater (Teacher) * Cohort (Dunedin) | -1.499 (-2.456, -0.542) | -1.499 (-2.456, -0.542) | -1.957 ( -3.335, -0.580) |
|  | Cohort (ALSPAC) * Sex (Female) | 0.472 (-0.254, 1.198) | 0.472 (-0.254, 1.198) | 0.427 (-0.292, 1.147) |
|  | Cohort (Pelotas) * Sex (Female) | 5.756 (4.612, 6.900) | 5.756 (4.612, 6.900) | 5.811 (4.382, 7.240) |
|  | Cohort (E-Risk) * Sex (Female) | -0.340 (-1.690, 1.011) | -0.340 (-1.690, 1.011) | -1.923 (-3.153, -0.692) |
|  | Cohort (Dunedin) * Sex (Female) | 2.279 (0.926, 3.632) | 2.279 (0.926, 3.632) | -0.273 (-1.606, 1.061) |
|  | Cohort (ALSPAC) * Mother’s age | -0.193 (-0.279, -0.107) | -0.193 (-0.279, -0.107) | -0.195 (-0.284, -0.107) |
|  | Cohort (Pelotas) * Mother’s age | -0.133 (-0.234, -0.033) | -0.133 (-0.234, -0.033) | -0.170 (-0.290, -0.049) |
|  | Cohort (E-Risk) * Mother’s age | -0.218 (-0.350, -0.085) | -0.218 (-0.350, -0.085) | -0.301 (-0.418, -0.184) |
|  | Cohort (Dunedin) * Mother’s age | -0.252 (-0.398, -0.105) | -0.252 (-0.398, -0.105) | -0.278 ( -0.431, -0.124) |
|  | Cohort (ALSPAC) * SEP | 0.788 (0.386, 1.190) | 0.788 (0.386, 1.190) | 0.926 (0.510, 1.341) |
|  | Cohort (Pelotas) * SEP | 1.177 (0.581, 1.773) | 1.177 (0.581, 1.773) | 1.157 (0.429, 1.885) |
|  | Cohort (E-Risk) * SEP | -1.195 (-1.937, -0.452) | -1.195 (-1.937, -0.452) | -1.172 (-1.841, -0.502) |
|  | Cohort (Dunedin) * SEP | 0.176 (-0.536, 0.889) | 0.176 (-0.536, 0.889) | -0.580 (-1.275, 0.114) |
|  | Spline1 * Cohort (ALSPAC) | 0.368 (0.271, 0.464) | 0.368 (0.271, 0.464) | 0.399 (0.301, 0.497) |
|  | Spline1 * Cohort (E-Risk) | -0.141 (-0.244, -0.038) | -0.141 (-0.244, -0.038) | -0.071 (-0.192, 0.049) |
|  | Spline1 * Cohort (Dunedin) | -0.502 (-0.717, -0.286) | -0.502 (-0.717, -0.286) | -0.469 (-0.710, -0.228) |
|  | Spline2 * Cohort (ALSPAC) | -0.009 (-0.371, 0.354) | -0.009 (-0.371, 0.354) | -0.017 (-0.368, 0.335) |
|  | Spline2 * Cohort (E-Risk) | -2.338 (-2.633, -2.043) | -2.338 (-2.633, -2.043) | -2.444 (-2.814, -2.075) |
|  | Spline2 * Cohort (Dunedin) | 6.030 (5.167, 6.893) | 6.030 (5.167, 6.893) | 6.382 (5.449, 7.315) |
|  | Spline3 * Cohort (ALSPAC) | -0.504 (-1.375, 0.367) | -0.504 (-1.375, 0.367) | -1.414 (-2.397, -0.431) |
|  | Spline3 * Cohort (Dunedin) | -4.510 (-5.491, -3.528) | -4.510 (-5.491, -3.528) | -4.715 (-5.607, -3.824) |
|  | Spline4 * Cohort (ALSPAC) | 0.598 (-0.139, 1.335) | 0.598 (-0.139, 1.335) | 1.310 (0.475, 2.144) |
|  | Spline4 * Cohort (Pelotas) | -2.216 (-3.801, -0.632) | -2.216 (-3.801, -0.632) | -2.014 (-4.123, 0.095) |
|  | Spline4 * Cohort (Dunedin) | 0.764 (0.472, 1.056) | 0.764 (0.472, 1.056) | 0.965 (0.684, 1.246) |
| **Random Effects (Level 3)** | Variance Intercept | 57.996 (50.066, 65.925) | 57.996 (50.066, 65.925) |  |
| **Random Effects (Level 2)** | Variance Intercept | 164.657 (153.640, 175.675) | 164.657 (153.640, 175.675) |  |
|  | Covariance Intercept and Spline 1 | -7.994 (-8.992, -6.996) | -7.994 (-8.992, -6.996) |  |
|  | Variance Spline 1 | 0.971 (0.825, 1.117) | 0.971 (0.825, 1.117) |  |
|  | Covariance Intercept and Spline 2 | -13.354 (-15.704, -11.003) | -13.354 (-15.704, -11.003) |  |
|  | Covariance Spline 1 and Spline 2 | 0.544 (0.235, 0.853) | 0.544 (0.235, 0.853) |  |
|  | Variance Spline 2 | -1.339 (-2.346, -0.331) | -1.339 (-2.346, -0.331) |  |
|  | Covariance Intercept and Spline 3 | -3.512 (-5.377, -1.647) | -3.512 (-5.377, -1.647) |  |
|  | Covariance Spline 1 and Spline 3 | -0.113 (-0.347, 0.121) | -0.113 (-0.347, 0.121) |  |
|  | Covariance Spline 2 and Spline 3 | 8.398 (7.775, 9.022) | 8.398 (7.775, 9.022) |  |
|  | Variance Spline 3 | -7.572 (-8.150, -6.993) | -7.572 (-8.150, -6.993) |  |
|  | Covariance Intercept and Spline 4 | -5.660 (-6.707, -4.613) | -5.660 (-6.707, -4.613) |  |
|  | Covariance Spline 1 and Spline 4 | 0.234 (0.099, 0.368) | 0.234 (0.099, 0.368) |  |
|  | Covariance Spline 2 and Spline 4 | -2.610 (-3.130, -2.089) | -2.610 (-3.130, -2.089) |  |
|  | Covariance Spline 3 and Spline 4 | 1.245 (0.874, 1.615) | 1.245 (0.874, 1.615) |  |
|  | Variance Spline 4 | 0.535 (0.431, 0.640) | 0.535 (0.431, 0.640) |  |
| **Random Effects (Level 1)** | Variance Intercept | 133.069 (131.511, 134.626) | 133.069 (131.511, 134.626) |  |

**Note.** The best fitting model has linear splines with knots at 14, 17 and 21 years. All five cohorts (TEDS, ALSPAC, E-Risk, Pelotas, Dunedin) and three raters (parent, teacher, self) were included. Estimates are given for parent-ratings of a male from the TEDS cohort. The primary model reported in the main text has all continuous covariates centred to the mean for the total sample (birthweight = 3kg, gestational age = 38 weeks, mother’s age at delivery = 30 years). This is compared with a model where each cohort had covariates centred to the mean for that cohort (covariate means by cohort available in Supplementary Table S1). SEP = socio-economic position.

## Supplementary Figures

### Figure S1. Cubic spline model for SDQ in the ALSPAC cohort


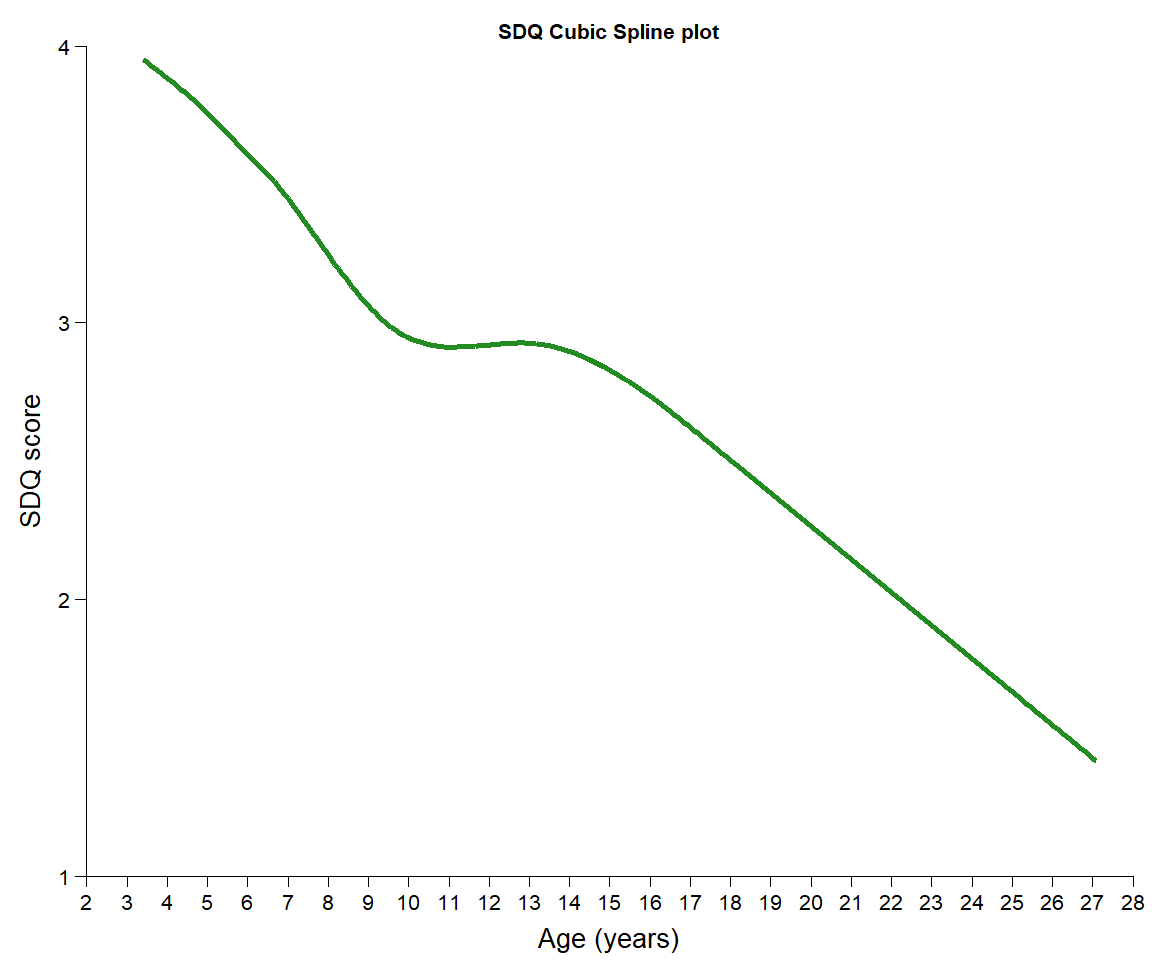


### Figure S2. Cubic spline model for SDQ in the TEDS cohort


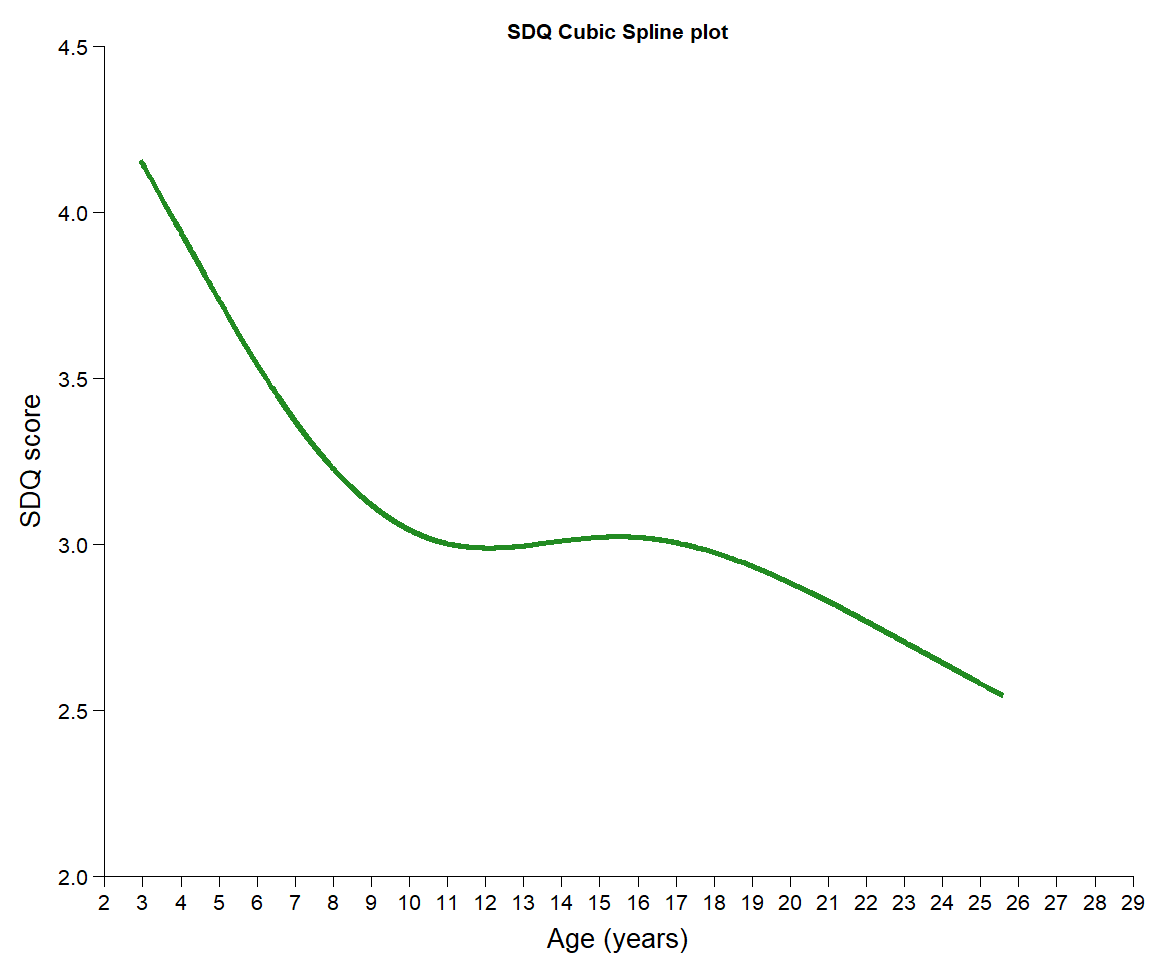


### Figure S3. SDQ Trajectories extrapolated from age 3 to 27 years


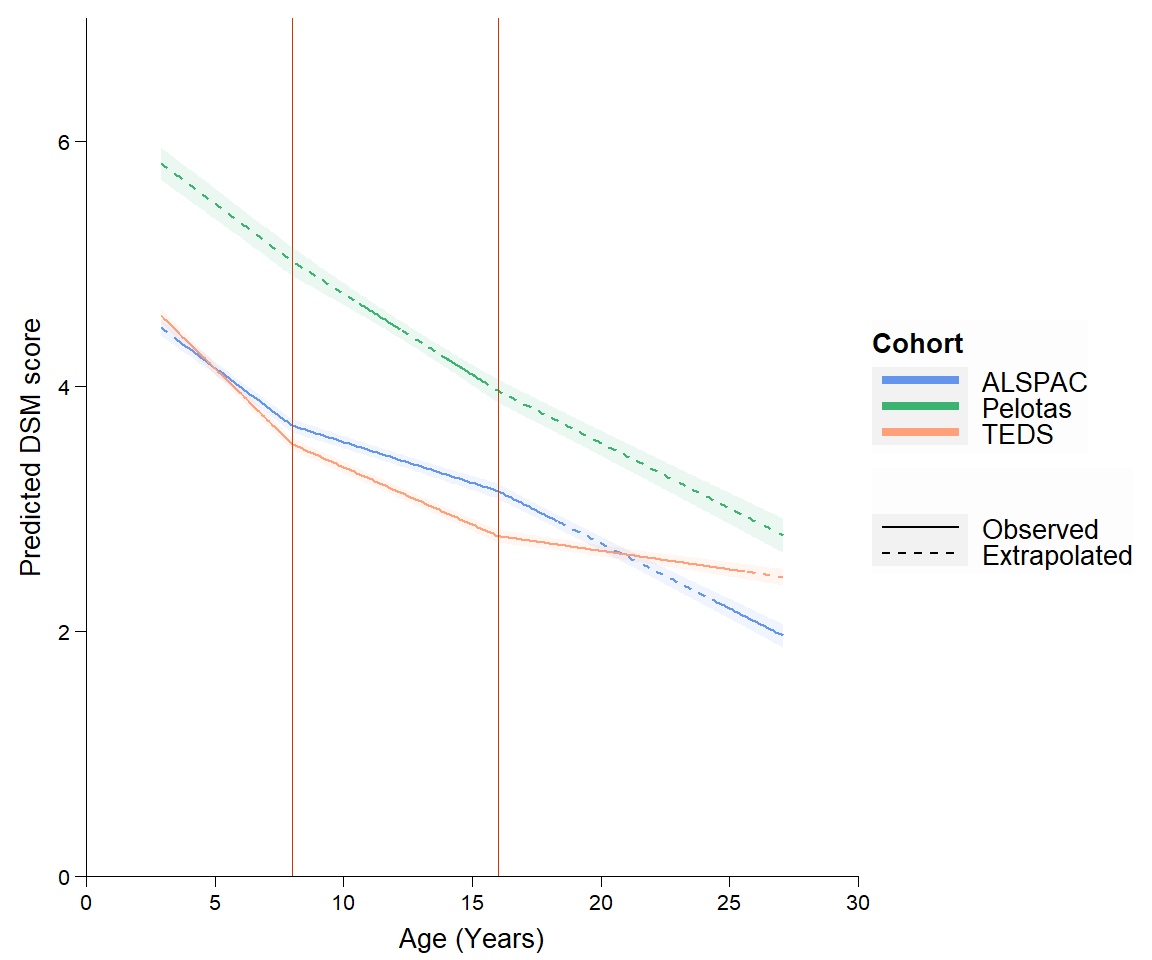
 **Note.** The best fitting model of SDQ (knot points at 8 and 16 years) extrapolated across ages 3 to 27 years. Gaps between observed ages within each cohort are represented as extrapolated if they exceed 1 year between observations. Plotted average scores are parent-rated for a male, with mean covariate values.

### Figure S4. Benchmark Model of DSM differences across cohorts


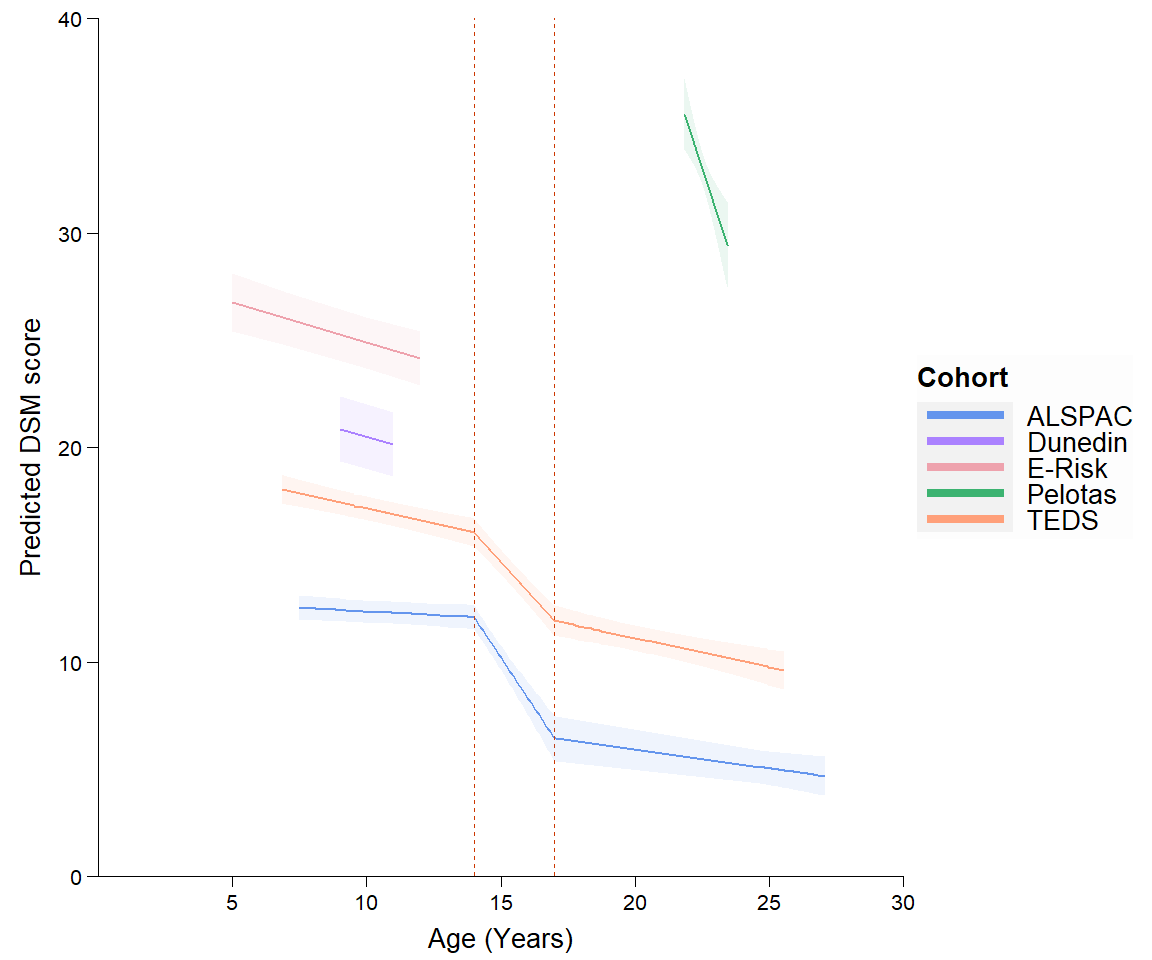


### Figure S5. Cubic spline model for DSM in the TEDS cohort


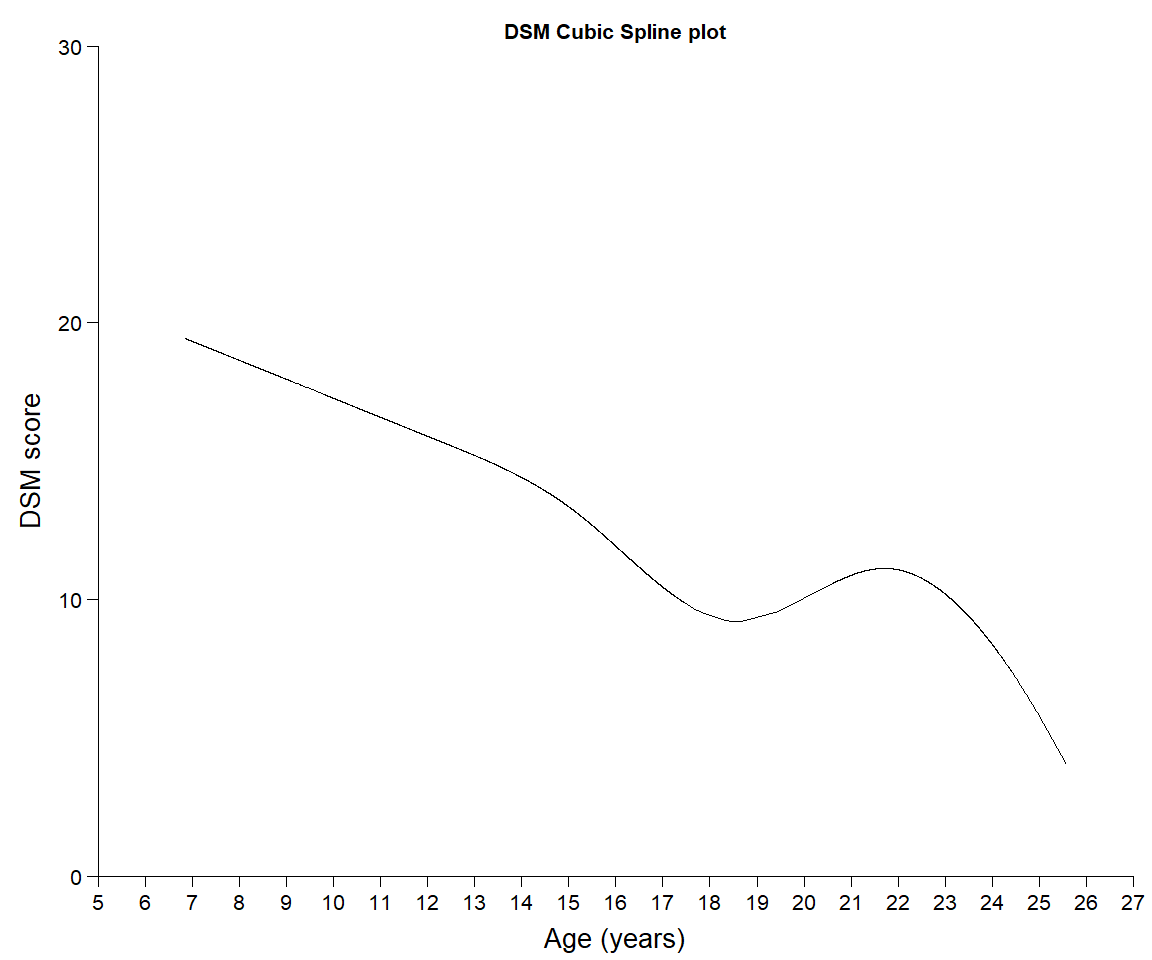


### Figure S6. DSM Trajectories extrapolated from age 5 to 45 years


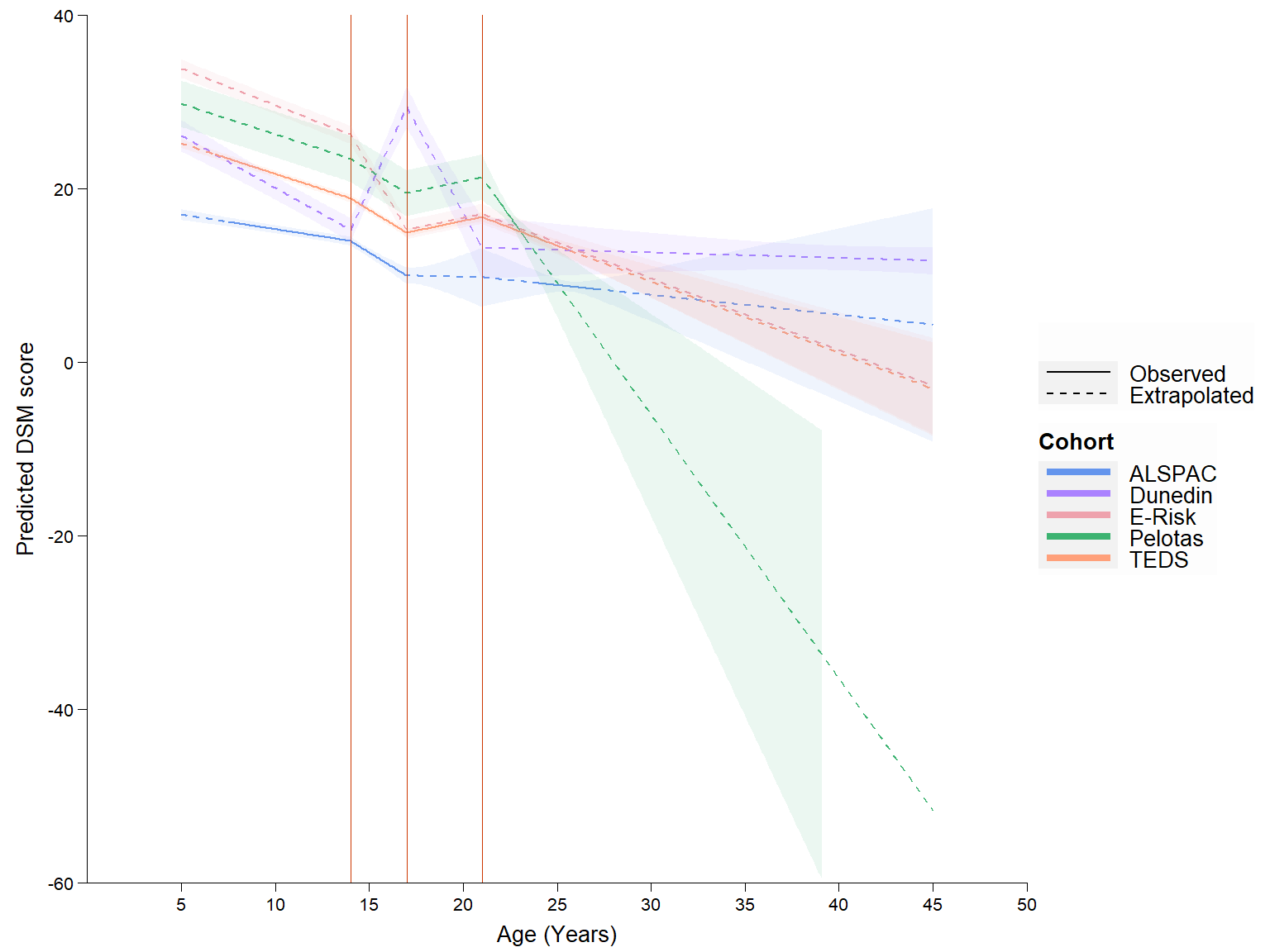
 **Note.** The best fitting model of DSM (knot points at 14, 17 and 21 years) with predicted values extrapolated from age 5 to 45 years, even where data is unobserved in a particular cohort. Gaps between observed ages within each cohort are represented as extrapolated if they exceed 1 year between observations. Predicted values are parent rated for a male with average covariate values. Values below zero are not theoretically possible - the model is creating less accurate predictions at the extreme of the age distribution due to smaller numbers of observations in older ages. Values at extreme ages should be interpreted with caution.

### Figure S7. Average SDQ scores split by sex


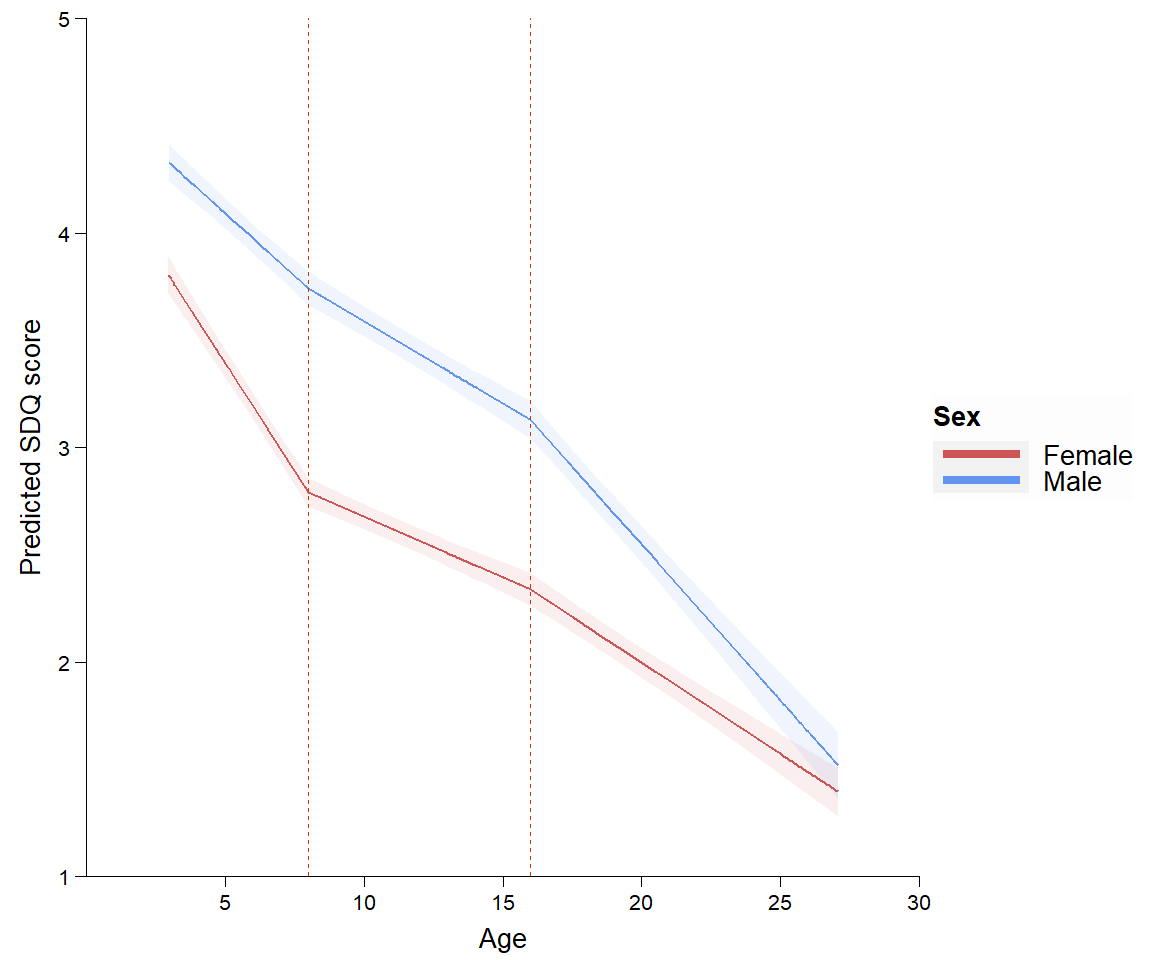
 **Note.** The best fitting model with knot points at 8 and 16 years is adjusted for cohort, rater, birthweight, mother’s age at delivery, socio-economic position. Includes interactions between cohort and rater, mother’s age at delivery, socio-economic position and slope.

### Figure S8. Average DSM percentage scores split by sex


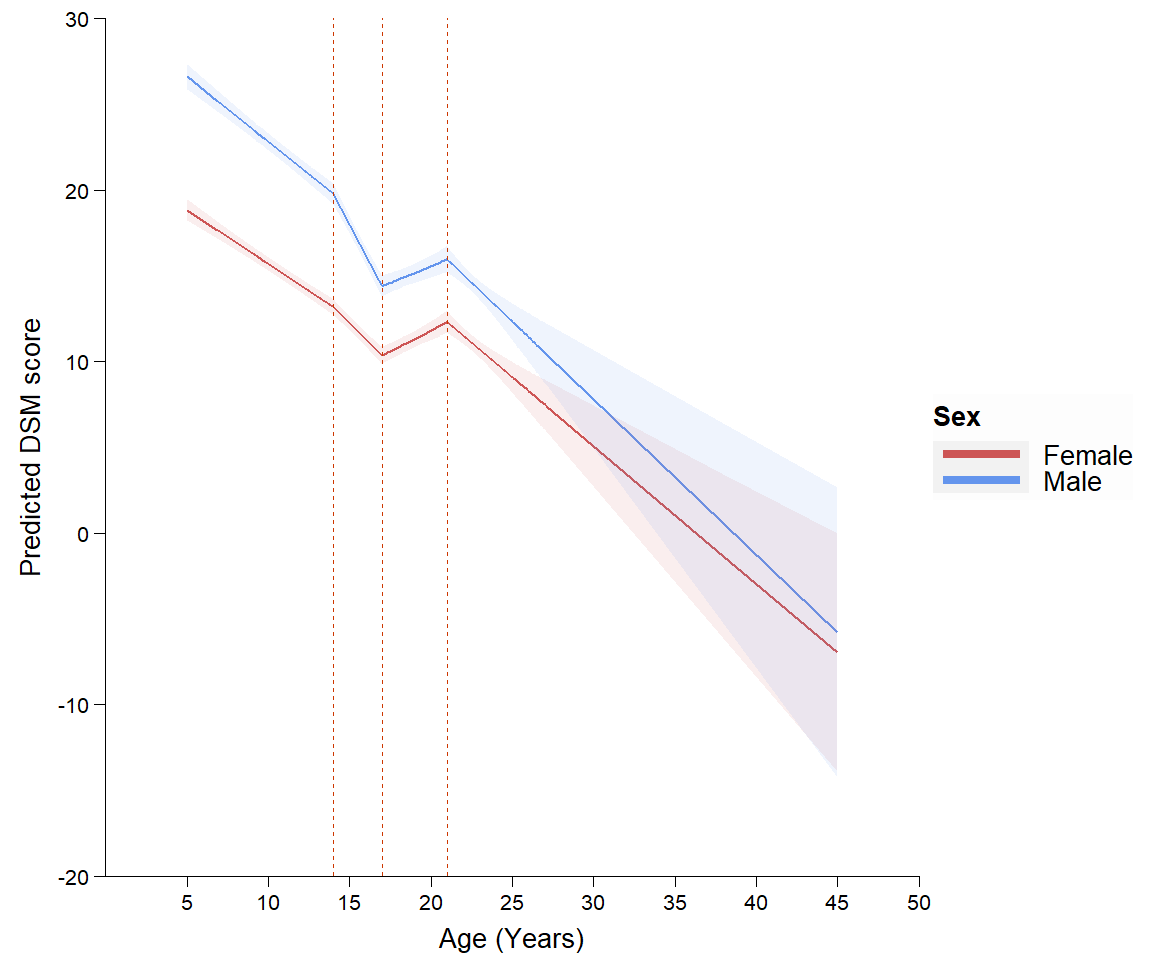
 **Note.** The best fitting model with knot points at 14, 17 and 21 years is adjusted for cohort, rater, birthweight, mother’s age at delivery, socio-economic position. Includes interactions between cohort and rater, mother’s age at delivery, socio-economic position and slope. Plotted values are parent-rated for an individual from the TEDS cohort with average covariate values. Values below zero are not theoretically possible - the model is creating less accurate predictions at the extreme of the age distribution due to smaller numbers of observations in older ages. Values at extreme ages should be interpreted with caution.

### Figure S9. Average SDQ scores split by socio-economic position


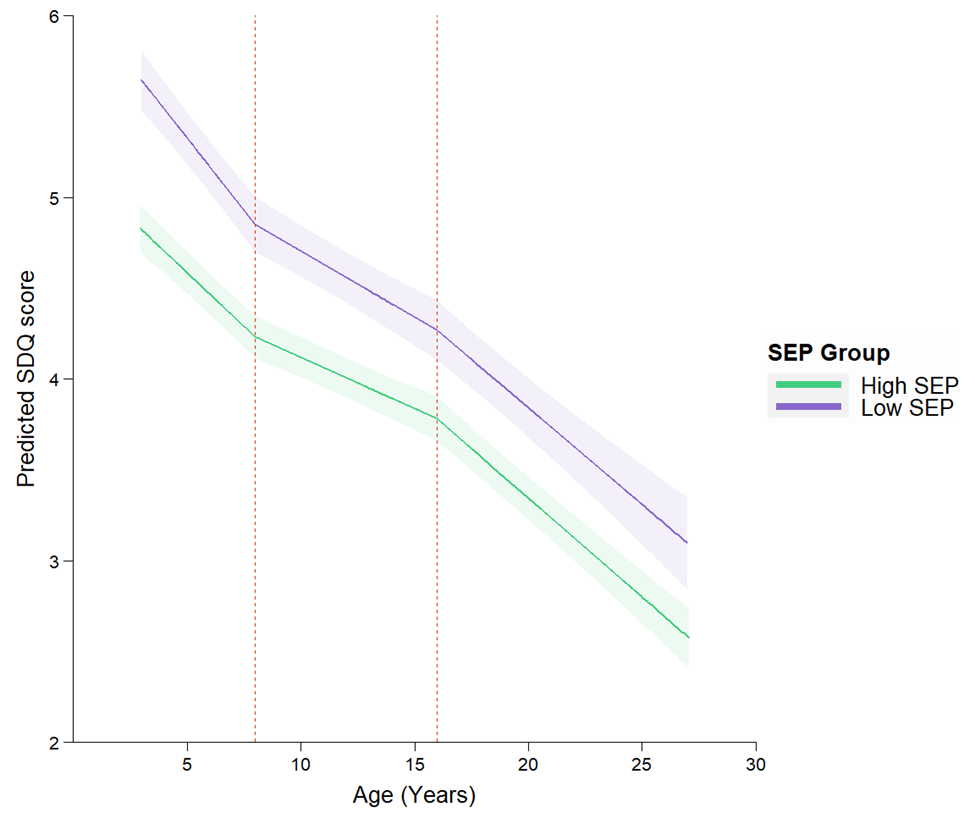


**Note.** High and Low SEP are median split for each cohort. Plotted average scores are parent-rated for a male from the ALSPAC cohort with average covariates. High and Low SEP are median split for each cohort. The plotted final model has knot points at 8 and 16 years and is adjusted for cohort, rater, birthweight, mother’s age at delivery and sex. It includes interactions between cohort and rater, age at delivery, sex and slope.

### Figure S10. Average DSM percentage scores split by socio-economic position


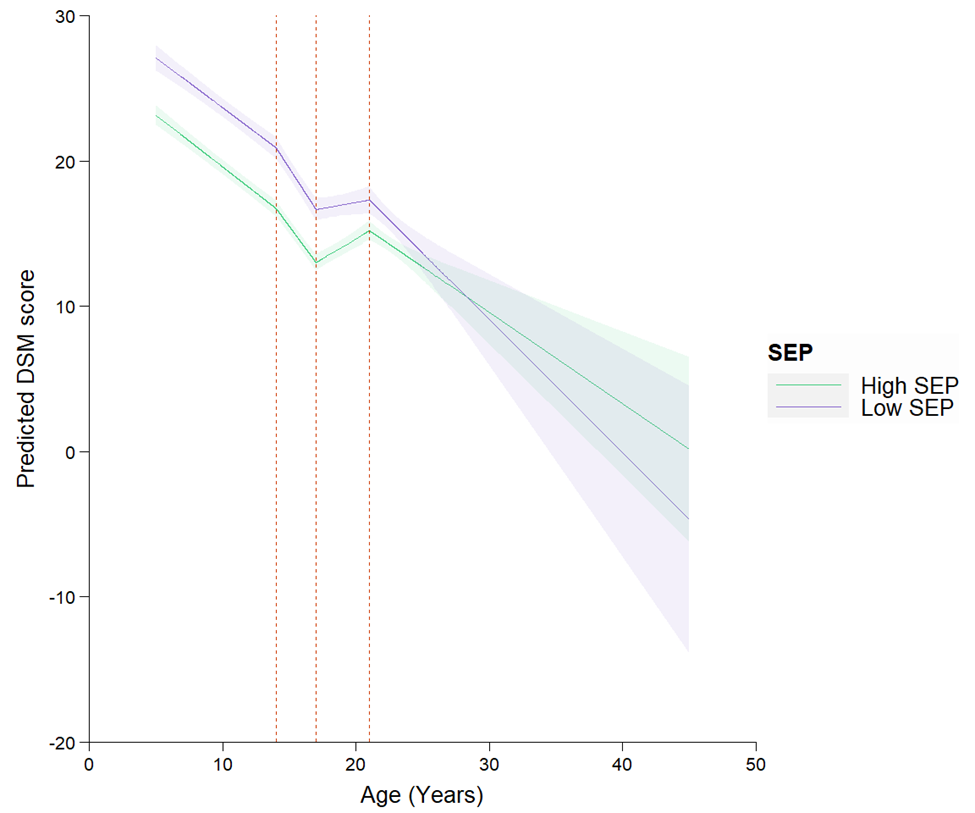


**Note.** High and Low SEP are median split for each cohort. Plotted average scores are parent-rated for a male from the TEDS cohort with average covariates. High and Low SEP are median split for each cohort. The plotted final model has knot points at 14, 17 and 21 years and is adjusted for cohort, rater, birthweight, mother’s age at delivery and sex. Includes interactions between cohort and rater, sex, mother’s age at delivery and slope. Values below zero are not theoretically possible - the model is creating less accurate predictions at the extreme of the age distribution due to smaller numbers of observations in older ages. Values at extreme ages should be interpreted with caution.

### Figure S11. SDQ Model using only individuals who have completed 3+ assessments across the age range


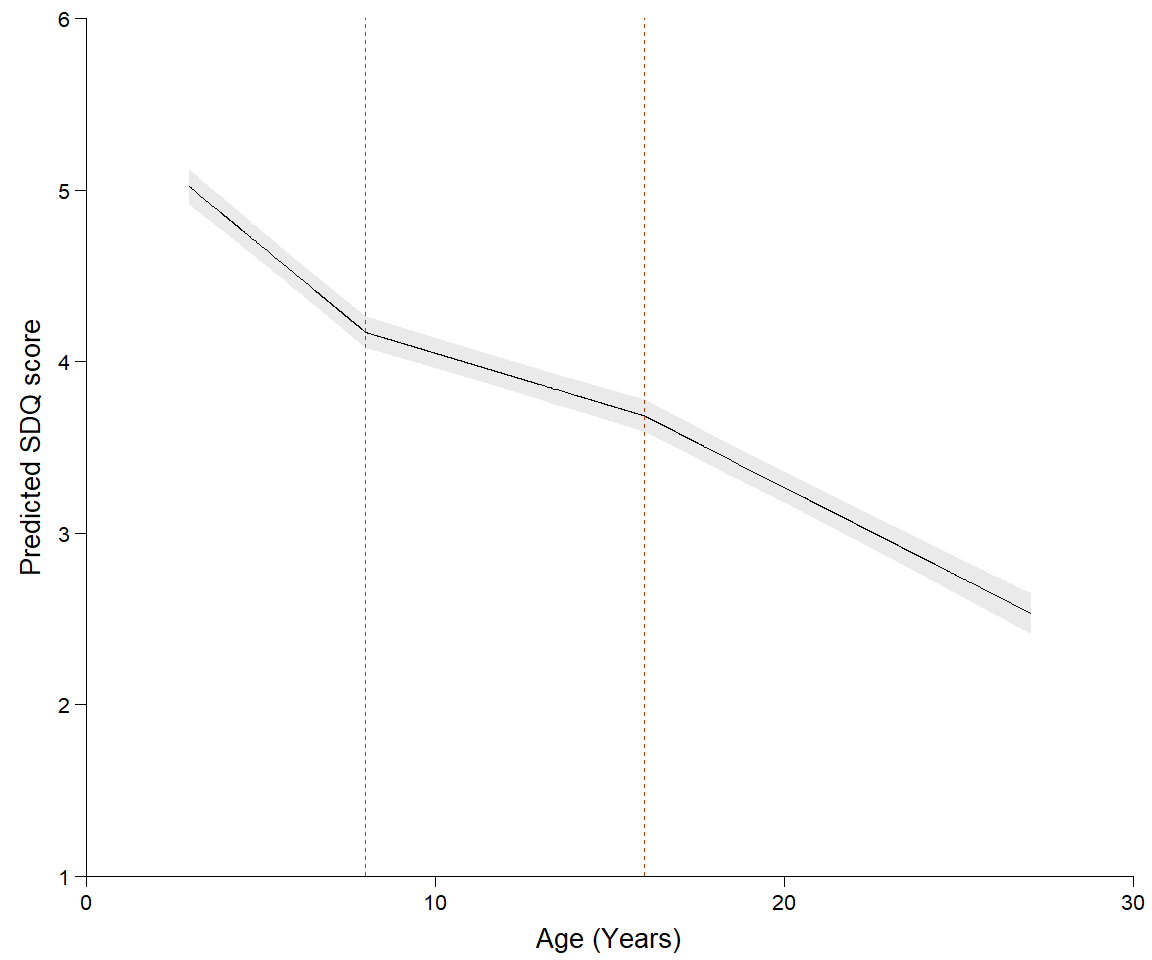
 **Note.** The best fitting model with knot points at 8 and 16 years is adjusted for cohort, rater, birthweight, mother’s age at delivery, socio-economic position. Includes interactions between cohort and rater, mother’s age at delivery, socio-economic position and slope.

### Figure S12. DSM Model using only individuals who have completed 3+ assessments across the age range


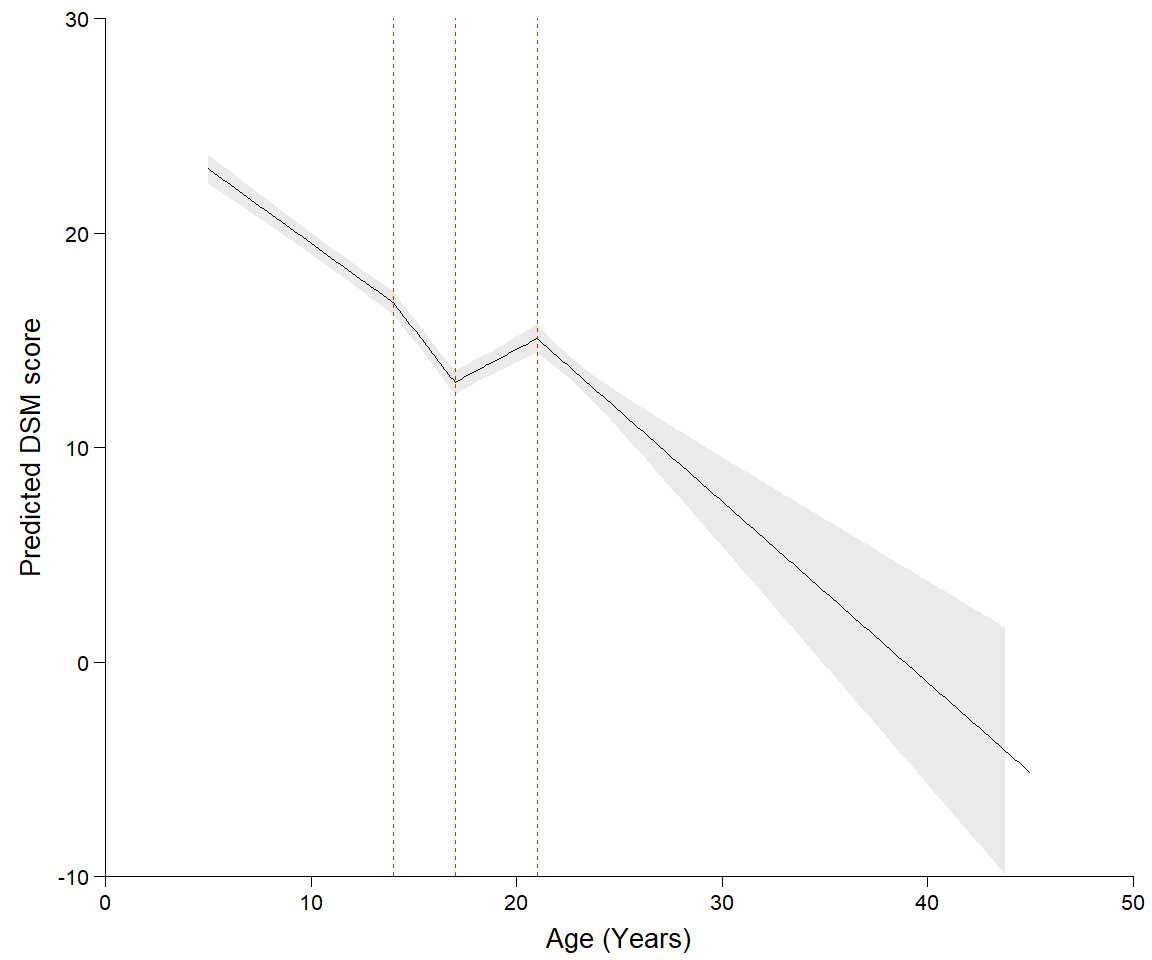
 **Note.** The best fitting model with knot points at 14, 17 and 21 years is adjusted for cohort, rater, birthweight, mother’s age at delivery, socio-economic position. Includes interactions between cohort and rater, mother’s age at delivery, socio-economic position and slope. Values below zero are not theoretically possible - the model is creating less accurate predictions at the extreme of the age distribution due to smaller numbers of observations in older ages. Values at extreme ages should be interpreted with caution.

### Figure S13. Exploratory analysis removing TEDS self-ratings at age 21 years


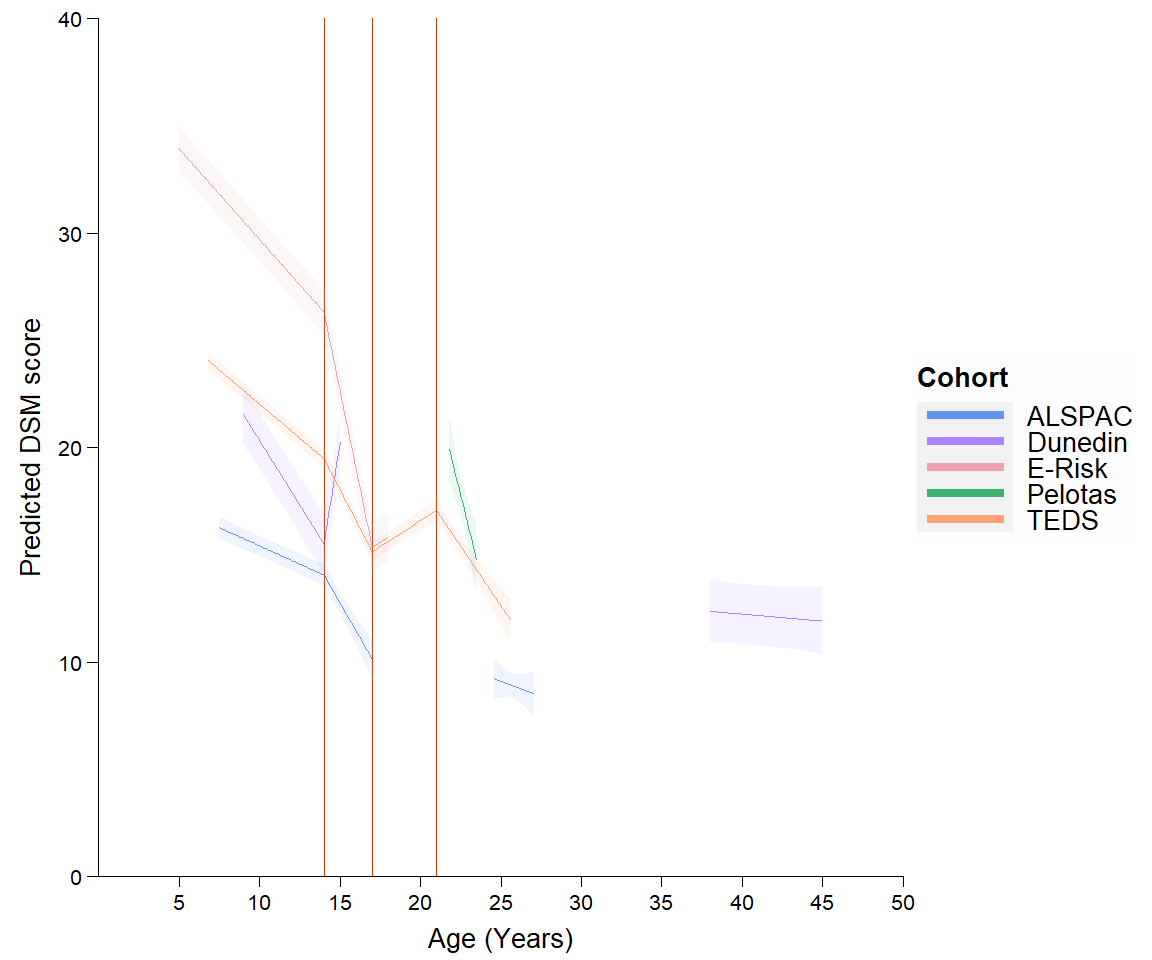
 **Note.** The best fitting model with knot points at 14, 17 and 21 years is adjusted for cohort, rater, birthweight, mother’s age at delivery, socio-economic position. Includes interactions between cohort and rater, mother’s age at delivery, socio-economic position and slope.
