# Supplementary material for: Decline in attention-deficit hyperactivity disorder traits over the life course in the general population: trajectories across five population birth cohorts spanning ages 3 to 45 years
Source: Int J Epidemiol. 2022 Apr 11;51(3):919–30. doi: 10.1093/ije/dyac049 (PMC9189965; doi:10.1093/ije/dyac049)
Supplement: dyac049_Supplementary_Data [file dyac049_supplementary_data.zip › ije-2021-08-1228-File011.docx]

**Supplementary Note**

**Contents**

| 1. Cohort Descriptions | Page 2 |
| --- | --- |
| 2. Overview of ADHD Measures collected in each of the cohorts | Page 4 |
| 3. Measures of DSM 5 diagnostic items for ADHD across the five cohorts. | Page 5 |
| 4: Details of the ADHD trait measures | Page 8 |
| 5. Measures of covariates across cohorts | Page 10 |
| 6. Benchmark Model of DSM Differences across Cohorts | Page 12 |
| 7. Sensitivity analysis to explore the role of attrition on patterns of change | Page 13 |
| 8. The results of sex split analysis for each model | Page 14 |
| 9. The results of SEP split analysis for each model | Page 16 |
| 10. Exploration of the average increase in DSM score at ages 17 and 21 | Page 18 |
| 11. Additional methodological details  References | Page 19  Page 21 |
|  |  |

**Supplementary Note 1. Cohort Descriptions**

ALSPAC

Pregnant women resident in Avon, UK with expected dates of delivery 1st April 1991 to 31st December 1992 were invited to take part in the study. The initial number of pregnancies enrolled is 14,541 (for these at least one questionnaire has been returned or a “Children in Focus” clinic had been attended by 19/07/99). Of these initial pregnancies, there was a total of 14,676 foetuses, resulting in 14,062 live births and 13,988 children who were alive at 1 year of age. When the oldest children were approximately 7 years of age, an attempt was made to bolster the initial sample with eligible cases who had failed to join the study originally. As a result, when considering variables collected from the age of seven onwards (and potentially abstracted from obstetric notes) there are data available for more than the 14,541 pregnancies mentioned above. The number of new pregnancies not in the initial sample (known as Phase I enrolment) that are currently represented on the built files and reflecting enrolment status at the age of 24 is 913 (456, 262 and 195 recruited during Phases II, III and IV respectively), resulting in an additional 913 children being enrolled. The phases of enrolment are described in more detail in the cohort profile paper and its update (Boyd et al., 2013; Fraser et al., 2012; Northstone et al., 2019). The total sample size for analyses using any data collected after the age of seven is therefore 15,454 pregnancies, resulting in 15,589 foetuses. Of these 14,901 were alive at 1 year of age. A 10% sample of the ALSPAC cohort, known as the Children in Focus (CiF) group, attended clinics at the University of Bristol at various time intervals between 4 to 61 months of age. The CiF group were chosen at random from the last 6 months of ALSPAC births (1432 families attended at least one clinic). Excluded were those mothers who had moved out of the area or were lost to follow-up, and those partaking in another study of infant development in Avon.

Study data after 22 years of age were collected and managed using REDCap electronic data capture tools hosted at the University of Bristol (Harris et al., 2009, 2019). Please note that the study website contains details of all the data that is available through a fully searchable data dictionary and variable search tool (<http://www.bristol.ac.uk/alspac/researchers/our-data/> ).

TEDS

The Twins Early Development Study (TEDS) sample is a longitudinal birth cohort of twins born between 1994 and 1996 in England and Wales. Initially, 16,810 pairs of twins were recruited and more than 10,000 pairs of twins are still enrolled. The primary research focus was behavioural and cognitive outcomes (Haworth et al., 2013). The TEDS sample was representative of the UK population at first contact (Haworth et al., 2013) and remains representative despite attrition (Kovas et al., 2007).

Twins were excluded from analysis if they met certain perinatal conditions: low birth weight, short gestational age, maternal drinking during pregnancy, long period of special care after birth, and long stay in hospital after birth. Individuals were also excluded if they do not meet general medical inclusion criteria: autism, cerebral palsy, any genetic, chromosomal or inherited disorder, brain damage, Downs syndrome, deafness, global development delay, blindness or death of either twin.

E-Risk

The Environmental Risk (E-Risk) Longitudinal Twin Study is a subset of the Twins Early Development Study, which aims to understand how environmental and genetic factors contribute to the development of behaviours, health, and mental health (Moffitt & E-Risk Study Team, 2002). Twins were selected to take part in the E-Risk study in order to form a nationally representative sample of newborns in the UK in the 1990s. This was achieved by focusing on the recruitment of twins from disadvantaged families, to avoid the typical bias present in population birth cohorts. This is to avoid an excess of twins born to well-educated women using assisted reproduction. The resulting study sample consists of 2,232 same-sex twins born in 1994-1995, who represent the full range of socioeconomic conditions in Great Britain (as measured by a neighbourhood-level socioeconomic index (ACORN)).

Pelotas

The 1993 Pelotas cohort is a longitudinal birth cohort recruited from the city of Pelotas in southern Brazil, a municipality in the state of Rio Grande do Sul. The population of Pelotas is around 350,000. All live babies born in Pelotas from 1 January to 31 December 1993 (*N* = 5265) were enrolled in the study (Gonçalves et al., 2018; Victora et al., 2008). Of those, 5249 families agreed to take part in the longitudinal study. This study was particularly focused on the effects of breast-feeding, intrauterine, infant and child growth on long-term health.

Dunedin

Dunedin is a city in New Zealand, at the head of Otago Harbour on the South Island’s southeast coast. It has a population of around 130,500 people. The original Dunedin cohort comprised 1037 children representing the general population of New Zealand’s South Island in the early 1970s (1972-1973), as measured using the New Zealand census. Participants were recruited from the greater Dunedin Metropolitan area, 3 years after their birth at Queen Mary Maternity Hospital—the only maternity hospital in Dunedin at the time (Poulton et al., 2015). The 9% who declined or were unable to participate were no different from the 91% who agreed to take part in terms of maternal prenatal complications, birthweight, neonatal complications or family socioeconomic status. Cohort members were primarily white: 7.5 % self-identify as being Māori which matches the ethnic distribution of the South Island of New Zealand.

**Supplementary Note 2. Overview of ADHD Measures collected in each of the cohorts**

| Age (years) | **4** | **5** | **7** | **8** | **9** | **10** | **11** | **12** | | **13** | | **14** | | **15** | **16** | **17** | | | **18** | **21** | | **25** | **38** | **45** | **Items** | **Scale** | | **Timescale** | | | | **Questionnaire** | | | | |  |  |
| --- | --- | --- | --- | --- | --- | --- | --- | --- | --- | --- | --- | --- | --- | --- | --- | --- | --- | --- | --- | --- | --- | --- | --- | --- | --- | --- | --- | --- | --- | --- | --- | --- | --- | --- | --- | --- | --- | --- |
| **1. SDQ (Hyperactivity subscale)** | | | | | | | | | | | | | | | | | | | | | | | | |  | | | | | |  | | | |  | | |  |
| ALSPAC | **P** |  | **P,T** | **P** |  | **P,T** |  | **P** | | **P** | |  | |  |  | | **P** |  | |  | **P,S** | |  |  | 5 | 0-2 | | 6 months | | | | SDQ | | | | |  |  |
| E-RISK |  |  |  |  |  |  |  |  | |  | |  | |  |  | |  |  | |  |  | |  |  |  |  | |  | | | |  | | | | |  |  |
| Pelotas |  |  |  |  |  |  | **P,S** |  | |  | |  | | **P** |  | |  |  | |  |  | |  |  | 5 | 0-2 | | 6 months | | | | SDQ | | | | |  |  |
| Dunedin |  |  |  |  |  |  |  |  | |  | |  | |  |  | |  |  | |  |  | |  |  |  |  | |  | | | |  | | | | |  |  |
| TEDS | **P** |  | **P,T** |  | **P,S,T** |  |  | **P,T,S** | |  | |  | |  | **P,S** | |  |  | | **P,S** |  | |  |  | 5 | 1-3 | | NA | | | | SDQ | | | | |  |  |
| **2. DSM 5 Items** | | | | | | | | | | | | | | | | | | | | | | | | |  | | | |  | | | |  | | |  |  |  |
| ALSPAC |  |  |  |  |  |  |  |  | |  | |  | |  |  | |  |  | |  | **P,S** | |  |  | 18 | 0-3 | | 6 months | | | | Barkley | | | | |  |  |
| ALSPAC |  |  |  | **P** |  |  | **P** |  | |  | | **P** | |  | **P** | |  |  | |  | **P** | |  |  | 18 | 1-3 | | 6 months | | | | DAWBA | | | | |  |  |
| E-RISK |  | **P,T** | **P,T** |  |  | **P,T** |  | **P,T** | |  | |  | |  |  | |  |  | |  |  | |  |  | 18 | 0-2 | | 6 months | | | | CBCL | | | | |  |  |
| E-RISK |  |  |  |  |  |  |  |  | |  | |  | |  |  | |  | **S** | |  |  | |  |  | 27 | 0-1 | |  | | | | DSM5 | | | | |  |  |
| Pelotas |  |  |  |  |  |  |  |  | |  | |  | |  |  | |  |  | | **S** |  | |  |  | 18 | 0-1 | | 6 months | | | | MINI | | | | |  |  |
| Dunedin |  |  |  |  |  |  |  |  | |  | |  | |  |  | |  |  | |  |  | | **S** | **S** | 27 | 0-1 | | 12 months | | | | DSM 5 | | | | |  |  |
| Dunedin |  |  |  |  | **P,T** |  | **P,T,S** |  | | **P,T,S** | |  | | **P,S** |  | |  |  | |  |  | |  |  | 11-14 | 0-2 | |  | | | | Short CBCL | | | | |  |  |
| TEDS |  |  |  | **P** |  |  |  | **P** | |  | | **P,S,T** | |  | **P** | |  |  | | **P** |  | |  |  | 18 | 0-3 | | 3 months | | | | Conners | | | | |  |  |
| TEDS |  |  |  |  |  |  |  |  | |  | |  | |  |  | |  |  | | **S** |  | |  |  | 20 | 0-3 | | 3 months | | | | Conners | | | | |  |  |
|  |  |  |  |  |  |  |  |  |  | |  | |  | |  |  | | |  |  | |  |  |  |  | |  | | |  | | | |  | | | | |

P = parent; T = teacher; S = self.

Self-ratings for Dunedin and E-Risk at age 18 were collected via clinician assessment.

SDQ = strengths and difficulties questionnaire (Goodman, 1997); Barkley = Barkley Adult ADHD rating scale (Barkley, 2011); DAWBA = the Development and Wellbeing Assessment (Goodman et al., 2000); DSM-5 = Diagnostic and Statistical Manual 5; Conners = the Conners’ Parent Rating scale (Conners et al., 1998); CBCL = the Child Behaviour Checklist (Achenbach & Edelbrock, 1983); MINI = the Mini International Neuropsychiatric Interview (Lecrubier et al., 1997).

**Supplementary Note 3. Measures of DSM 5 diagnostic items for ADHD across the five cohorts.**

| **DSM 5 Diagnostic Criteria items** | **ALSPAC (Barkley's ADHD Scale)** | **ALSPAC (DAWBA)** | **TEDS (Conners Parent Rating Scale)** | **E-Risk (Child Behaviour Checklist)** | **Dunedin** | **Pelotas (MINI) - Translated** |
| --- | --- | --- | --- | --- | --- | --- |
| **Hyperactive/Impulsive** |  |  |  |  |  |  |
| Often fidgets with or taps hands or feet, or squirms in seat | Fidgeted with hands or feet or squirmed in seat | Do they often fidget? | Fidgets with hands or feet or squirms in seat | Fidgety or squirmy | I often feel fidgety, restless, squirmy | Do you move around in your chair or swing your hands or feet when you need to sit for a long time? |
| Often leaves seat in situations when remaining seated is expected | Left seat in classroom or other situations in which sitting was expected | Do they run or climb about when they shouldn't? | Leaves seat in classroom or in other situations in which remaining seated is expected | Very restless, has difficulty staying seated for long | I dislike quiet activities, like sitting through long meetings | Have you had difficulty sitting in class, at work or in meetings? |
| Often runs about or climbs excessively in situations where it is inappropriate (adolescents or adults may be limited to feeling restless) | Restless in the "squirmy" sense | Is it hard for them to stay sitting down for long? | Runs about or climbs excessively in situations where it is inappropriate | Impulsive or acts without thinking | I get uncomfortable sitting still; I need to get up and move | Do you feel very restless or do you move too much in situations where you should be quiet? |
| Often unable to play or take part in leisure activities quietly | Had difficulty engaging in leisure activities or doing fun things quietly | Do they find it hard to play or take part in other leisure activities without making a noise? | Has difficulty playing or engaging in leisure activities quietly | Cannot settle to anything for more than a few moments | I'm too loud or noisy | Have you been noisy or have you spoken too loudly? |
| Is often "on the go" acting as if "driven by a motor" | Was "on the go all the time" or acted as if "driven by a motor" | If they are rushing about do they find it hard to calm down when someone asks them to do so? | Is always “on the go” or acts as if driven by a motor | Seems to be 'on the go' or acts as if 'driven by a motor' | 1) I often have difficulty unwinding or relaxing  2) I'm always on the go, in a hurry, as if driven by a motor | Are you “racing at an hour” or are you acting as if you are “powered by an engine, full steam ahead”? |
| Often talks excessively | Talked excessively | Do they often go on talking even if they have been asked to stop or no-one is listening? | Talks excessively | Talks too much | I often talk too much | Have you talked too much, “like a rattle”? |
| Often blurts out answers before a question has been completed | Blurted out answers before questions had been completed | Do they often blurt out an answer before they have heard the question properly? | Blurts out answers to questions before the questions have been completed | Blurts out answers before the whole question has been asked | 1) I make "snap" decisions (too fast)  2) I can't stop when I know I should | When you talk to people, do you answer before the questions are finished or do you finish the sentences of the people before them? |
| Often has difficulty waiting his/her turn | Had difficulty awaiting turn | Is it hard for them to wait their turn? | Has difficulty waiting in lines or awaiting turn in games or group situations | Has difficulty waiting for his\her turn | I have difficulty waiting; I'm impatient | Do you have difficulty waiting for your turn or waiting in lines? |
| Often interrupts or intrudes on others (e.g., butts into conversations or games) | Interrupted or intruded on others | Do they often butt in on other people's conversations or games? | Interrupts or intrudes on others (e.g. butts into others conversations or games) | Interrupts conversations or games | 1) I jump into projects without reading the instructions  2) I'm impulsive, I act without thinking about what might happen | Have you interrupted others or meddled in other people's activities? |
| **Inattention** |  |  |  |  |  |  |
| Often fails to give close attention to details or makes careless mistakes in schoolwork, work, or other activities | Failed to give close attention to details or made careless mistakes in work | Do they often make careless mistakes or fail to pay attention to what they are supposed to be doing? | Fails to give close attention to details or makes careless mistakes in schoolwork or other activities | Doesn't pay attention to details | I often make careless mistakes, I'm not a detail person | Do you miss school or work tasks by not paying attention to details or by carelessness? |
| Often has difficulty sustaining attention in tasks or play activities | Had difficulty sustaining attention in tasks or fun activities | Do they often seem to lose interest in what they are doing? | Has difficulty sustaining attention in tasks or play activities | Short attention span | 1) I can't concentrate, my mind wanders  2) I get bored quickly  3) I often tune out when I should focus | Do you have difficulty keeping your attention on tasks such as classes, reading or long conversations? |
| Often does not seem to listen when spoken to directly | Didn't listen when spoken to directly | Do they often not listen to what people are saying to them? | Does not seem to listen to what is being said to him/her | Doesn't notice when people speak to him\her | I don't listen | Do you have difficulty concentrating on what people say, even when they are talking directly to you? |
| Often does not follow through on instructions and fails to finish schoolwork, chores or duties in the workplace | Didn't follow through on instructions and failed to finish work | Do they often not finish a job properly? | Does not follow through on instructions and fails to finish schoolwork or chores | Fails to finish things he\she starts | I often leave projects unfinished | Do you have difficulty finishing school activities, homework or work? |
| Often has difficulty organizing tasks and activities | Had difficulty organising tasks and activities | Is it often hard for them to get themselves organised to do something? | Has difficulty organising tasks and activities | Doesn't organise him\herself well | 1) I'm messy, disorganized  2) I have difficulty organizing tasks that have many steps | Do you have you had difficulty organizing yourself in tasks and activities? |
| Often avoids, dislikes, or is reluctant to engage in tasks that require sustained mental effort (such as schoolwork or homework) | Avoided, disliked or was reluctant to engage in work that required sustained mental effort | Do they often try to get out of things they would have to think a lot about e.g. homework? | Avoids, expresses reluctance about, or has difficulties engaging in tasks that require sustained mental effort (such as schoolwork or homework) | Doesn't like activities requiring a lot of attention and effort | 1) I lack self-discipline  2) I often put off tasks that require lots of effort | Do you avoid or stop doing tasks that require a lot of thought and make continuous mental effort? |
| Often loses things necessary for tasks or activities | Lost things necessary for tasks or activities | Do they often lose things they needs for school or PE? | Loses things necessary for tasks or activities | Loses things, like toys and clothes | I often misplace my wallet, keys, eyeglasses, paperwork | Have you lost items needed to perform tasks, jobs or activities? |
| Is often easily distracted | Easily distracted | Are they easily distracted? | Easily distracted by extraneous stimuli | Inattentive or easily distracted | 1) I'm easily distracted, I get side-tracked easily  2) I can't resist temptation | Do you get easily distracted by noises, your thoughts or other things when you are concentrating or trying to concentrate on something? |
| Is often forgetful in daily activities | Forgetful in daily activities | Are they often forgetful? | Forgetful in daily activities | Forgets what he\she is doing | 1) I miss deadlines, forget appointments, am often late  2) I often forget to do errands, return calls, pay bills | Do you forget to do everyday activities, tasks and obligations? |
| Response Scale | 0 (Never or rarely) 1 (Sometimes) 2 (Often) 3 (Very often) | 1 (No) 2 (A little more than others) 3 (A lot more than others) | 0 (Not true at all) 1 (Just a little bit true) 2 (Pretty much true) 3 (Very true) | 0 (not true) 1 (Somewhat or sometimes true) 2 (Very true or often true) | 0 (Absent) 1 (Present) | 0 (Not) 1 (Yes) |
| Time Frame | Past 6 months | Past 6 months | Last 3 months | Past 6 months | Past 12 months | Past 6 months |
| Absent/Present | Absent = 0/1  Present = 2/3 | Absent = 1/2  Present = 3 | Absent = 0/1  Present = 2/3 | Absent = 0/1  Present = 2 | - | - |
| Ages at which the items are phrased this way | 25 years | 8 to 16 years | 8 to 16 years | 5 to 12 years | 38 to 45 years | 21 years |

Note. Pelotas items are translated from Portuguese. This is an example of how items are phrased in each of the five cohorts. Item phrasing and number of items changed across cohorts. The ages at which these items were collected is presented in the table. Barkley = Barkley Adult ADHD rating scale (Barkley, 2011); DAWBA = the Development and Wellbeing Assessment (Goodman et al., 2000); Conners = the Conners’ Parent Rating scale (Conners et al., 1998); MINI = the Mini International Neuropsychiatric Interview (Lecrubier et al., 1997).

**Supplementary Note 4. Details of the ADHD trait measures**

Strengths and Difficulties Questionnaire (SDQ) hyperactive-inattentive subscale

The hyperactive-inattentive subscale of the SDQ consists of 5 items capturing inattentive, hyperactive and impulsive behaviours including “easily distracted, concentration wanders” and “constantly fidgeting and squirming”. The SDQ was designed for parent-ratings of children aged 4 to 17 years^26^ but has been validated for use in adulthood^1,2^, self-ratings from age 11 years and parent-ratings in 2-3 year olds. Items are endorsed on a 3-point scale: “not true”, “somewhat true” and “certainly true” (possible scores range from 0-10, higher scores represent more ADHD behaviours. For children aged 4-17 years, recommended cut-points for teacher or parent-ratings are low (0-5), slightly raised (6-7), high (8), very high (9-10)^26^. For self-ratings they are low (0-5), slightly raised (6), high (7), very high (8-10)^26^.

DSM percentage scores

The different scales used included: the Barkley Adult ADHD rating scale^28^, the Development and Wellbeing Assessment (DAWBA)^29^, the Conners’ Parent Rating scale^30^, the Child Behaviour Checklist^31^ and the Mini International Neuropsychiatric Interview^32^. Of the 18 DSM items, 9 relate to hyperactivity/impulsivity (e.g. “Is often ‘on the go’ acting as if ‘driven by a motor’”) and 9 relate to inattention (e.g. “Often fails to give close attention to details or makes careless mistakes”). At some assessments, fewer than 18 items were collected (e.g. in Dunedin) ) and occasionally more than 18 items were collected (self-ratings in E-Risk, Dunedin and TEDS at ages 18+years) to capture age related changes in trait expression (Supplementary Note 2). To facilitate comparison across cohorts despite different item counts and response categories across scales, we converted all scores to the percentage of total possible score for that cohort, at that timepoint. A score of 0 indicates no endorsement for any DSM defined symptoms and 100 indicates the highest possible score endorsed for every DSM defined symptom. For comparison of the items across cohorts, see Supplementary Note 3.

Interpretation of the DSM percentage scores

As discussed in Supplementary Note 6, there were several inconsistencies between the measurement of DSM items across cohorts. Therefore, to make the average values from each cohort comparable, we converted scores from all cohorts to the percentage of the total possible score for that cohort at each time point. Total possible scores not only differed across cohorts, but also across time points within cohorts. This is due to different numbers of items being asked at different time points (often to capture age related changes) or because the response scale changed across time points. Consequently, it is not possible to give one conversion from the percentage scores back to item numbers and not even possible to give a conversion for each cohort. Instead, here we give some examples of what the percentages from the final model would mean for specific cohorts at specific time points.

The intercept DSM percentage score for our final model was 25%. Our reference category was the TEDS cohort at age 5 years. At this time point, TEDS asked 18 items with 4 possible response categories (0 = Not true at all, 1 = Just a little bit true, 2 = Pretty much true, 3 = Very true). Therefore, percentages were calculated from a total possible score of 54. A score of 25% would mean the individual had scored 13.5. On this scale, a response of “pretty much true” or “very true” (i.e. 2 or 3) equates to a DSM defined symptom. Thus, an individual with a raw score of 13.5 could have equivalent to 6 symptoms endorsed. However their responses could also equate to 0 symptoms endorsed, if they responded to several items with “just a bit true” (i.e. 1).

Alternatively, a score of 25% in the E-Risk cohort at age 5 years would have been out of a total possible score of 36 because 18 items were asked on a 3-level response scale (0 = not true, 1 = Somewhat or sometimes true, 2 = very true or often true). A response of “very or often true” (2) is considered equivalent to a DSM defined symptom. A score of 25% would be equivalent to a raw score of 9 which could equate to 4 DSM symptoms, or again the individual could have instead endorsed several items as “somewhat or sometimes true”.

Alternatively, a score of 25% in the Pelotas cohort at age 21, was out of a total possible score of 18. Individuals were asked the DSM-5 18 items to which they could respond either 0 (no) and 1(yes). Consequently, an individual with 25% score in the Pelotas cohort at age 21 would have a raw score of 4.5, equivalent to 4.5 DSM defined symptoms out of 18.

All of the given examples related to a maximum of 4-6 DSM defined symptoms. This is below the threshold required for a DSM-5 diagnosis of ADHD, which is at least 6 inattention and 6 hyperactivity symptoms (12 total) for children up to age 16 years, or five or more of each (10 total) for adolescents age 17 years and older and adults (American Psychiatric Association, 2013).

**Supplementary Note 5: Measures of covariates across cohorts**

Sex

Sex (male or female) was self-reported at first assessment in E-Risk and TEDS. In ALSPAC, Pelotas and Dunedin, it was obtained from the birth record.

For all analyses, male was our reference category.

Birthweight

In ALSPAC, birthweight was identified using three sources: 1) obstetric data, 2) birthweight as recorded by the ALSPAC measurers and 3) birthweight from birth notification. For this analysis, we used the ‘preferred birthweight’ variable in ALSPAC which combines the data from the three sources in the following way: if all birthweights from each source were identical, that was taken. If two sources are the same, and one is different, they take the weight which is the same. If there is a discrepancy between all three sources or only two sources are available, then if the difference was less than 100g, they took the lower birthweight. If the difference was greater than 100g, then they are set to missing. In E-Risk and TEDS, each twin’s birthweight was obtained from parental recall when the twins were around one year old (Tully et al., 2004).

For Dunedin and Pelotas, birthweight was taken from medical records at the time of the infant’s birth.

We converted birthweight to kilograms for every cohort. Our models were centred to the rounded mean birthweight of 3kg.

Gestational age

In ALSPAC, gestation was recorded in a variety of ways: 1) using last menstrual period, 2) paediatric assessment, 3) obstetric assessment and 4) ultrasound assessment. We used the ALSPAC generated *bestgest* variable, which mostly used last menstrual period, if the mother was certain of it and there were no other clinical suggestions that this was erroneous. If the date of the last menstrual period was considered unreliable, then the earliest ultrasound measurement was most likely to be taken. In TEDS and E-Risk, gestational age was obtained from parent recall at first contact, when the twins were around 1 year old. For Dunedin, gestational age was from the medical chart at the time of the infant’s birth. For Pelotas, gestational age was from medical records taken within the maternity ward.

We converted gestational age to weeks for every cohort. Our models were centred to the rounded mean gestational age of 38 weeks.

Maternal age at delivery

In ALSPAC, the age of the mother at delivery was calculated from the mother’s date of birth and date of delivery. In TEDS, maternal age at delivery was reported by the mother at first contact, when the twins were aged around 1 year old. For E-Risk, we calculated mother’s age at delivery using twin date of birth and mother date of birth.

For Dunedin, mother’s age at delivery was from the medical chart at the time of the infant’s birth. For Pelotas, mother’s age at delivery was self-reported by the mothers at birth.

We converted the units of maternal age at delivery to years for every cohort. Our models were centred to the rounded mean gestational age of 30 years.

Parental socio-economic position (SEP)

For ALSPAC, we used the socio-economic group of the mother calculated from the response to occupation questions from multiple questionnaires over the first 3 years of the child’s life. Using semi-automatic processing, the responses were assigned standard occupational classification 2000 codes (SOC 2000), which were then converted to socio-economic groupings. For TEDS, we used family SEP at first contact (around age 1 year). This measure is a composite of five derived variables: maternal social class, paternal social class, maternal education qualification level, paternal education qualification level and mother's age at birth of first child. In E-Risk, we used a parental social class composite from data collected when twins were age 5 years. The standardized composite combines income, education, and occupation. The three SEP indicators were highly correlated (r ranged from 0.57 to 0.68, all p < .05) and loaded significantly onto one latent factor (factor loadings = 0.82, 0.70, and 0.83 for income, education, and occupation, respectively). In Dunedin, family SEP was measured on a six-point scale that assessed the occupational status of both parents using average income and education level derived from the New Zealand Census (Elley & Irving, 1976). Occupational status was assessed from birth, and again at subsequent assessments up to when the child was aged 15 years. The highest occupational status of either parent was averaged across the childhood assessments (Poulton et al., 2002). For Pelotas, families were categorised at child age 11 into one of five social classes from Class A being the highest to Class E being the lowest. Classification into the five classes is based upon a points system described elsewhere (Associação Brasilerira de Empresas de Pesquisa, 2016).

Due to differences in measurement of SEP across cohorts, we standardised scores for each cohort (mean = 0; SD = 1) prior to analysis, after ensuring that higher scores indicated higher SEP, and reversing any scores if necessary.

**Supplementary Note 6. Benchmark Model of DSM Differences across Cohorts**

Methods

The measurement of DSM scales was not as consistent as for SDQ measures. Inconsistencies included: different item wording and different numbers of items (often to capture age related change), different raters, different response scales, and with reference to different time frames. Therefore, our DSM model required additional steps to be able to harmonise and consequently compare across the cohorts.

To address this, first we constructed a benchmark model, using only five parent-rated items that were phrased relatively consistently across the cohorts at similar time points. These were: “*Squirms, fidgets”, “Fail to finish”, “Doesn't listen”, “Easily distracted”, “Difficulty organising tasks and activities”.* Given different scales used across cohorts, we converted the scores to a percentage of total possible score for each cohort. For simplicity of this model, we only adjusted for cohort, sex and allowed interactions between cohort and slope. We did not include related individuals in this model (excluding one twin at random in TEDS and E-Risk, and keeping first born child in ALSPAC), resulting in a two-level multi-level model. The first level being repeated measures clustered within the second level of unrelated individuals. Differences between cohorts observed in this benchmark model can be used to help interpret the overall DSM model.

Given the approach used in this benchmark model, the following were now consistent across the cohorts: rater, item phrasing, number of items, and the response scale. However, it was not possible to remove all differences. Therefore, differences between cohort could be due to differences in response scale prior to conversion to percentage of total possible score (e.g. 0 (no) 1 (yes) in Pelotas v. 0 (Not true at all) 1 (Just a little bit true) 2 (Pretty much true) 3 (Very true) in TEDS) and due to differences in timing (e.g. over the last 6 months in ALSPAC v. 3 months in TEDS) as well as true differences in trait prevalence/reporting differences across cohorts. We did not aim to interpret the coefficient estimates from this benchmark model, but rather to use the differences between cohorts to help interpret the final model of DSM scores.

Results

The best fitting model of DSM benchmark scores had linear splines with knot points at age 14 and 17 years. The average DSM percentage scores from the benchmark model are presented in Supplementary Figure S4. Apart from Pelotas, average trajectories showed a similar slope across all other cohorts. In particular, trajectory patterns were very similar between TEDS and ALSPAC which both had measures available over a larger age range. Pelotas showed a more rapid decrease in ADHD traits than the other cohorts. While most cohorts had similar slopes, there was large variation in the average intercepts for each cohort. Pelotas had the highest average scores, followed by E-Risk, Dunedin, TEDS and finally ALSPAC had the lowest average scores. Given that the trajectory slopes are similar and the intercepts different, this suggests that differences could be due to differences in the response scales used across cohorts. Of course, they could also be due to real changes in prevalence across cohorts.

**Supplementary Note 7. Sensitivity analysis to explore the role of attrition on patterns of change**

Repeated measures multi-level models (MLM) are robust to bias if data is missing at random, but not if the data is missing not at random. Individuals in the models must have completed at least one measure of ADHD score, and evidence suggests that ADHD traits in childhood predict ADHD traits in adulthood despite a general decreasing trend (Faraone et al., 2006). However, evidence from multi-informants across multiple settings suggests that there is some state-like as well as trait-like components of ADHD expression (Litson et al., 2018). Such state-like fluctuations in ADHD behaviours will bias MLM models if: 1) the fluctuations could not be predicted by observed variables included in the model and 2) the fluctuations make individuals less likely to take participate at that time point. Therefore, to test the possible influence of attrition on the models, we conducted a further sensitivity analysis. We explored whether the overall pattern of change differed when only including individuals who had completed items from early, mid and late stages of the trajectory.

Methods

Participants had to have completed at least one ADHD measure from the early years (E), middle years (M) and later years (L). This one response could be either parent-rated, teacher-rated or self-rated. Ages were categorised as early, middle or late separately for each cohort, to ensure that we had participants responding across the possible age range for that cohort. Roughly, early was categorised as age 0-10, middle age 10-18 and late age 18+ years. The categories used are displayed in Table N1. The Pelotas cohort only had a maximum of two time points for SDQ and only one time point for DSM, so we required participants must have responded at those time points.

**Table N1. Required time points for inclusion in the attrition sensitivity model.**

|  | **ALSPAC** | | | **TEDS** | | | **Pelotas** | | **E-Risk** | | | **Dunedin** | | |
| --- | --- | --- | --- | --- | --- | --- | --- | --- | --- | --- | --- | --- | --- | --- |
| **SDQ** | **E** | **M** | **L** | **E** | **M** | **L** | **M** | **L** |  |  |  |  |  |  |
|  | 4,7,8 | 10,12,13 | 17,25 | 4,7 | 9,12 | 16,21 | 11 | 15 |  |  |  |  |  |  |
| **DSM** | **E** | **M** | **L** | **E** | **M** | **L** |  | **L** | **E** | **M** | **L** | **E** | **M** | **L** |
|  | 8,11 | 14,16 | 25 | 8,12 | 14,16 | 21 |  | 21 | 5,7 | 10,12 | 18 | 9,11 | 13,15 | 38,45 |

*Note. Participants had to have completed at least one ADHD measure from each of the sections: early years (E), middle years (M) and later years (L), to be included in analysis. SDQ = hyperactivity subscale of the strengths and difficulties questionnaire. DSM = measures of the Diagnostic and Statistical Manual diagnosis of ADHD.*

After restricting the sample to those who had responded at multiple time points, we re-ran the best fitting models from the full dataset, to explore whether or not the trajectories were altered.

Results

After restricting to individuals who had responded at least once in early, middle and late years, we had a total of 138,024 observations from 18,240 participants in our model of SDQ (n = 5,295 ALSPAC; 8,788 TEDS; 4,157 Pelotas) and a total of 91,709 observations from 17,341 participants in our model of DSM (n = 4,971 ALSPAC; 7,540 TEDS; 2,042 E-Risk; 893 Dunedin; 3,517 Pelotas). Average trajectories for the best fitting model are given in Supplementary Figures S9 and S10. Overall pattern of change was highly consistent.

**Supplementary Note 8. The results of sex split analysis for each model**

SDQ scores

The best fitting model of SDQ scores had knot points at age 8 and 16 years. After splitting the dataset into males and females, we tested the fit of this model and iteratively removed covariates to find the most parsimonious model (Supplementary Tables S12 and S13). All covariates were retained in the model, apart from gestational age and an interaction between cohort and birthweight (i.e. the association of birthweight with SDQ appeared to be constant across cohorts).

Separate trajectories by sex are shown in Supplementary Figure S7. At all ages, average SDQ scores were lower for females than males. Our final model prediction for a male aged 3 years from the ALSPAC cohort (birthweight = 3kg, mothers age at delivery = 30 years) would have a parent-rated SDQ score of 4.32 (95% CI = 4.24, 4.40). The first spline, from ages 3 to 8 was characterised by a rapid decrease in average SDQ score such that on average, SDQ decreased by -0.12 every year (95% CI = -0.14, 0.10). The second spline, from age 8 to 16 years is characterised by a more gradual reduction in scores with a reduction of -0.07 SDQ score per year on average (95% CI = -0.09, -0.06). Predictions over the final spline (age 16 years onwards) showed the most rapid rate of trait decline, reducing by -0.15 SDQ score every year (95% CI = -0.16, -0.13).

Our final model for a female aged 3 years from the ALSPAC cohort (birthweight = 3kg, mothers age at delivery = 30 years), would have a parent-rated SDQ score of 3.80 (95% CI = 3.72, 3.88). The first spline, from ages 3 to 8 was characterised by an even more rapid decrease in average SDQ score than males, such that on average, SDQ decreased by -0.20 every year (95% CI = -0.22, -0.18). The second spline, from age 8 to 16 years is characterised by a more gradual reduction in scores with a reduction of -0.06 SDQ score per year on average (95% CI = -0.07, -0.05), similar to that observed for males. Average values over the final spline (age 16 onwards) declined less rapidly than for males: by -0.09 SDQ score every year (95% CI = -0.10, -0.07).

Adding an interaction term between sex and cohort in the main model of SDQ did not improve model fit, suggesting sex differences were consistent across cohorts.

DSM percentage scores

The best fitting model of DSM percentage scores had knot points at age 14, 17 and 21 years. After splitting the dataset into males and females, we tested the fit of this model and iteratively removed covariates to find the most parsimonious model (Supplementary Tables S22 and S23). All covariates were retained in the model, apart from gestational age and an interaction between cohort and birthweight (i.e. the association of birthweight with DSM appeared to be constant across cohorts).

Separate trajectories by sex are shown in Supplementary Figure S8. At all ages, average DSM percentage scores were lower for females than males. Our final model prediction, for a male aged 5 years from the TEDS cohort (birthweight = 3kg, mothers age at delivery = 30 years) would have a parent-rated DSM score of 26.62% (95% CI = 25.88, 27.36). The first spline, from ages 5 to 14 was characterised by a decrease in average DSM score such that on average, DSM decreased by -0.76% every year (95% CI = -0.86, -0.66). The second spline, from age 14 to 17 years is characterised by the most rapid reduction of -1.80% per year on average (95% CI = -2.04, -1.56). Over the third spline (ages 17 to 21 years), average DSM scores increased slightly by 0.39% each year on average (95% CI = 0.18, 0.60). Over the final spline (age 21 years and onwards) average DSM score decreased again by an average of -0.91% per year (95% CI = -1.28, -0.53).

Our final model prediction, for a female aged 5 years from the TEDS cohort (birthweight = 3kg, mothers age at delivery = 30 years) would have a parent-rated DSM score of 18.85% (95% CI = 18.25, 19.45). The first spline, from ages 5 to 14 was characterised by a decrease in average DSM score similar to that observed for males, such that on average, DSM decreased by -0.63% every year (95% CI = -0.71, -0.55). The second spline, from age 14 to 17 years is characterised by the most rapid reduction with a reduction, although less rapid than for males, of -0.94% per year on average (95% CI = -1.13, -0.76). Over the third spline (ages 17 to 21 years), similar to observed for males, DSM scores increased slightly by 0.49% each year on average (95% CI = 0.32, 0.66). Over the final spline (age 21 years and onwards) average DSM score decreased again, similar to males, by an average of -0.80% per year (95% CI = -1.11, -0.49).

For the full DSM model, adding an interaction term between sex and cohort significantly improved model fit, suggesting sex differences across cohorts. Model coefficients (Supplementary Table S25) showed that male scores were higher than female scores on average for all cohorts, apart from the Pelotas cohort (at age 21 years) where female scores were 0.82% higher.

**Supplementary Note 9. The results of SEP split analysis for each model**

SDQ scores

The best fitting model of SDQ scores had knot points at age 8 and 16 years. After splitting the dataset into high and low SEP groups, we tested the fit of this model and iteratively removed covariates to find the most parsimonious model (Supplementary Tables S14 and S15). The best fitting model for low SEP was adjusted for cohort, rater, birthweight, mother's age at delivery and sex, and includes interactions between cohort and rater, sex and slope. The best fitting model for high SEP was adjusted for cohort, rater, birthweight, mother's age at delivery and sex, and includes interactions between cohort and rater, mother's age at delivery and slope.

Separate trajectories by SEP are plotted in Supplementary Figure S9. Given that the best fitting covariates were different for low and high SEP groups, here we included all covariates important for both models to make them comparable. This model is adjusted for cohort, rater, birthweight, mother’s age at delivery and sex, and it includes interactions between cohort and rater, age at delivery, sex and slope. At all ages, average SDQ scores were lower for the high SEP group than the low SEP group. Slope was very similar across the groups.

Our final model prediction for a male from the low SEP group, aged 3 years from the ALSPAC cohort (with average covariates) would have a parent-rated SDQ score of 5.65 (95% CI = 5.48, 5.81). The first spline, from ages 3 to 8 was characterised by the fastest rate of decline such that on average, SDQ decreased by -0.16 every year (95% CI = -0.19, -0.13). The second spline, from age 8 to 16 years is characterised by a more gradual reduction of -0.07 SDQ score per year on average (95% CI = -0.09, -0.05). Predictions over the final spline (age 16 years onwards) again declined more rapidly, reducing by -0.11 SDQ score every year (95% CI = -0.13, -0.08).

Our final model for a male from the high SEP group, aged 3 years from the ALSPAC cohort (with average covariates), would have a parent-rated SDQ score of 4.82 (95% CI = 4.69, 4.95). In the first spline, from ages 3 to 8, SDQ decreased by -0.12 every year (95% CI = -0.13, -0.10). The second spline, from age 8 to 16 years was characterised by a more gradual reduction of -0.06 SDQ score per year on average (95% CI = -0.07, -0.04), similar to that observed for the low SEP group. Average values over the final spline (age 16 onwards) declined more rapidly by -0.11 SDQ score every year (95% CI = -0.12, -0.10).

DSM percentage scores

The best fitting model of DSM scores had knot points at age 14, 17 and 21 years. After splitting the dataset into low and high SEP groups, we tested the fit of this model and iteratively removed covariates to find the most parsimonious model (Supplementary Tables S26 and S27). For the low SEP group, all covariates were retained in the model, apart from birthweight, gestational age and an interaction between cohort and age at delivery (i.e. the association of age at delivery with DSM appeared to be constant across cohorts). For the high SEP group, all covariates were retained in the model, apart from gestational age and an interaction between cohort and birthweight.

Separate trajectories by SEP group are shown in Supplementary Figure S10. Given that the best fitting covariates were different for low and high SEP groups, in the final model we included all covariates important for both models to ensure they are comparable. This model is adjusted for cohort, rater, birthweight, mother’s age at delivery and sex. It includes interactions between cohort and rater, sex, mother’s age at delivery and slope. Across the first 3 splines (from ages 5 to 21 years) average DSM percentage scores were higher for the low SEP group compared to the high SEP group. In the final spline (age 21 years onwards) it appears that the average scores converge (although confidence intervals are large).

Our final model prediction, for a male from the low SEP aged 5 years from the TEDS cohort (with average covariates) would have a parent-rated DSM score of 27.13% (95% CI = 26.25, 28.01). From ages 5 to 14, DSM score decreased on average by -0.69% every year (95% CI = -0.80, -0.58). From ages 14 to 17 years, DSM scores decreased most rapidly by -1.41% per year on average (95% CI = -1.68, -1.15). From ages 17 to 21 years, DSM scores increased slightly by 0.16% each year on average (95% CI = -0.07, 0.40). From age 21 years and onwards, DSM score decreased again by an average of -0.92% per year (95% CI = -1.33, -0.51).

Our final model prediction, for a male from the high SEP group aged 5 years from the TEDS cohort (with average covariates) would have a parent-rated DSM score of 23.16% (95% CI = 22.50, 23.82). From ages 5 to 14 years, DSM score decreased by -0.71% every year (95% CI = -0.79, -0.64). From ages 14 to 17 years, there was the largest average reduction of -1.24% per year on average (95% CI = -1.41, -1.07). From ages 17 to 21 years, DSM scores increased slightly by 0.55% each year on average (95% CI = 0.40, 0.70). Over the final spline (age 21 years and onwards) average DSM score decreased again by an average of -0.63% per year (95% CI = -0.91, -0.35).

**Supplementary Note 10. Exploration of the average increase in DSM score between ages 17 and 21**

In the third spline of the DSM model (ages 17 to 21) there is an average increase in DSM scores. We conducted additional exploratory analyses to see if this is likely a methodological artifact. There are only two cohorts with observed DSM scores between 17 and 21: TEDS at age 21 and E-Risk at age 18. Both cohorts include self- as well as parent-reports at that age.

For TEDS, self-ratings at age 21 have 20 items instead of 18. Two items are split down into a further two items, for example, *“Often fails to give close attention to details or makes careless mistakes in schoolwork, work, or other activities”*, becomes 1) *“It is hard for me to pay attention to details”* and 2) *“I make mistakes by accident”*. Parent ratings at age 21 remain with the same 18 original items.

For E-Risk, there is a change of informant at age 18 from parents and teachers to self. However, informant is accounted for as a covariate in the model so this should not be accounting for the increase in scores. There is also a change in scoring at age 18. All previous assessments are scored 0-2. At age 18, self-ratings are coded yes/no from clinical interview.

To explore whether this increase is likely methodological, we removed self-rated DSM scores at age 21 years from the TEDS cohort, leaving only parent-ratings at this age. The measurement of parent-ratings was consistent with previous ages, so an associated change in average scores suggests that it may previously have been due to change in measurement. We were unable to explore E-Risk scores at 18 in the same way because only self-ratings were available. We were also unable to include a fixed effect for number of response categories or for number of items because those were highly correlated with age.

Results

In the original DSM model, between 17 and 21 years, DSM score increased by 0.46% (95% CI: 0.32, 0.60) per year increase in age. After removing TEDS self-ratings at age 21, the trajectory was similar, increasing by 0.49% (95% CI: 0.35, 0.63) per year increase in age. Model results are show in Supplementary Figure S11.

**Supplementary Note 11. Additional Methodological Details**

*Model Fitting.* First, the cohort with the greatest spread of parent-rated repeated measures (the model development cohort) was used to test different models of non-linear change separately for SDQ (ALSPAC) and DSM percentage scores (TEDS). At this initial stage, we only included unrelated individuals and parent-ratings for simplicity. We fit a two-level MLM with repeated measures of SDQ/DSM (level 1) nested within participants (level 2). We allowed for individual variation in both initial ADHD scores (random intercept) and longitudinal pattern (random slope). We used a cubic spline model to identify the overall shape of the growth curve. To aid model interpretation and parsimony, we simplified with linear splines and knot points, while maintaining good fit. A linear spline model assumes that change takes place in periods of linear change joined at knot points^16^. AIC was used to determine the best fitting number of splines and the location of the knot points. Subsequently, we included data from all raters (parent, teacher and self) in the best fitting model, adding a fixed effect categorical covariate for rater, to allow each trait’s mean score to differ by informant.

We then assessed the fit of the best-fitting model in each of the other cohorts, and in all cohorts combined (adding a fixed effect categorical covariate for cohort). The five additional covariates were then added to the model (sex, birthweight, gestational age, maternal age at delivery and SEP) as fixed effects and also as interaction terms with cohort, to allow the influence of each covariate on slope to differ by cohort. We assessed the change in fit after removing each of these covariates iteratively to achieve the most parsimonious model. We added related individuals to the dataset (twin pairs for TEDS and E-Risk, siblings/twins for ALSPAC) and accounted for the greater similarity between these individuals by including a third level (family) to the model. The final MLM is presented in Figure 1. To compare trajectories for males and females, we also modelled sex stratified trajectories.

Family

Level 3

Individual

Level 2

Repeated measures

Level 1

*Figure 1. The final hierarchical multi-level model, with repeated measures of ADHD traits nested within individuals who are nested within families.*

All analyses used a one stage approach with access to individual-level participant data.

*Model centring.* We centred each of the covariates to improve model interpretation. Age was centred at the earliest round age available (3 years for SDQ and 5 years for DSM). Continuous covariates were centred to the rounded overall mean (birthweight - 3kg, gestational age - 38 weeks, maternal age at delivery - 30 years). Primary results reported are from this mean centred model. To test for the impact of using average cohort means, we compared the results with a model where each cohort’s covariates were centred at the mean for that cohort.

**References**

Achenbach, T. M., & Edelbrock, C. S. (1983). *Manual for the child behaviour checklist and revised child behaviour profile.* 393–405.

American Psychiatric Association. (2013). Diagnostic and statistical manual of mental disorders, 5th edition. *American Psychiatric Association*, *21*.

Associação Brasilerira de Empresas de Pesquisa. (2016). *Critério de Classificação Econômica Brasil: Brazilian Criteria 2015 and social class distribution update for 2016*.

Barkley, R. A. (2011). *Barkley Adult ADHD Rating Scale-IV (BAARS-IV)*. Guilford Press.

Boyd, A., Golding, J., Macleod, J., Lawlor, D. A., Fraser, A., Henderson, J., Molloy, L., Ness, A., Ring, S., & Davey Smith, G. (2013). Cohort profile: The ‘children of the 90s’—the index offspring of the Avon Longitudinal Study of Parents and Children. *International Journal of Epidemiology*, *42*(1), 111–127.

Conners, C. K., Sitarenios, G., Parker, J. D., & Epstein, J. N. (1998). The revised Conners’ Parent Rating Scale (CPRS-R): Factor structure, reliability, and criterion validity. *Journal of Abnormal Child Psychology*, *26*(4), 257–268.

Elley, W., & Irving, J. (1976). Revised socio-economic index for New Zealand. *New Zealand Journal of Educational Studies*, *7*, 153–167.

Faraone, S. V., Biederman, J., & Mick, E. (2006). The age-dependent decline of attention deficit hyperactivity disorder: A meta-analysis of follow-up studies. *Psychological Medicine*, *36*(2), 159.

Fraser, A., Macdonald-Wallis, C., Tilling, K., Boyd, A., Golding, J., Davey Smith, G., Henderson, J., Macleod, J., Molloy, L., Ness, A., & others. (2012). Cohort profile: The Avon Longitudinal Study of Parents and Children: ALSPAC mothers cohort. *International Journal of Epidemiology*, *42*(1), 97–110.

Gonçalves, H., Wehrmeister, F. C., Assunção, M. C. F., Tovo-Rodrigues, L., Oliveira, I. O. de, Murray, J., Anselmi, L., Barros, F. C., Victora, C. G., & Menezes, A. M. B. (2018). Cohort Profile Update: The 1993 Pelotas (Brazil) Birth Cohort follow-up at 22 years. *International Journal of Epidemiology*, *47*(5), 1389–1390e. https://doi.org/10.1093/ije/dyx249

Goodman, R. (1997). The Strengths and Difficulties Questionnaire: A research note. *Journal of Child Psychology and Psychiatry, and Allied Disciplines*, *38*(5), 581–586. https://doi.org/10.1111/j.1469-7610.1997.tb01545.x

Goodman, Robert, Ford, T., Richards, H., Gatward, R., & Meltzer, H. (2000). The development and well-being assessment: Description and initial validation of an integrated assessment of child and adolescent psychopathology. *Journal of Child Psychology and Psychiatry*, *41*(5), 645–655.

Harris, P. A., Taylor, R., Minor, B. L., Elliott, V., Fernandez, M., O’Neal, L., McLeod, L., Delacqua, G., Delacqua, F., Kirby, J., Duda, S. N., & REDCap Consortium. (2019). The REDCap consortium: Building an international community of software platform partners. *Journal of Biomedical Informatics*, *95*, 103208. https://doi.org/10.1016/j.jbi.2019.103208

Harris, P. A., Taylor, R., Thielke, R., Payne, J., Gonzalez, N., & Conde, J. G. (2009). Research electronic data capture (REDCap)—A metadata-driven methodology and workflow process for providing translational research informatics support. *Journal of Biomedical Informatics*, *42*(2), 377–381. https://doi.org/10.1016/j.jbi.2008.08.010

Haworth, C. M. A., Davis, O. S. P., & Plomin, R. (2013). Twins Early Development Study (TEDS): A Genetically Sensitive Investigation of Cognitive and Behavioral Development From Childhood to Young Adulthood. *Twin Research and Human Genetics : The Official Journal of the International Society for Twin Studies*, *16*(1), 117–125. https://doi.org/10.1017/thg.2012.91

Kovas, Y., Haworth, C. M., Dale, P. S., Plomin, R., Weinberg, R. A., Thomson, J. M., & Fischer, K. W. (2007). The genetic and environmental origins of learning abilities and disabilities in the early school years. *Monographs of the Society for Research in Child Development*, *72*(3), 1–156.

Lecrubier, Y., Sheehan, D. V., Weiller, E., Amorim, P., Bonora, I., Sheehan, K. H., Janavs, J., & Dunbar, G. C. (1997). The Mini International Neuropsychiatric Interview (MINI). A short diagnostic structured interview: Reliability and validity according to the CIDI. *European Psychiatry*, *12*(5), 224–231.

Litson, K., Geiser, C., Burns, G. L., & Servera, M. (2018). Trait and state variance in multi-informant assessments of ADHD and academic impairment in Spanish first-grade children. *Journal of Clinical Child & Adolescent Psychology*, *47*(5), 699–712.

Moffitt, T. E. & E-Risk Study Team. (2002). Teen-aged mothers in contemporary Britain. *Journal of Child Psychology and Psychiatry, and Allied Disciplines*, *43*(6), 727–742. https://doi.org/10.1111/1469-7610.00082

Northstone, K., Lewcock, M., Groom, A., Boyd, A., Macleod, J., Timpson, N., & Wells, N. (2019). The Avon Longitudinal Study of Parents and Children (ALSPAC): An update on the enrolled sample of index children in 2019. *Wellcome Open Research*, *4*. https://doi.org/10.12688/wellcomeopenres.15132.1

Poulton, R., Caspi, A., Milne, B. J., Thomson, W. M., Taylor, A., Sears, M. R., & Moffitt, T. E. (2002). Association between children’s experience of socioeconomic disadvantage and adult health: A life-course study. *The Lancet*, *360*(9346), 1640–1645.

Poulton, R., Moffitt, T. E., & Silva, P. A. (2015). The Dunedin Multidisciplinary Health and Development Study: Overview of the first 40 years, with an eye to the future. *Social Psychiatry and Psychiatric Epidemiology*, *50*(5), 679–693. https://doi.org/10.1007/s00127-015-1048-8

Tully, L. A., Arseneault, L., Caspi, A., Moffitt, T. E., & Morgan, J. (2004). Does maternal warmth moderate the effects of birth weight on twins’ attention-deficit/hyperactivity disorder (ADHD) symptoms and low IQ? *Journal of Consulting and Clinical Psychology*, *72*(2), 218–226. https://doi.org/10.1037/0022-006X.72.2.218

Victora, C. G., Hallal, P. C., Araújo, C. L., Menezes, A. M., Wells, J. C., & Barros, F. C. (2008). Cohort Profile: The 1993 Pelotas (Brazil) Birth Cohort Study. *International Journal of Epidemiology*, *37*(4), 704–709. https://doi.org/10.1093/ije/dym177
